# A universal hydrochloric acid-assistant powder-to-powder strategy for quick and mass preparation of lead-free perovskite microcrystals

**DOI:** 10.1038/s41377-023-01117-2

**Published:** 2023-03-20

**Authors:** Huanxin Yang, Xiangxiang Chen, Yiyue Chu, Changjiu Sun, Haolin Lu, Mingjian Yuan, Yuhai Zhang, Guankui Long, Libing Zhang, Xiyan Li

**Affiliations:** 1 Institute of Photoelectronic Thin Film Devices and Technology, Solar Energy Conversion Center, Nankai University, Tianjin, 300350 China; 2 Key Laboratory of Photoelectronic Thin Film Devices and Technology of Tianjin, Nankai University, Tianjin, 300350 China; 3 Engineering Research Center of Thin Film Photoelectronic Technology of Ministry of Education, Nankai University, Tianjin, 300350 China; 4 Collaborative Innovation Center of Technology and Equipment for Biological Diagnosis and Therapy in Universities of Shandong, Institute for Advanced Interdisciplinary Research (IAIR), University of Jinan, Jinan, 250022 Shandong China; 5 Tianjin Key Laboratory of Molecular Optoelectronic, Department of Chemistry, Tianjin University, Tianjin, 300354 China; 6 Key Laboratory of Advanced Energy Materials Chemistry (Ministry of Education), Renewable Energy Conversion and Storage Center (RECAST), College of Chemistry, Nankai University, Tianjin, 300071 China; 7 School of Materials Science and Engineering, Nankai University, Tianjin, 300350 China

**Keywords:** Inorganic LEDs, Optical spectroscopy

## Abstract

Lead-free halide perovskite materials possess low toxicity, broadband luminescence and robust stability compared with conventional lead-based perovskites, thus holding great promise for eyes-friendly white light LEDs. However, the traditionally used preparation methods with a long period and limited product yield have curtailed the commercialization of these materials. Here we introduce a universal hydrochloric acid-assistant powder-to-powder strategy which can accomplish the goals of thermal-, pressure-free, eco-friendliness, short time, low cost and high product yield, simultaneously. The obtained Cs_2_Na_0.9_Ag_0.1_In_0.95_Bi_0.05_Cl_6_ microcrystals exhibit bright self-trapped excitons emission with quantum yield of (98.3 ± 3.8)%, which could retain (90.5 ± 1.3)% and (96.8 ± 0.8)% after continuous heating or ultraviolet-irradiation for 1000 h, respectively. The phosphor converted-LED exhibited near-unity conversion efficiency from ultraviolet chip to self-trapped excitons emission at ~200 mA. Various ions doping (such as Cs_2_Na_0.9_Ag_0.1_InCl_6_:Ln^3+^) and other derived lead-free perovskite materials (such as Cs_2_ZrCl_6_ and Cs_4_MnBi_2_Cl_12_) with high luminous performance are all realized by our proposed strategy, which has shown excellent availability towards commercialization.

## Abstract (web summary)

A general hydrochloric acid-assistant powder-to-powder strategy was proposed for preparing a series of lead-free perovskite microcrystals, meeting the needs of rapid preparation, mass production and remarkable fluorescent performance, simultaneously.

## Graphical abstract

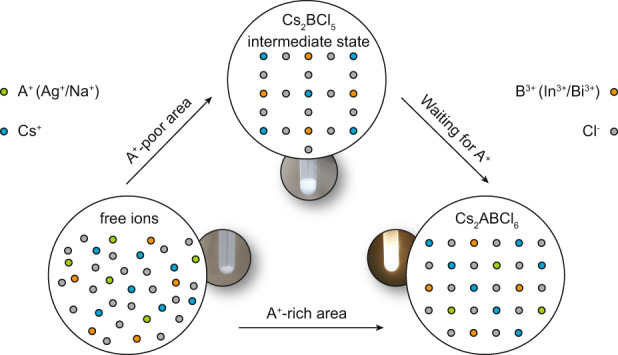

## Introduction

Lead-free halide perovskite materials are considered as one of the most competitive luminescence candidates for greatly overcoming toxicity and instability of conventional lead-based halide perovskites APbX_3_ (A = Cs, MA or FA, etc. X = Cl, Br, I)^1–6^. The efficient broadband emission induced by self-trapped excitons (STEs) empowers them with unique characteristics in applications such as eyes-friendly white light LEDs or background light sources of LCD screens^7–13^. For example, the bright warm-white light from single component Cs_2_Na_1−*x*_Ag_*x*_In_1−*y*_Bi_*y*_Cl_6_ can greatly reduce the complexity of LED device structure and avoid the inefficient reabsorption between multiple powders^10,14–16^. Furthermore, the flexible luminescence regulation supported by various ions-doping makes them suitable for different application scenarios, such as indoor lighting, bio-imaging and up-conversion anti-counterfeiting^10,17–21^, etc. Lead-free perovskite materials are expected to be commercialized due to their promising luminescence performance and wide range of applicability.

A synthesis strategy that can realize excellent photoluminescence (PL) performance, eco-friendly low cost, and rapid mass production is a prerequisite for achieving the goal of industrial applications. However, conventional strategies such as hydrothermal and solid-state reactions cannot greatly meet the needs of industrialization due to the considerable time cost (hour scale) and unsafe high reaction temperature (over 180 °C) or high pressure (over 1 MPa)^18,22,23^, etc. In contrast, the precipitation method is more popular for preparing lead-free perovskites due to the rapid reaction rate and bright luminescence performance of the products. Taking chloric double perovskite Cs_2_(Ag/Na)InCl_6_ as an example, essential raw materials such as CsCl, AgCl and NaCl are firstly dissolved in the selected regents (e.g. hydrochloric acid, called HCl hereafter) separately to form precursors, and then the precipitations are produced by proportionally mixing different precursors together^12^. The concentrated HCl is thought as a decent solvent because of the Cl^−^-rich environment for passivation of surface vacancies^24,25^. Unfortunately, the solubility of some raw materials such as AgCl, NaCl or ZrCl_4_ are limited in concentrated HCl solution, leading to a large consumption of HCl with limited yield of products (e.g. 30 mL concentrated HCl was consumed for preparing only 1 mmol product)^26^. In 2017, Volonakis et al. increased reaction temperature to 115 °C to enhance the solubility of AgCl in concentrated HCl^27^; Majher et al. and Wang et al. synthesized Cs_2_NaBiCl_6_:Mn^2+^ and Cs_2_Ag_0.4_Na_0.6_InCl_6_:Bi^3+^,Ce^3+^ microcrystals at 80 °C in 2019 and 2020, respectively^7,25^. However, it should be noted that HCl gas may be volatilized above the boiling point of concentrated HCl (~45–48 °C), leading to decreased product yield and inferior PL properties (Fig. 2g in the following text). In addition, the introduction of condensing units and hour-scale preparation may increase the economic costs, limiting the commercialization. Up to date, there is still a lack of preparation strategy that could satisfy the requirements of thermal-, pressure-free, eco-friendliness, short time, low cost, as well as high product yield, simultaneously.

Herein, taking Cs_2_Na_1−*x*_Ag_*x*_In_1−*y*_Bi_*y*_Cl_6_ as an example, we would like to introduce a universal concentrated hydrochloric acid-assistant powder-to-powder (HAAPP) strategy for preparing lead-free perovskite microcrystals, which can simultaneously satisfy the above-mentioned requirements. The products can be obtained by simply mixing the raw materials in a small amount of concentrated HCl solution, and the obtained uniform phase structure and high luminescence performance, as well as great alloying effect imply that complete dissolution of the raw materials seems unnecessary in recrystallization method. The firstly obtained intermediate product Cs_2_In_1-*y*_Bi_*y*_Cl_5_·H_2_O would combine the gradually released Na^+^/Ag^+^ from NaCl/AgCl to grow to the final Cs_2_Na_1-*x*_Ag_*x*_In_1-*y*_Bi_*y*_Cl_6_ within several minutes. Benefited from the Cl^−^-rich environment of concentrated HCl, the products synthesized by HAAPP strategy showed remarkable product yield of ~90% and near-unity PL quantum yield (PLQY). Although synthesized at room temperature, the product retained ~72.22% as intensity as 123 K while heating to 423 K. After continuous heating or ultraviolet (UV) irradiation, the products still retained (90.5 ± 1.3)% and (96.8 ± 0.8)% of PLQY, respectively. The near-unity conversion efficiency from UV-LED chip to STE emission indicated an excellent application value for warm-white LEDs. Furthermore, our proposed HAAPP strategy can be greatly applied to the other lead-free perovskite structures, such as CsMnCl_3_, vacancy-order perovskite Cs_2_ZrCl_6_, layered double perovskite Cs_4_MnBi_2_Cl_12_, or Br-/I-based perovskites Cs_2_AgBiBr_6_/Cs_3_Bi_2_I_9_, etc. The universal powder-to-powder synthesis strategy will provide a reliable way for the development of lead-free perovskite materials and industrialization.

## Results

### Synthesis strategy

**Fig. 1:**
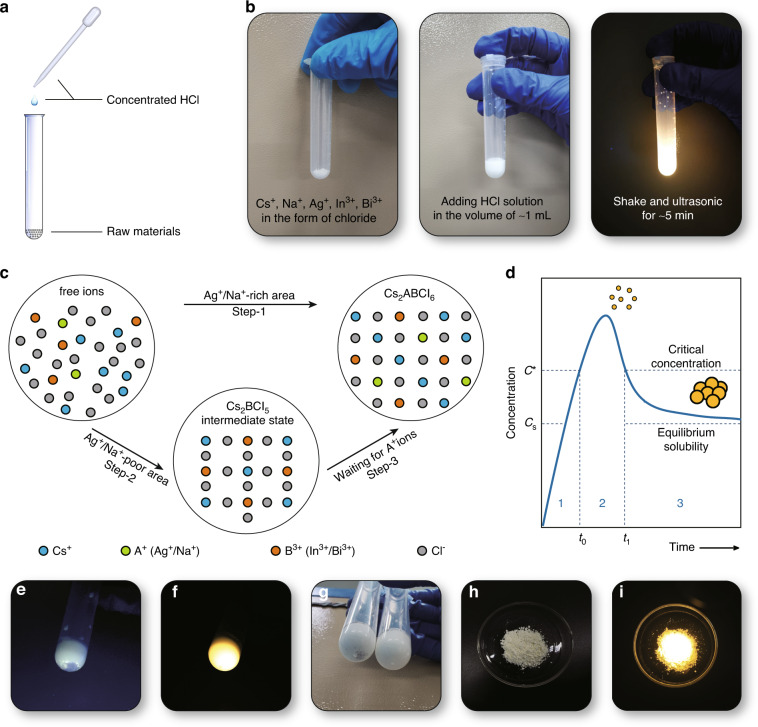
HAAPP synthesis strategy. **a** Operation diagram of the HAAPP strategy; **b** Digital photographs of the sample in each step; **c** Mechanism description about the growth processes of the HAAPP strategy; **d** LaMer model for description of the crystal growth processes; Digital photographs of the samples **e** once adding concentrated HCl and **f** after shaking for ~5 min; **g** Comparison of products with (left) and without (right) UV irradiation during synthesis; Digital photographs of the products obtained by mass preparation under **h** room light and **i** 365 nm UV light

**Fig. 2:**
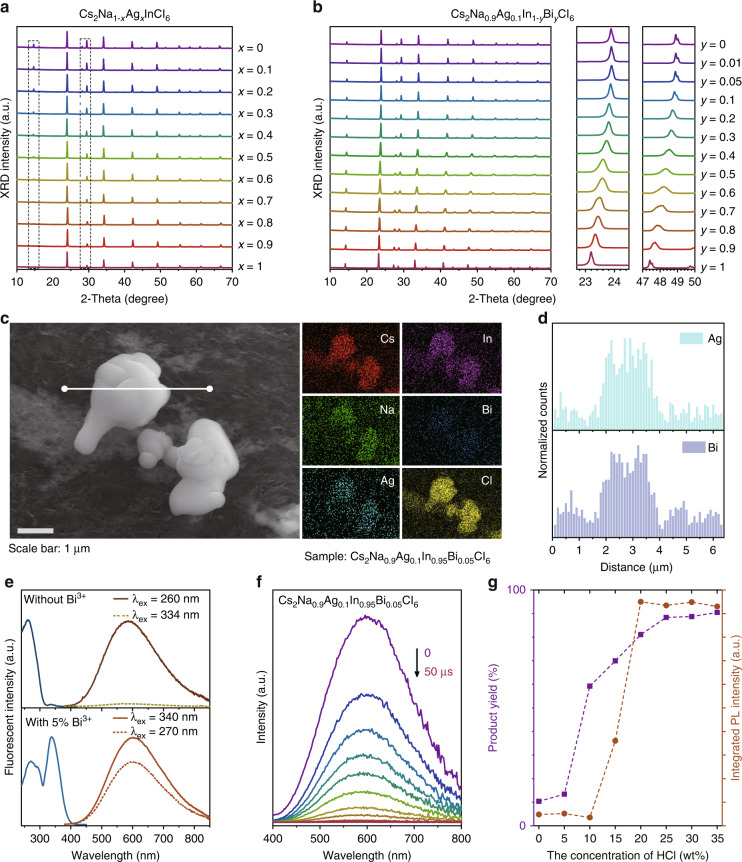
Phase and luminous performance of the synthesized products. XRD patterns of the **a** Cs_2_Na_1-*x*_Ag_*x*_InCl_6_ and **b** Cs_2_Na_0.9_Ag_0.1_In_1-*y*_Bi_*y*_Cl_6_; **c** SEM image and component elements maps of Cs_2_Na_0.9_Ag_0.1_In_0.95_Bi_0.05_Cl_6_; **d** EDS profile of the line scanning marked in (**c**); **e** PLE ( < 400 nm) and PL (380–850 nm) spectra of Cs_2_Na_0.9_Ag_0.1_InCl_6_ and Cs_2_Na_0.9_Ag_0.1_In_0.95_Bi_0.05_Cl_6_, respectively; **f** Microsecond transient emission spectra of Cs_2_Na_0.9_Ag_0.1_In_0.95_Bi_0.05_Cl_6_ excited at 260 nm; **g** Product yields and corresponding PL intensities of Cs_2_Na_0.9_Ag_0.1_In_0.95_Bi_0.05_Cl_6_ as a function of the concentration of HCl

The lead-free double perovskite Cs_2_Na_0.9_Ag_0.1_In_0.95_Bi_0.05_Cl_6_ with microcrystal morphology was successfully synthesized by our proposed HAAPP strategy, which was simply adding a small amount of concentrated HCl to the mixed raw materials, as shown in Fig. 1a and Video S1. After shaking and ultrasonication for ~5 min, the mixture emitted bright yellow light efficiently (Fig. 1b) under 365 nm UV irradiation. X-ray diffraction (XRD) (Fig. 2) and transmission electron microscope (TEM, Supplementary Fig. S1) were conducted to confirm the formation of the Cs_2_Na_0.9_Ag_0.1_In_0.95_Bi_0.05_Cl_6_. Specifically, the ions from the raw materials are firstly released to the solution, shown as Eqs. (1)-(5):

1 $${{{\mathrm{CsCl}}}} = {{{\mathrm{Cs}}}}^ + + {{{\mathrm{Cl}}}}^ -$$

2 $${{{\mathrm{InCl}}}}_3 = {{{\mathrm{In}}}}^{3 + } + 3{{{\mathrm{Cl}}}}^ -$$

3 $${{{\mathrm{BiCl}}}}_3 = {{{\mathrm{Bi}}}}^{3 + } + 3{{{\mathrm{Cl}}}}^ -$$

4 $${{{\mathrm{AgCl}}}} \rightleftharpoons {{{\mathrm{Ag}}}}^ + + {{{\mathrm{Cl}}}}^ -$$

5 $${{{\mathrm{NaCl}}}} \rightleftharpoons {{{\mathrm{Na}}}}^ + + {{{\mathrm{Cl}}}}^ -$$

Among them, AgCl and NaCl actually show extremely poor solubility in concentrated HCl, causing partially dissolved Ag^+^ and Na^+^ ions in solution. Then, the final product of Cs_2_ABCl_6_ (“A^+^” represents monovalent ions Na^+^ or Ag^+^, while “B^3+^” represents triple valent ions In^3+^ or Bi^3+^) can be synthesized in one step in the Ag^+^/Na^+^-rich area, which show local bright luminescence, shown in Fig. 1c (Step-1), Eq. (6) and Supplementary Fig. S2. Otherwise, the intermediate non-luminescent (under 365 nm) product of Cs_2_BCl_5_·H_2_O is initially synthesized which could be proved by the XRD pattern (Step-2 in Fig. 1c and Supplementary Figs. S3 and S4), and the final product Cs_2_ABCl_6_ are continuously generated accompanied the gradual releasing of A^+^ ions from ACl solid, shown in Fig. 1c (Step-3), Eqs. (7) and (8) and Video S2. As the continuous consumption of Ag^+^/Na^+^ ions, the chemical equilibriums in Eqs. (4) and (5) shift to the right side until the solids are completely depleted.

6 $$2{{{\mathrm{Cs}}}}^ + + {{{\mathrm{A}}}}^ + + {{{\mathrm{B}}}}^{3 + } + 6{{{\mathrm{Cl}}}}^ - \rightleftharpoons {{{\mathrm{Cs}}}}_2{{{\mathrm{ABCl}}}}_6 \downarrow$$

7 $$2{{{\mathrm{Cs}}}}^ + + {{{\mathrm{B}}}}^{3 + } + 5{{{\mathrm{Cl}}}}^ - + {{{\mathrm{H}}}}^ + + {{{\mathrm{OH}}}}^ - \rightleftharpoons {{{\mathrm{Cs}}}}_2{{{\mathrm{BCl}}}}_5\cdot {{{\mathrm{H}}}}_2{{{\mathrm{O}}}} \downarrow$$

8 $${{{\mathrm{Cs}}}}_2{{{\mathrm{BCl}}}}_5\cdot {{{\mathrm{H}}}}_2{{{\mathrm{O}}}} + {{{\mathrm{A}}}}^ + + {{{\mathrm{Cl}}}}^ - \rightleftharpoons {{{\mathrm{Cs}}}}_2{{{\mathrm{ABCl}}}}_6 + {{{\mathrm{H}}}}^ + + {{{\mathrm{OH}}}}^ -$$

The gradually enhancement of luminescence intensity under 365 nm UV irradiation is obtained during the stirring process (Fig. 1e, f), suggesting the phase transition from non-luminescent (under 365 nm) intermediate Cs_2_BCl_5_·H_2_O to bright Cs_2_ABCl_6_. During the actual synthesis processes, the UV sources are not recommended due to the reducibility of Ag^+^ ions, producing some purplish-black Ag solids on the surface of AgCl therefore hinders the reaction, shown in Fig. 1g (left). Notably, the products cannot be obtained without any solvents, suggesting the process from raw materials to products must goes through the “solids (raw materials)-free ions-solids (products)” rather than a direct “solid-solid” process. Therefore, the whole growth processes of the proposed HAAPP strategy still could be depicted by the LaMer model as reported by previous references (Fig. 1d)^28,29^. However, different from the conventional “dissolution in polar solvent - crystallization in poor solvent”, in our proposed HAAPP strategy, three stages (dissolution of raw materials-nucleation-growth) exist simultaneously, making it difficult to distinguish each stage separately but endowing the HAAPP strategy an attractive feature of “raw materials do not need to be pre-dissolved”. Intriguingly, the HAAPP synthesis is a process where the raw material is gradually dissolving and crystallization in a small volume of HCl environment in a short time, which makes it possible to prepare lead-free double perovskite phosphors in large quantities. Figure 1h, i shows an example of a mass preparation of Cs_2_Na_0.9_Ag_0.1_In_0.95_Bi_0.05_Cl_6_ (~6 g) with uniform luminescence characteristics.

### Spectroscopic and mechanism investigation

A series of lead-free double perovskite samples such as Cs_2_Na_1-*x*_Ag_*x*_InCl_6_ and Cs_2_Na_0.9_Ag_0.1_In_1-*y*_Bi_*y*_Cl_6_ were synthesized by using our HAAPP strategy. XRD patterns shown in Fig. 2a confirmed the pure double perovskite structures and reliable crystallization effect. The intensities of the peaks at ~14.7°, ~28.2° and 29.5° gradually decrease with the increase of Ag, proving the alloying process of Na and Ag^10^. The actual ratios of Na and Ag have been confirmed by inductively coupled plasma (ICP), shown as Supplementary Table S1, which are in line with our feeding ratios. The products exhibit weak emission when *x* = 0 or 0.8–1.0 due to the dark STE caused by a strong inversion-symmetry-induced parity-forbidden transition (Supplementary Fig. S5)^10,12,30^. The brightest luminescence was achieved by alloying 10% of Ag to break the strong inversion symmetry of [NaCl_6_] octahedrons^31,32^. The successful alloying effect was further confirmed by the enhanced STE emission intensity, broadened photoluminescence excitation (PLE) band, varied lifetime and band gap (Supplementary Figs. S5 and S6). Similarly, Cs_2_Na_0.9_Ag_0.1_In_1-*y*_Bi_*y*_Cl_6_ samples were also prepared by changing the feeding ratios of InCl_3_ and BiCl_3_. With the increase of larger sized Bi^3+^, the XRD peaks shift to the smaller degrees, with the observed phase transition process at *y* = 0.4–0.7 (Fig. 2b). Raman shifts can be also observed during the In/Bi alloying process^33–35^, shown in Supplementary Fig. S7. Meanwhile, the red-shifted PL band is observed with the increase of Bi^3+^ content (Fig. 2e and Supplementary Fig. S8), which is induced by the phase transition from direct band gap of Cs_2_NaInCl_6_ to indirect band gap of Cs_2_NaBiCl_6_^36^. With 5% Bi^3+^ alloying, the brightest emission with PLQY of (98.3 ± 3.8)% is achieved, and the central emission wavelength shifts from ~586 to ~600 nm (Fig. 2e). Meanwhile, another excitation band at ~340 nm is introduced (Fig. 2e and Supplementary Fig. S9), which should be assigned to the contributions of Bi^3+^ orbitals in the band edges (s-p transition)^7,11,17,26,36–40^, endowing their promising application value in commercial UV chip-based LEDs^24^. Scanning electron microscope (SEM) images shown in Fig. 2c and Supplementary Fig. S10 exhibit the microcrystals morphology of the Cs_2_Na_0.9_Ag_0.1_In_0.95_Bi_0.05_Cl_6_ products with mainly 0.4–1.2 μm of size distribution (Supplementary Fig. S11). The contents of elements obtained from energy dispersive spectrometer (EDS) (Supplementary Fig. S12) and ICP profiles (Supplementary Table S2) agree with our feeding ratios. Uniform distribution of all elements is also confirmed by line scanning profiles (Fig. 2d and Supplementary Fig. S13). Furthermore, no obvious shift of emission peak is found from the microsecond transient emission spectra (Fig. 2f and Supplementary Fig. S14). All of these results greatly demonstrate the pure phase and uniform luminescence of products synthesized by the HAAPP strategy.

In addition to directly mixing all of raw materials in HCl solution, the prepared products can be used as the new raw materials for secondary reaction. For example, we have added AgCl solids into the prepared Cs_2_NaInCl_6_ (in HCl solution). A secondary product Cs_2_(Na/Ag)InCl_6_ with bright STE luminescence can be obtained after stirring for several minutes, shown in Supplementary Fig. S15. No patterns of AgCl can be observed in XRD pattern, indicating a complete alloying effect of Na and Ag, and the PL spectrum exhibits broad STE emission with central wavelength of ~588 nm, which is similar to the PL behavior of directly synthesized Cs_2_(Na/Ag)InCl_6_. Furthermore, the alloyed products Cs_2_(Na/Ag)InCl_6_ can be also obtained by mixing Cs_2_NaInCl_6_ and Cs_2_AgInCl_6_ in HCl solution, shown in Supplementary Fig. S16. The successful alloying effect in secondary reactions further demonstrate the products could achieve uniform distribution of all ions through Eqs. (1–6) in HCl solution. The HAAPP strategy was conducted repeatedly for Cs_2_Na_0.9_Ag_0.1_In_0.95_Bi_0.05_Cl_6_ in 5 days to further confirm the repeatability. The XRD patterns and PL spectra of them exhibit consistent diffraction and emission behaviors, respectively, shown in Supplementary Fig. S17. Based on the ICP results, the ratio of Ag/(Ag + Na) was calculated to be 0.11 ± 0.03, which is close to the original feeding ratio (0.1), shown in Supplementary Table S3. Then, we can conclude that the products synthesized by our HAAPP strategy possess excellent uniformity and repeatability.

Next, to gain deep insights into the effect of concentrated HCl on the HAAPP strategy, we investigated the influence of HCl concentration on product yield (purple dot line) and PL intensity (orange dot line), which are shown in Fig. 2g, respectively. Firstly, the product yield gradually declines with the decreased concentration of HCl and sharply drops to ~12% at 5 wt% of HCl concentration. It is noted that the Cs_2_Na_0.9_Ag_0.1_In_0.95_Bi_0.05_Cl_6_ product can be greatly dissolved in diluted HCl compared to concentrated HCl (Supplementary Fig. S18), which is reasonable since the product prefers to exist in diluted HCl in form of free ions instead of precipitation, explaining the reason of low product yield in diluted HCl. The as-mentioned results show that a large amount of Cl^−^ in concentrated HCl strongly pushes Eq. (6) to the right side, providing a poor dissolution environment so that more products precipitate out, inducing a remarkable product yield of ~90% (Fig. 2g, Supplementary Figs. S5d and S8d); Secondly, the emission intensities of products gradually decrease with the dilution of HCl solution (Fig. 2g), indicating a worse passivation effect of the surface halogen vacancies compared to the concentrated HCl. Meanwhile, similar experiments by using other solvents (such as CH_3_COOH, CH_3_OH and NH_3_·H_2_O) were also conducted (Supplementary Fig. S19). Compared with concentrated HCl, the products synthesized in other solvents exhibit extremely weak emissions, suggesting Cl^−^-rich environment is preferred for preparing chlorinated double perovskites, which can effectively passivate the Cl^−^ vacancies on the surface to suppress the non-radiative transitions thus greatly enhance the luminescence performance. Thirdly, as shown in Supplementary Fig. S20, the XRD results show that hybrid structure phases appear with the dilution of HCl, suggesting the crystallization trend of free ions could be controlled by the provided Cl^−^-rich environment, which is thought to be one of the main factors for the preparation of pure product such as Cs_2_Na_0.9_Ag_0.1_In_0.95_Bi_0.05_Cl_6_. In addition, we found that the products could be successfully synthesized with a wide range of volume of concentrated HCl solution (Supplementary Fig. S21a), indicating that the HAAPP method does not strictly limit the proportion between the sample quantity and concentrated HCl volume. Furthermore, the concentrated HCl solution can still be used for the preparation even after 5 cycle experiments (Supplementary Fig. S21b), suggesting that the HCl is not really consumed during synthesis process, but only provide a Cl^−^-rich environment with poor solubility and precise guidance for products. The products can be also obtained with the saturated NaCl and KCl solutions (Supplementary Fig. S22), which can further prove that the Cl^−^-rich environment is one of the pivotal factors for the proposed HAAPP strategy. However, the obvious impurities (such as NaCl, KCl or Cs_2_KInCl_6_) can be observed in Supplementary Fig. S22a due to the excess Na^+^ or K^+^ ions in the solution. By contrast, the excess of H^+^ ions provided by concentrated HCl solution seem to have a negligible effect on the components of products. Therefore, the concentrated HCl solution is considered as the best option for the HAAPP strategy.

### Stability performance of products

**Fig. 3:**
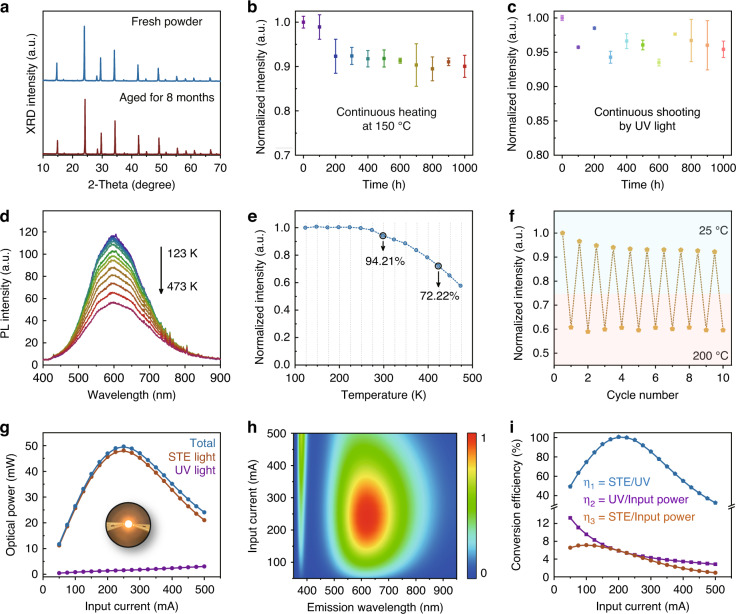
Stability performance of Cs_2_Na_0.9_Ag_0.1_In_0.95_Bi_0.05_Cl_6_ prepared by the HAAPP strategy. **a** XRD patterns of the fresh and aged Cs_2_Na_0.9_Ag_0.1_In_0.95_Bi_0.05_Cl_6_; **b** Thermal-stability of Cs_2_Na_0.9_Ag_0.1_In_0.95_Bi_0.05_Cl_6_ during continuous heating at 150 °C for 1000 h, in which the spectra were measured after cooling to the room temperature; **c** Photo-stability of Cs_2_Na_0.9_Ag_0.1_In_0.95_Bi_0.05_Cl_6_ during continuous irradiating by a 20 W, 365 nm UV lamp for 1000 h; **d** Temperature dependence of PL spectra of Cs_2_Na_0.9_Ag_0.1_In_0.95_Bi_0.05_Cl_6_; **e** Integrated PL intensity for Cs_2_Na_0.9_Ag_0.1_In_0.95_Bi_0.05_Cl_6_; **f** Response of the integrated PL intensity of Cs_2_Na_0.9_Ag_0.1_In_0.95_Bi_0.05_Cl_6_ during 10 consecutive cycles between 25 and 200 °C; The **g** optical power, **h** emission spectra and **i** conversion efficiencies of the fabricated pc-LED as a function of input current

Stability tests were conducted to examine the property of the samples synthesized through the proposed HAAPP strategy. XRD patterns for the fresh and aged Cs_2_Na_0.9_Ag_0.1_In_0.95_Bi_0.05_Cl_6_ shown in Fig. 3a exhibit unshifted peak position, suggesting there is no decomposition or secondary reaction even storage for 8 months. Under continuous heating or UV irradiation (20 W, 365 nm) for 1000 h, the PLQY of products can still maintain (90.5 ± 1.3)% and (96.8 ± 0.8)%, respectively (Fig. 3b, c), without obvious decomposition or phase transition (Supplementary Fig. S23). Temperature dependence of PL spectra recorded in Fig. 3d show that the shape and peak position do not change significantly in the range of 123–473 K, and the PL intensities remain 94.21% and 72.22% at 298 and 423 K, respectively (Fig. 3e and Supplementary Fig. S24), suggesting remarkable thermostability and photostability of the products with the HAAPP strategy. After 10 cycles of heating and cooling processes, the Cs_2_Na_0.9_Ag_0.1_In_0.95_Bi_0.05_Cl_6_ still maintains ~92.2% as intensity as the first cycle (Fig. 3f), consistent with the as-mentioned thermal-stability results (Fig. 3b), exhibiting a remarkable endurance resistance property. The remarkable resistance to the thermal- and photo-stress of products may greatly owe to the defect-free lattice guaranteed by Cl^−^-rich environment of concentrated HCl solution^10^. By taking advantage of the additional excitation band introduced by Bi^3+^ dopant, broadband emission across the entire visible region, high PLQY characteristic and excellent thermal- and photostability, the phosphor converted-LED (pc-LED) device was fabricated based on the 365 nm commercial UV chip and Cs_2_Na_0.9_Ag_0.1_In_0.95_Bi_0.05_Cl_6_ phosphor for efficient warm-white light source, shown as inset in Fig. 3g. The optical powers of STE light and total irradiation increase with input current and reach maximum values of ~48.07 and ~49.64 mW, respectively, at 250 mA. Further increasing the input current, the optical powers of STE light and total irradiation decrease due to the high temperature and heat dissipation-free measurement conditions. Figure 3h shows the emission spectra of the fabricated pc-LED device, which exhibits unshifted STE emission band, in line with the results of Fig. 3d. Intriguingly, the conversion efficiency from UV chip to STE light (η_1_) even reaches near-unity at 200 mA due to the near-unity PLQY performance of Cs_2_Na_0.9_Ag_0.1_In_0.95_Bi_0.05_Cl_6_, suggesting almost all photon energy from the UV chip is used for the STE emission (Fig. 3i). It is noted that, due to the limited optoelectronic conversion efficiency of UV chip (η_2_ in Fig. 3i, voltage in Supplementary Fig. S25), the conversion efficiency from input power to STE light (η_3_) reaches maxima of ~7.13% at 100 mA.

### Generality verification of the HAAPP strategy

**Fig. 4:**
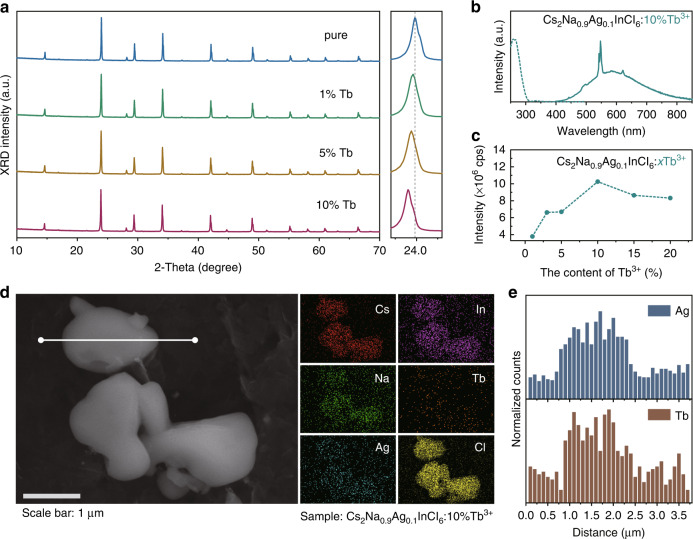
Phase, luminescence and elements distribution of prepared Cs_2_Na_0.9_Ag_0.1_InCl_6_:Tb. **a** XRD patterns of Cs_2_Na_0.9_Ag_0.1_InCl_6_: *x*Tb^3+^ (*x* = 0, 1%, 5%, 10%); **b** PLE and PL spectra of Cs_2_Na_0.9_Ag_0.1_InCl_6_: 10%Tb^3+^; **c** Integrated PL intensity as a function of the feeding contents of Tb; **d** SEM image and component elements maps of the Cs_2_Na_0.9_Ag_0.1_InCl_6_:10%Tb^3+^; **e** EDS profile of the line scanning marked in (**d**)

To verify the generality of the proposed HAAPP strategy, a series of proof-of-concept experiments have been performed. We initially attempted doping Ln^3+^ ions into Cs_2_(Na/Ag)_1_(In/Bi)_1_Cl_6_ system since Ln^3+^ ions were always doped as the luminous centers for designing advanced materials^41–45^. Taking Tb^3+^ dopant as an example, the XRD phase of the product gradually shifts to a smaller degree with the increase of Tb^3+^ (Fig. 4a), indicating the Tb^3+^ ions (*R* = 0.92 Å, CN = 6) have been successfully doped into the matrix of Cs_2_Na_0.9_Ag_0.1_InCl_6_ and substitute In^3+^ ions (*R* = 0.80 Å, CN = 6)^38^. Under 260 nm excitation, the products exhibit narrow emission peaks at ~494, 547 and 622 nm, attributing to the ^5^D_4_ → ^7^F_6_, ^5^D_4_ → ^7^F_5_ and ^5^D_4_ → ^7^F_3_ intrinsic transitions of Tb^3+^ ions, respectively (Fig. 4b). Similar to other synthesis methods^19,38,46^, the PL intensity of Tb^3+^ can be tuned by tailoring the feeding amount of Tb source (Fig. 4c). SEM image and EDS mapping shown in Fig. 4d and Supplementary Fig. S26, together with the line scanning profiles in Fig. 4e and Supplementary Fig. S27 indicate a uniform distribution of all elements. ICP measurement was further conducted for confirming the precise content of Tb^3+^ due to the sensitivity limitation of EDS instrument, shown in Supplementary Table S4. Obviously, the actual doping contents are much lower than feeding ratios, which is similar as the previous reports^17,22^, indicating a cation exchange-based doping process (Supplementary Note S3). Furthermore, a series of metal ions, such as Sb^3+^, Cr^3+^, Mn^2+^, Yb^3+^, Er^3+^, Tm^3+^, etc. have been proved successfully doped into the double perovskite microcrystals with the same HAAPP strategy, which are shown in Supplementary Figs. S28–S31 and Table S5.

**Fig. 5:**
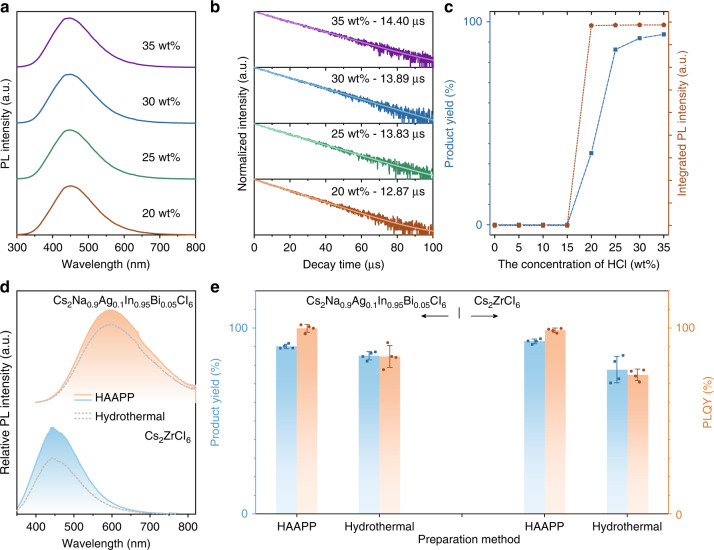
Extension of the HAAPP strategy and comparison with hydrothermal method. **a** PL spectra, **b** lifetime decay curves, **c** product yield and PL intensity of Cs_2_ZrCl_6_ prepared by the HAAPP strategy but with different concentration of HCl; Comparison of **d** PL spectra, **e** product yields and PLQY values for Cs_2_Na_0.9_Ag_0.1_In_0.95_Bi_0.05_Cl_6_ and Cs_2_ZrCl_6_ prepared by the HAAPP strategy and conventional hydrothermal method

Besides the general double perovskites Cs_2_ABCl_6_ (A = Ag/Na, B = In/Bi), a uniform vacancy-ordered halide double perovskite Cs_2_ZrCl_6_ with bright blue emission was also successfully synthesized (Fig. 5a, Supplementary Figs. S32–S36 and Video S3). Similar to the above-mentioned double perovskite Cs_2_Na_0.9_Ag_0.1_In_0.95_Bi_0.05_Cl_6_, the emission intensity of Cs_2_ZrCl_6_ gradually decrease with dilution of concentrated HCl (Fig. 5c), and the product yield declined to zero at 15 wt% of HCl concentration. Meanwhile, poorly passivated surface halogen vacancies increase the ratio of non-radiation transition, resulting in a gradual decrease in the lifetime from 14.40 to 12.87 μs while diluting HCl solution from 35 wt% to 20 wt% (Fig. 5b)^47,48^. Concentrated HCl solution is proved again as an ideal solvent for synthesis of products (Supplementary Fig. S37), and the wide range of HCl volume and the repeatability are still retained in this extended experiment (Supplementary Fig. S38). The similar thermostability measurement was also conducted for Cs_2_ZrCl_6_, which exhibited unchanged XRD patterns and ~79.1% of PLQY after continuous heating for 1000 h (Supplementary Figs. S39 and S40). Additional regulations such as red, near-infrared emissions and UVB excitation were all realized by doping Sb^3+^, Cr^3+^ and Bi^3+^ ions, respectively (Supplementary Fig. S41). Compared with the Cs_2_Na_0.9_Ag_0.1_In_0.95_Bi_0.05_Cl_6_ and Cs_2_ZrCl_6_ synthesized by conventional hydrothermal method (Fig. 5d, e and Supplementary Fig. S42), the products with similar structure by the HAAPP strategy have shown comparable crystallinity, higher product yield and brighter luminescence while achieving rapid mass production under room temperature and normal atmospheric pressure.

Furthermore, other lead-free perovskites such as standard perovskite structure represented by CsMnCl_3_ or layered perovskites represented by Cs_4_MnBi_2_Cl_12_ have been also successfully obtained by the same HAAPP strategy (Supplementary Fig. S43). In addition, a novel afterglow material of Cs_2_Na_0.9_Ag_0.1_InCl_6_:3%Mn^2+^ has been developed by the HAAPP strategy. As shown in Supplementary Fig. S44, after shot by a 4 W, 254 nm UV lamp, the product exhibited red afterglow emission, which could last for more than 150 min. More intriguingly, the HAAPP strategy can be also extended to the preparation of Br- or I-based lead-free perovskite materials by using corresponding concentrated haloid acid, such as Cs_2_AgBiBr_6_ and Cs_3_Bi_2_I_9_, etc., shown in Supplementary Fig. S45, suggesting that the HAAPP strategy can be applied not only to luminescence, but also to solar cells, photodetectors, ferroelectricity, magnetism and so on^5^. These series of proof-of-concept results prove the success of the proposed HAAPP strategy which could be comparable with conventional reported methods, and can be greatly applied to the industrialization for development of lead-free perovskite materials.

## Discussion

We have introduced a universal HAAPP strategy to achieve a series of lead-free perovskite materials. The continuous crystallization accompanies the gradual release of free ions from raw materials with low solubility, promoting the chemical equilibrium of the reversible reaction continuously to the free ions’ direction until the raw materials are exhausted. This powder-to-powder transition provides a new train of thought about the mechanism understanding of the conventional recrystallization method, that is, complete dissolution of the raw materials seems not strictly necessary. As the only used regent, the effects of concentrated HCl solution with Cl^−^-rich environment are concluded: Providing a liquid condition for rapid reactions; Giving a poor solubility for products thus improving the product yield; Enhancing the luminous performance of products by passivating the surface defects; Guiding the growth direction of free ions. Besides the advantages of thermal-, pressure-free, eco-friendliness, short time, low cost and high product yield for industrialization, the HAAPP strategy is also beneficial for the scientific research such as fluorescence investigation, afterglow regulation and photochromic, etc.

## Methods

### Raw materials

CsCl (99.99%), NaCl (99.99%), AgCl (99.99%), InCl_3_·4H_2_O (99.99%), BiCl_3_ (99.99%), ZrCl_4_ (99.9%), MnCl_2_·4H_2_O (99.9%), CrCl_3_ (99.9%), SbCl_3_ (99.9%) and corresponding LnCl_3_ or LnCl_3_·xH_2_O (99.9%) were directly used as the raw materials without further purification. Among them, AgCl and NaCl are recommended to be filtered through a 150-mesh sieve.

### Preparation of lead-free Cs_2_Na_1−*x*_Ag_*x*_In_1−*y*_Bi_*y*_Cl_6_ microcrystals

Taking the preparation of 1 mmol Cs_2_Na_0.9_Ag_0.1_In_0.95_Bi_0.05_Cl_6_ as an example, 2 mmol CsCl (0.3367 g), 0.9 mmol NaCl (0.0526 g), 0.1 mmol AgCl (0.0143 g), 0.95 mmol InCl_3_·4H_2_O (0.2785 g) and 0.05 mmol BiCl_3_ (0.0158 g) were weight and mixed in a centrifuge tube. 1–2 mL of concentrated HCl (35 wt%) was then added and shook for 5 min. The product was centrifuged at 5000 rpm for 10 s and washed by 6 mL ethanol for twice. The final sample was transferred to a 60 °C oven to dry for ~2 h and collected for the following measurements. It is noted that, AgCl and NaCl in powder state are recommended, otherwise additional sonication and vigorous stirring for more than 15 min are required (The specific time greatly depends on the block size). Furthermore, the ultraviolet light should be avoided in the preparation processes, because it can easily induce the reduction of AgCl, leading to the obvious purplish-black byproduct (that is Ag solid).

### Preparation of other lead-free perovskite microcrystals

Similar as the preparation profile of Cs_2_Na_0.9_Ag_0.1_In_0.95_Bi_0.05_Cl_6_, the Cs_2_ZrCl_6_, Cs_4_MnBi_2_Cl_12_ and CsMnCl_3_ can be easily prepared by adding mixed stoichiometric raw materials with a small amount of concentrated HCl (35 wt%) and shaking for ~1 min. The products were centrifuged at 5000 rpm for 10 s and washed by ethanol for twice. The final precipitates were transferred to a 60 °C oven to dry for ~2 h and collected for the following measurements.

### Preparation of Ln^3+^-doped lead-free perovskite microcrystals

Take the preparation of 1 mmol Cs_2_Na_0.9_Ag_0.1_InCl_6_:10%Tb^3+^ as an example. Tube A: 2 mmol CsCl (0.3367 g), 0.9 mmol NaCl (0.0526 g), 0.1 mmol AgCl (0.0143 g) and 1 mmol InCl_3_·4H_2_O (0.2932 g) were weight and mixed in a centrifuge tube. Tube B: 0.1 mmol TbCl_3_·6H_2_O (0.0373 g) was dissolved in 100 μL pure water, and added into 1–2 mL concentrated HCl (35 wt%). The mixed solution in Tube B was then added into the Tube A and shook for 5 min. The final products were centrifuged at 5000 rpm for 10 s and washed by ethanol for twice. The final precipitates were transferred to a 60 °C oven to dry for ~2 h and collected for the following measurements.

### LED fabrication

The pc-LED was fabricated by the Cs_2_Na_0.9_Ag_0.1_In_0.95_Bi_0.05_Cl_6_ phosphor and 365 nm UVB LED chip (3 W). The mass ratio of curing glue and phosphor is ~1:1, and the corresponding curing condition was irradiated by a 5 W, 365 nm light source for ~30 s.

### Hydrothermal preparation

The Cs_2_Na_0.9_Ag_0.1_In_0.95_Bi_0.05_Cl_6_ and Cs_2_ZrCl_6_ were also prepared by hydrothermal method for comparison. 2 mmol CsCl (0.3367 g), 0.9 mmol NaCl (0.0526 g), 0.1 mmol AgCl (0.0143 g), 0.95 mmol InCl_3_·4H_2_O (0.2785 g) and 0.05 mmol BiCl_3_ (0.0158 g) were weight and transferred into a 25 mL Teflon vessel and added 5 mL concentrated HCl (35 wt%) for Cs_2_Na_0.9_Ag_0.1_In_0.95_Bi_0.05_Cl_6_ preparation. Similarly, 2 mmol CsCl (0.3367 g) and 1 mmol ZrCl_4_ (0.2330 g) were weight and transferred into a 25 mL Teflon vessel and added 5 mL concentrated HCl (35 wt%) for Cs_2_ZrCl_6_ preparation. The vessels were transferred into the steel reactors and heated at 180 °C for 12 h. The cooling rate was set as 30 °C/h. The final products were wash by ethanol for twice and dried at 60 °C for ~2 h.

### Characterization

The XRD pattens of samples were confirmed by the Ultima X-ray diffractometer (Rigaku, Japan), with Cu Kα (λ = 1.5405 Å) as the irradiation source under 40 kV–40 mA power, and the scanning rate was set to 15 degrees per minute. The particle size and elements mapping were measured by the scanning electron microscope (SEM, JSM-7800F, Japan) equipped with an energy dispersive spectroscopy (EDS). The PL spectra, PLE spectra, PLQY, PL decay curves, persistent emission spectrum and afterglow decay curves were measured by a spectrofluorometer FLS-1000 (Edinburg, England) and further confirmed by another spectrofluorometer FS-5 (Edinburg, England). In the PL comparison measurements, the samples were accurately weighed and confined to the same sample tank, and the quartz glass was used to cover the surface to ensure consistency of the thickness and surface roughness of all samples. The measurement systems were calibrated by commercial YAG:Ce^3+^ phosphor with a standard PLQY of ~80% under 460 nm excitation. The absorption spectra were measured by a UV–Vis spectrophotometer (UV-2600, Shimadzu, Japan). The inductively coupled plasma (ICP) measurements were conducted by the Agilent ICPOES-730. Raman spectra were carried out on RMS-1000 (Edinburg, England) spectrometer with an excitation source of 532 nm laser. The digital photographs were pictured by a smartphone (Xiaomi 12S Ultra). The pc-LED device was measured in a glovebox without encapsulation. The current density-voltage responses were recorded by using a Keithley 2400 source meter with a step of 25 mA cm^−2^. The emission spectra of pc-LED were collected by using an integrating sphere (Ocean Optics, FOIS-1) coupled with a spectrophotometer (Ocean Optics, QE65 Pro). The temperature dependent-PL spectra were obtained by a spectrophotometer (Aurora 4000, GE-UV-NIR, Changchun New Industries Optoelectronics Tech. Co., Ltd) equipped with a temperature module (HCS421VXY, Instec, Shanghai Hengshang Precision Instrument Co., Ltd) for temperature control.

## Supplementary information

Supplementary information

Video S1

Video S2

Video S3

## Data Availability

The data that support these findings are available from the corresponding author upon request. Source data of all figures are provided with this paper.

## Source data

Source data
