# Supplementary material for: A universal hydrochloric acid-assistant powder-to-powder strategy for quick and mass preparation of lead-free perovskite microcrystals
Source: Light Sci Appl. 2023 Mar 20;12:75. doi: 10.1038/s41377-023-01117-2 (PMC10025261; doi:10.1038/s41377-023-01117-2)
Supplement: Supplementary file 1 — Supplementary information [file 41377_2023_1117_MOESM1_ESM.docx]

**Supplementary Information for**

**A universal hydrochloric acid-assistant powder-to-powder strategy for quick and mass preparation of lead-free perovskite microcrystals**

Huanxin Yang^1^, Xiangxiang Chen^2^, Yiyue Chu^3^, Changjiu Sun^4^, Haolin Lu^5^, Mingjian Yuan^4^, Yuhai Zhang^2^, Guankui Long^5^, Libing Zhang^3^ & Xiyan Li^1,^*

**Note S1**

**Video S1:** Mixing the raw materials together, and then adding a small amount of concentrated HCl solution. The bright luminescence of the mixture can be observed under UV irradiation. The nonuniform luminescence of the products in Video S1 was caused by the uneven mixing and stirring-free condition.

**Video S2:** Adding the non-luminescent intermediate product Cs_2_In_0.95_Bi_0.05_Cl_5_·H_2_O dispersed in concentrated HCl solution to the mortar containing the AgCl solids, and a bright emission around AgCl solids can be observed under UV irradiation, corroborating the phase transition from Cs_2_In_0.95_Bi_0.05_Cl_5_·H_2_O to Cs_2_AgIn_0.95_Bi_0.05_Cl_6_.

**Video S3:** A blue light emission can be observed immediately under UV irradiation once adding concentrated HCl solution into the centrifuge tube containing CsCl and ZrCl_4_ powders.

**Note S2**

**Double exponential decays** are fitted by equation S1 and the average lifetimes are calculated by equation S2:

|  | *I*(*t*) = *A*_1_ exp(-*t*/*τ*_1_) + *A*_2_ exp(-*t*/*τ*_2_) + *I*_0_ | (S1) |
| --- | --- | --- |
|  | *τ*_avg_ = (*A*_1_*τ*_1_^2^ + *A*_2_*τ*_2_^2^) / (*A*_1_*τ*_1_ + *A*_2_*τ*_2_) | (S2) |

**Triple exponential decays** are fitted by equation S3 and the average lifetimes are calculated by equation S4:

|  | *I*(*t*) = *A*_1_ exp(-*t*/*τ*_1_) + *A*_2_ exp(-*t*/*τ*_2_) + *A*_3_ exp(-*t*/*τ*_3_) + *I*_0_ | (S3) |
| --- | --- | --- |
|  | *τ*_avg_ = (*A*_1_*τ*_1_^2^ + *A*_2_*τ*_2_^2^ + *A*_3_*τ*_3_^2^) / (*A*_1_*τ*_1_ + *A*_2_*τ*_2_ + *A*_3_*τ*_3_) | (S4) |

The *I*(*t*) and *I*_0_­ are the emission intensities at times *t* and zero, respectively. *A*_1_, *A*_2_ and *A*_3_ are constants. The *τ*_1_, *τ*_2_ and *τ*_3_ correspond to the decay lifetimes of different proportions, respectively. The *τ*_avg_ is the average lifetime. The fitting criterions of all lifetime decay curves are *R*^2^ > 0.99.

In Table S6, the average lifetimes decrease with the increase of Ag, suggesting Na/Ag alloying process. Triple exponential fitting methods are applied in *x* = 0.3-0.6, corresponding to the critical range of phase transition from Cs_2_NaInCl_6_ to Cs_2_AgInCl_6_.

In Table S7, the lifetime decay curves are all fitted by double exponential method and show similar average lifetimes in *y* = 0-0.8. The rapid decline of average lifetime from 9.52 to 6.80 μs with increase of *y* from 0.8 to 1.0 should be attributed to the increasing dominance of the indirect bandgap of Cs_2_Na_0.9_Ag_0.1_BiCl_6_.

**Note S3**

The actual doping concentrations of Ln^3+^ ions and transition metals are much lower than the feeding ratios, which are similar to the previous reports (refs. 17, 22, 26, 39, 40, etc.). Here, taking Tb as an example, some possible reasons are concluded:

1. The products Cs_2_NaInCl_6_, Cs_2_AgInCl_6_, Cs_2_NaBiCl_6_ and Cs_2_AgBiCl_6_ can be synthesized individually by using the proposed HAAPP strategy. However, Tb-based double perovskites (Cs_2_NaTbCl_6_ or Cs_2_AgTbCl_6_) cannot be synthesized, suggesting that the In and Tb cannot be alloyed like In/Bi or Ag/Na in this strategy.
2. DFT calculation was carried out for Cs_2_NaInCl_6_ (-1.46 eV·mol^-1^) and Cs_2_NaTbCl_6_ (-0.81 eV·mol^-1^). Compared to Cs_2_NaInCl_6_, the higher formation energy of Cs_2_NaTbCl_6_ may also be one of the reasons for the low doping ratios.

**Calculation details of the formation energies**

The Vienna Ab-initio Simulation Package (VASP) software was used to accomplish the density functional theory (DFT) calculations. The projector augmented wave (PAW) method with the Perdew-Burke-Ernzerhof (PBE) functional as revised for solids (PBEsol) was selected and the plane-wave cutoff energy was set to 500 eV. Geometric structures are fully relaxed without any restrictions for symmetries until the energy and total forces were converged to 10^-5^ eV and 0.001 eV·Å^-1^, respectively. Γ-centered Monkhorst-Pack mesh of 21×21×21 k-points were employed for sampling the Brillouin zones for structural relaxations and static calculations.

The formation energies E(formation) are defined as Equation S5:

|  | E(formation) = E(perovskite) - E(CsCl) - E(NaCl) - E(BCl_3_) | (S5) |
| --- | --- | --- |

where B represents the trivalent metal cations. E(perovskite), E(CsCl), E(NaCl) and E(BCl_3_) are the total free energies of perovskites and reactants of metal chloride salts, respectively.


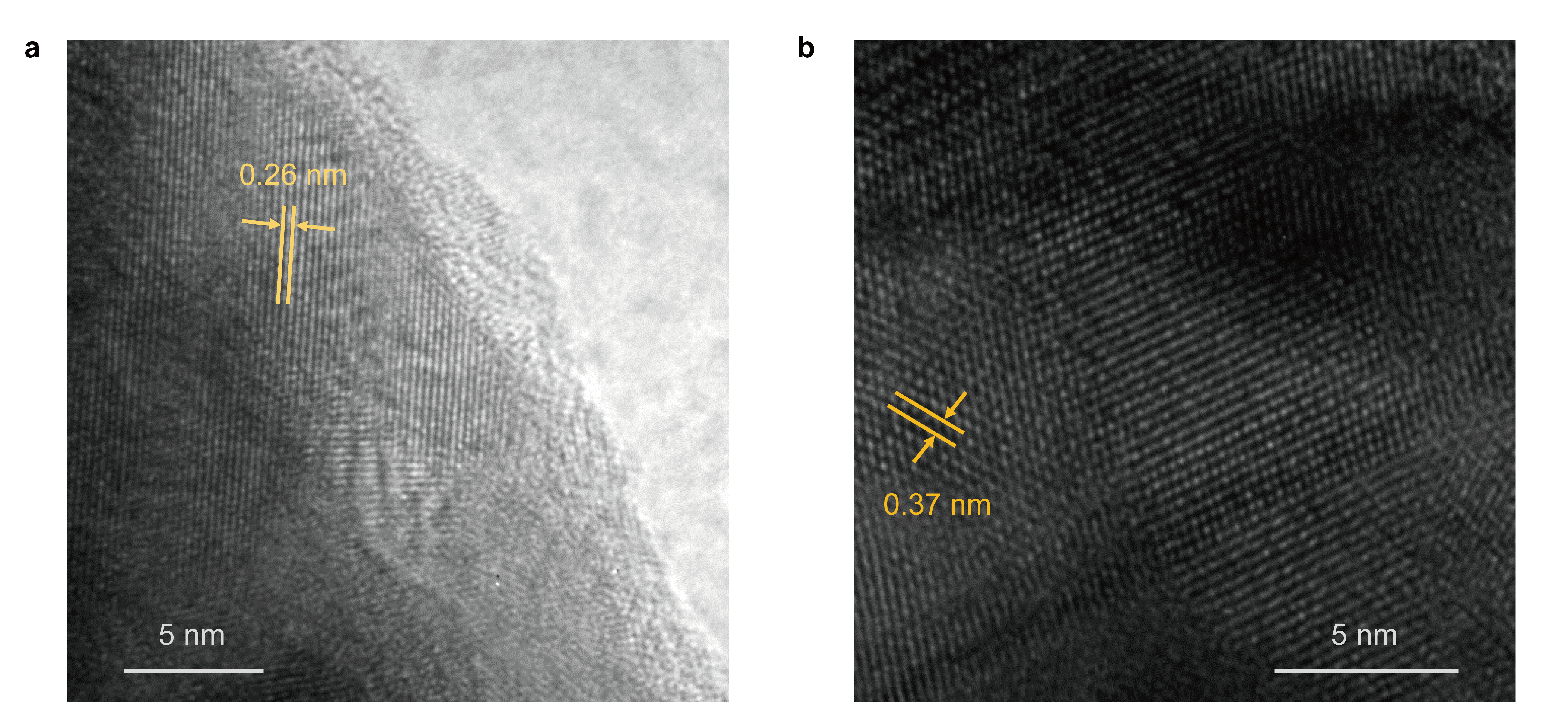


**Fig. S1. High-resolution TEM images of Cs_2_Na_0.9_Ag_0.1_In_0.95_Bi_0.05_Cl_6_ prepared by the HAAPP strategy.**

The relationship between lattice spacing and XRD peaks can be calculated according to the Braggs law, shown as equation S6:

|  | *nλ* = 2*d*·sin*θ* | (S6) |
| --- | --- | --- |

in which *nλ* represents integer multiples of wavelength, while *d* and *θ* are lattice spacing and incidence angle, respectively. The spacing values of 0.26 and 0.37 nm correspond to the diffraction peaks of ~34.1^o^ (400) and ~23.9^o^ (220), respectively.


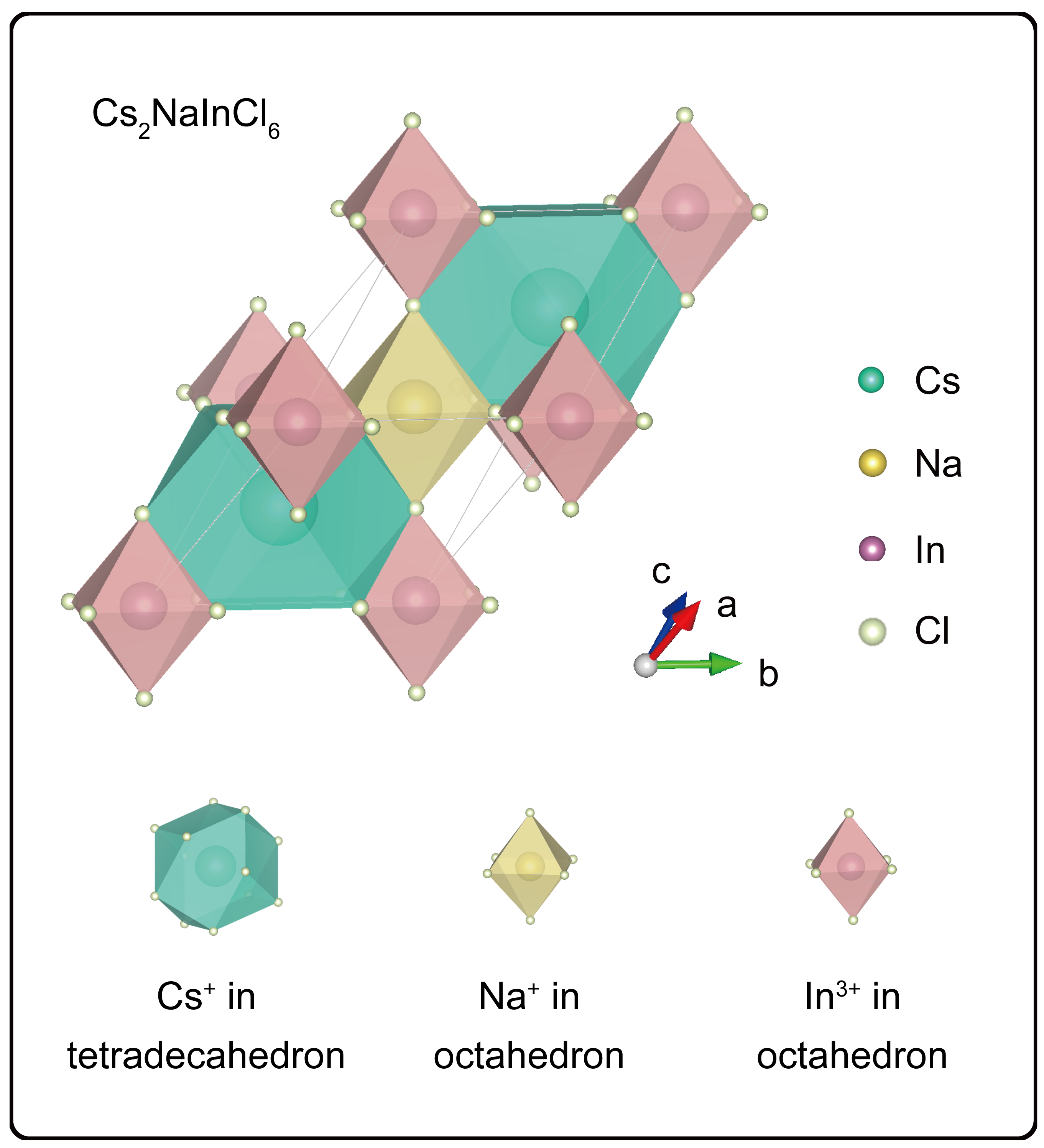


**Fig. S2. Standard crystal structure of Cs_2_NaInCl_6_.**


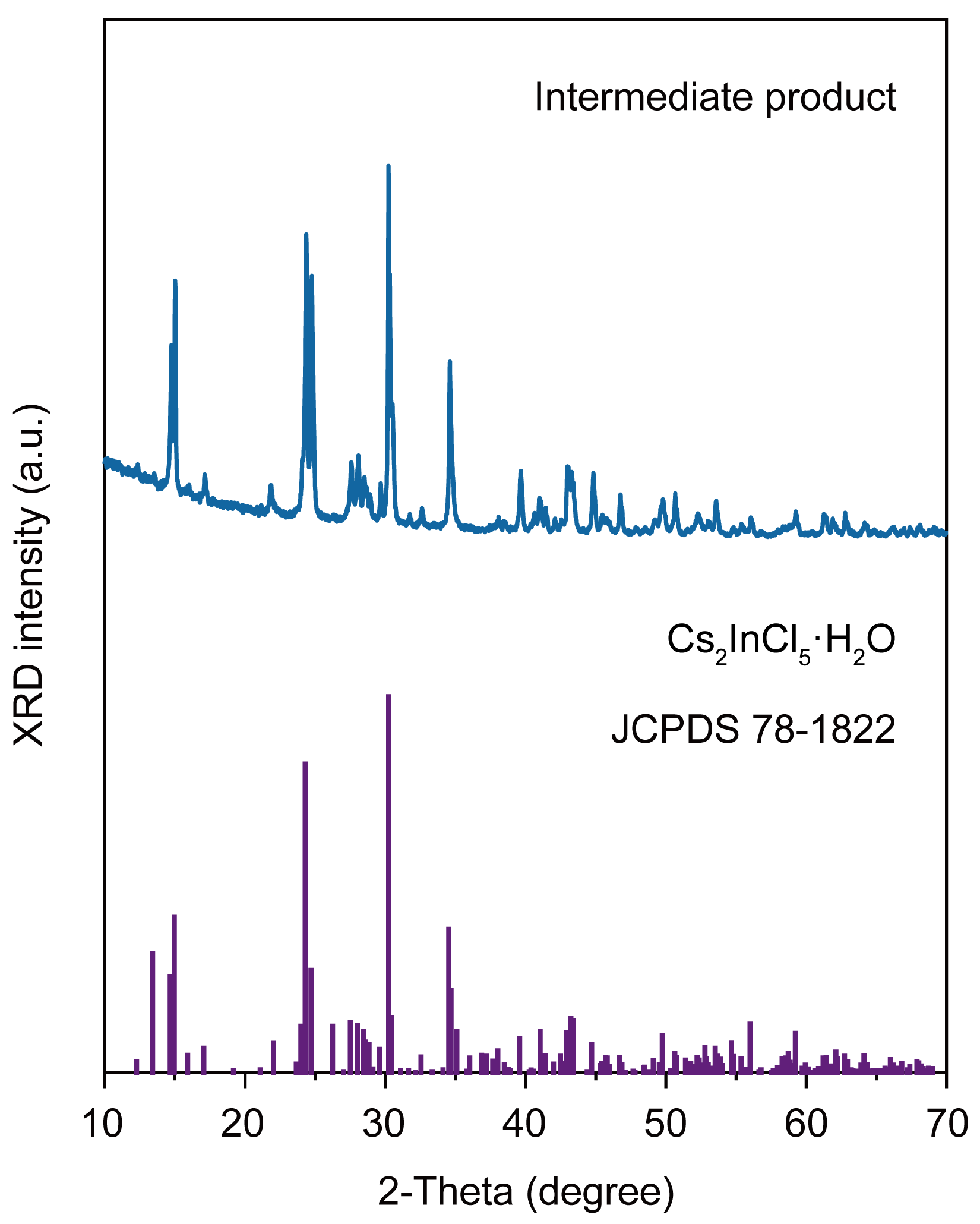


**Fig. S3. XRD pattern for the intermediate product during the HAAPP preparation.**


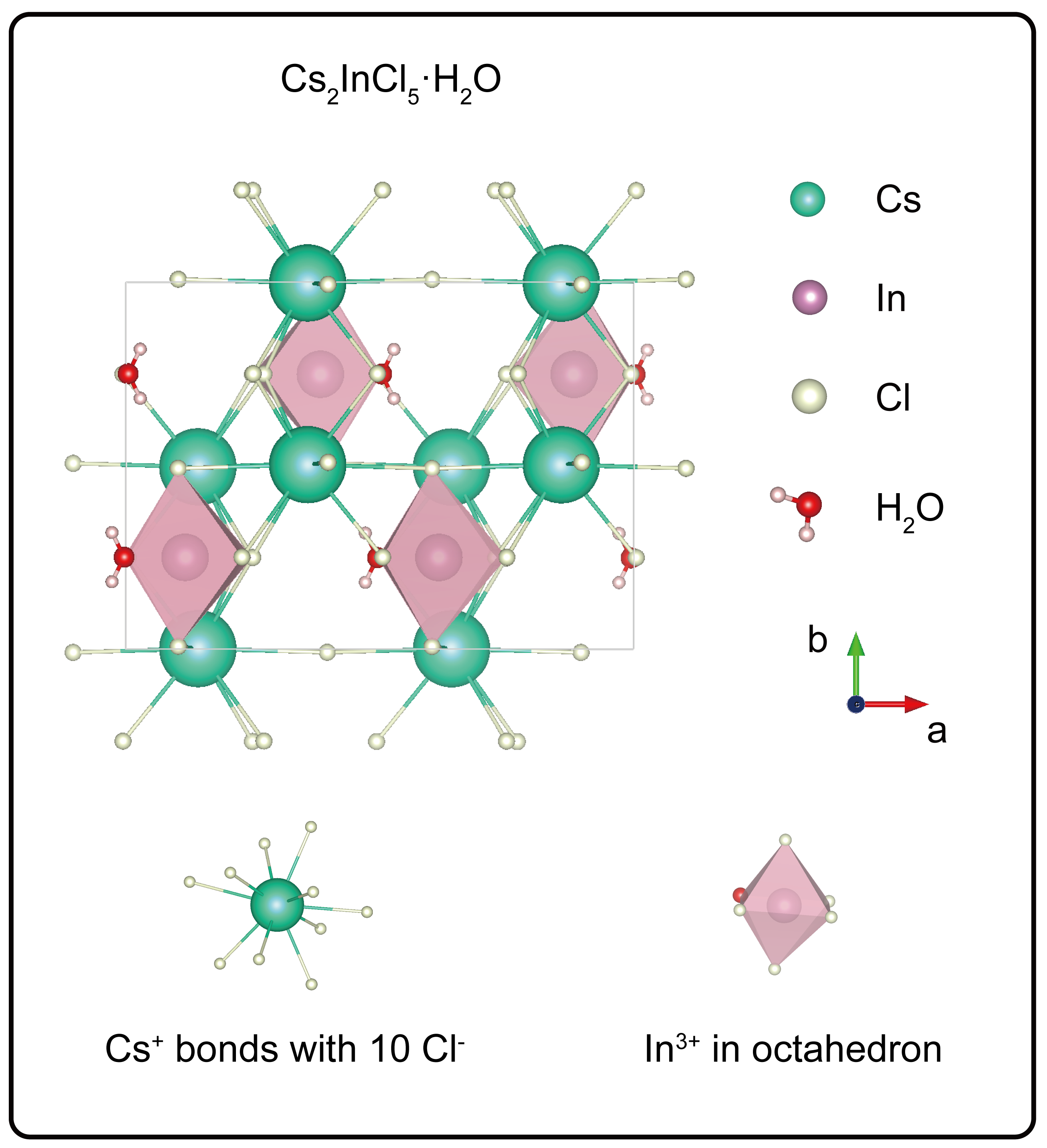


**Fig. S4. Standard crystal structure of Cs_2_InCl_5_·H_2_O.**


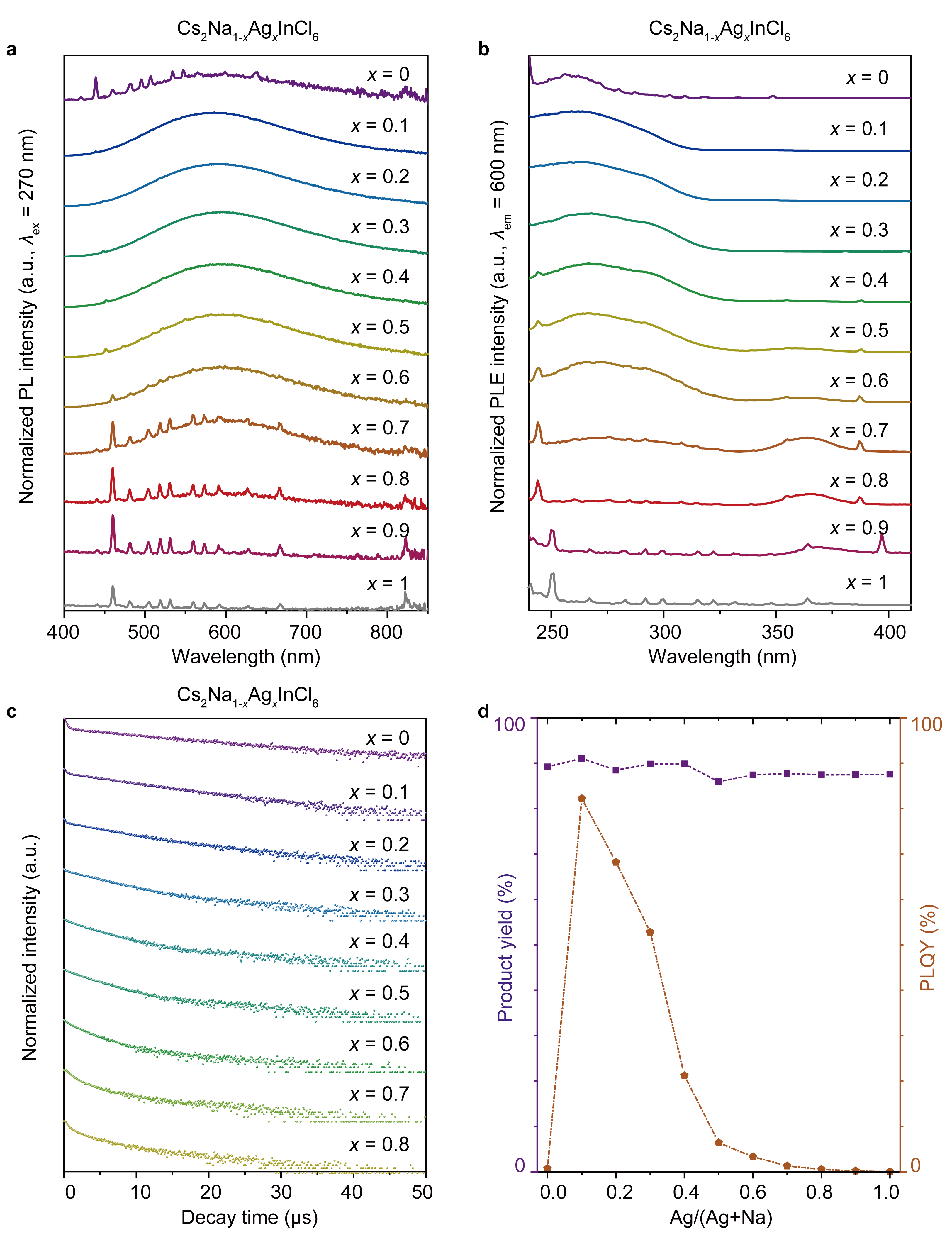


**Fig. S5. Fluorescent performance of Cs_2_Na_1-_*_x_*Ag*_x_*InCl_6_. (a)** PL and **(b)** PLE spectra; **(c)** Lifetime decay curves; **(d)** Product yields and PLQY values as a function of Ag content.

From the Figs. S5a and S5b, no obvious PL or PLE signal can be detected for *x* = 1 and 0.9 due to the dark STE caused by strong parity-forbidden effect (ref. 10 in text). Therefore, the lifetime decay curves were measured only for *x* = 0-0.8. The fitting results for lifetime decay curves are shown in Table S6, and the fitting criterions are described in Note S2.


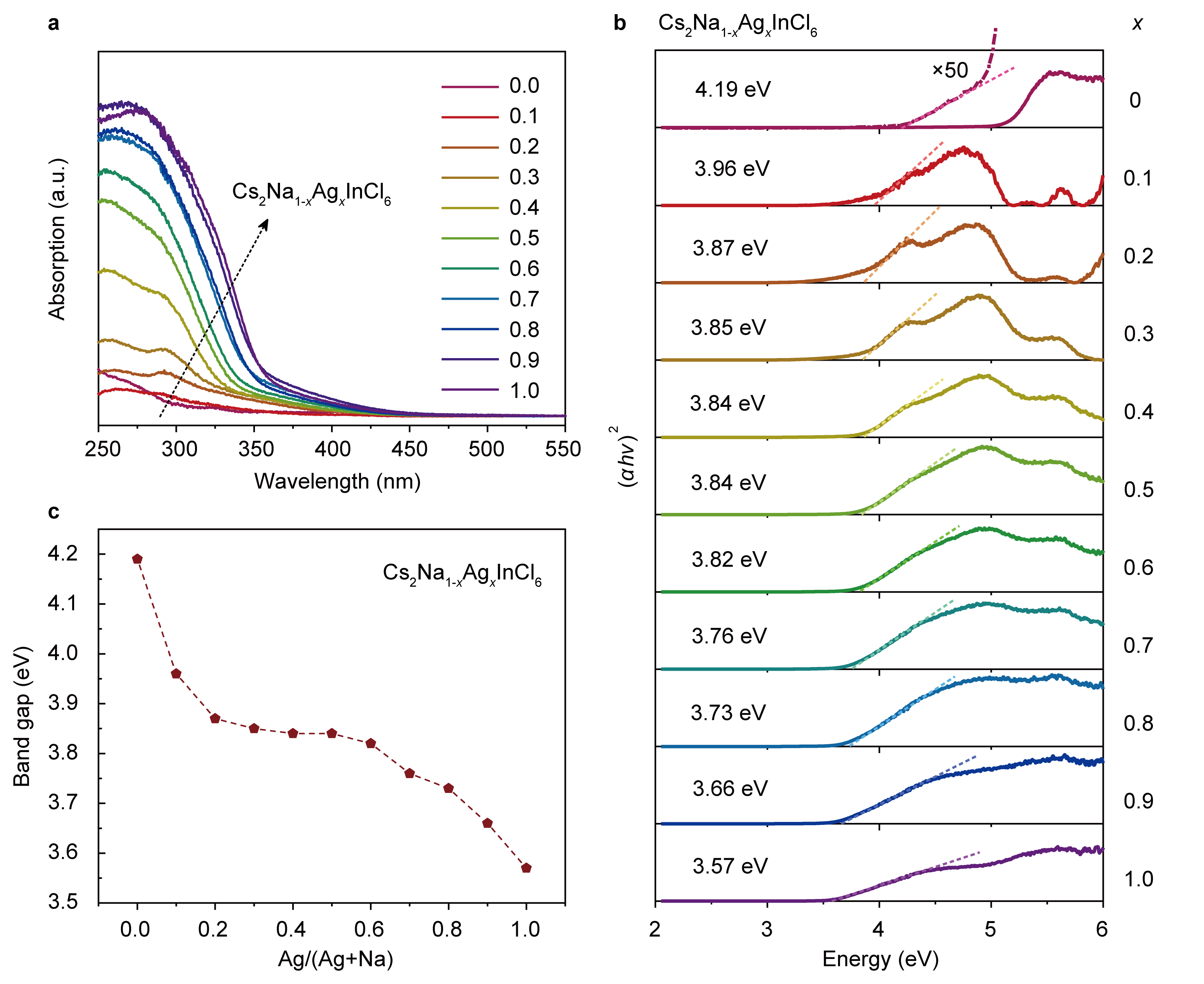


**Fig. S6. Absorption spectra and fitted band gaps of Cs_2_Na_1-_*_x_*Ag*_x_*InCl_6_. (a)** Absorption spectra; **(b, c)** Fitting results of band gaps.


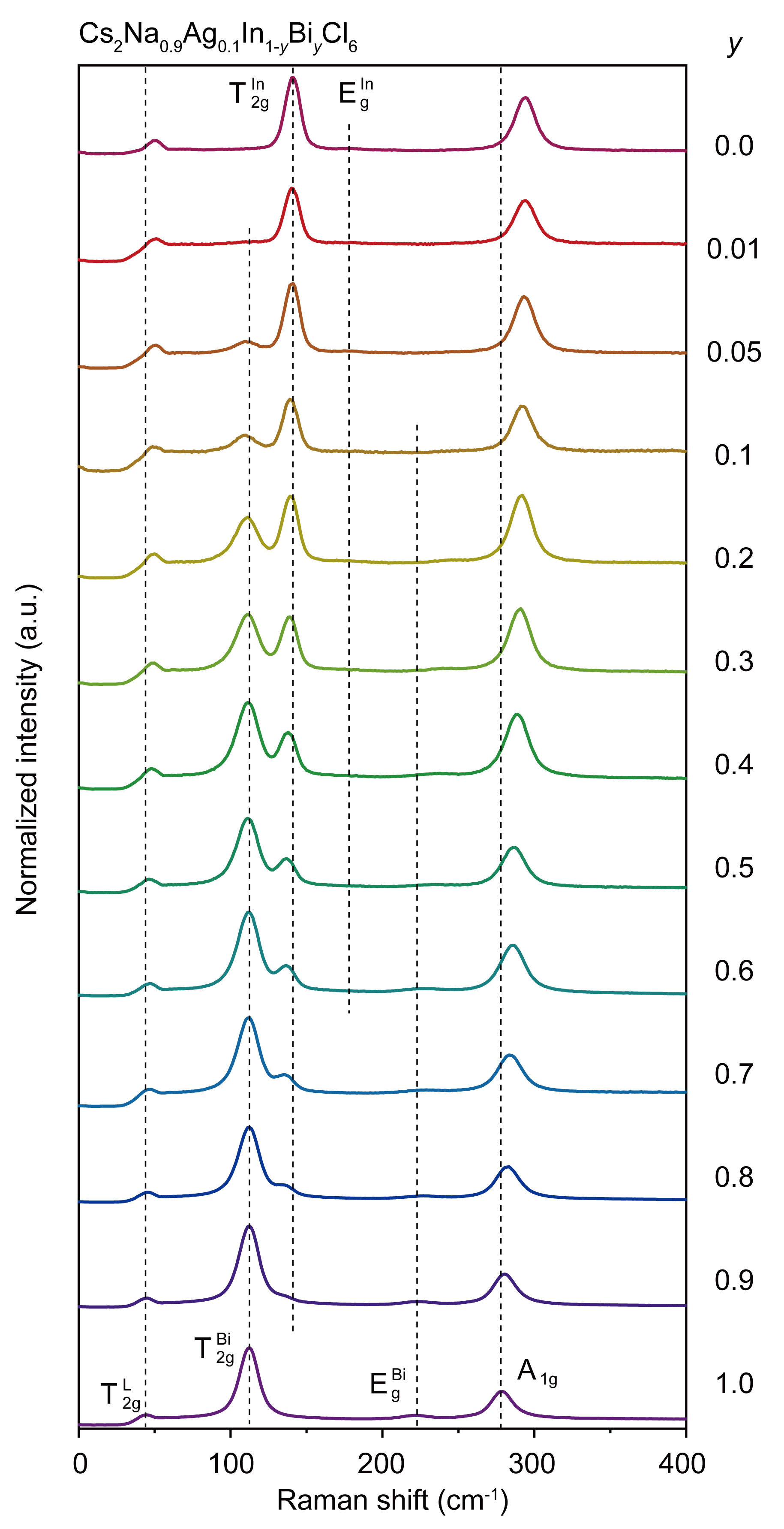


**Fig. S7. Raman spectra of Cs_2_Na_0.9_Ag_0.1_In_1-_*_y_*Bi*_y_*Cl_6_ products prepared by the HAAPP strategy.**


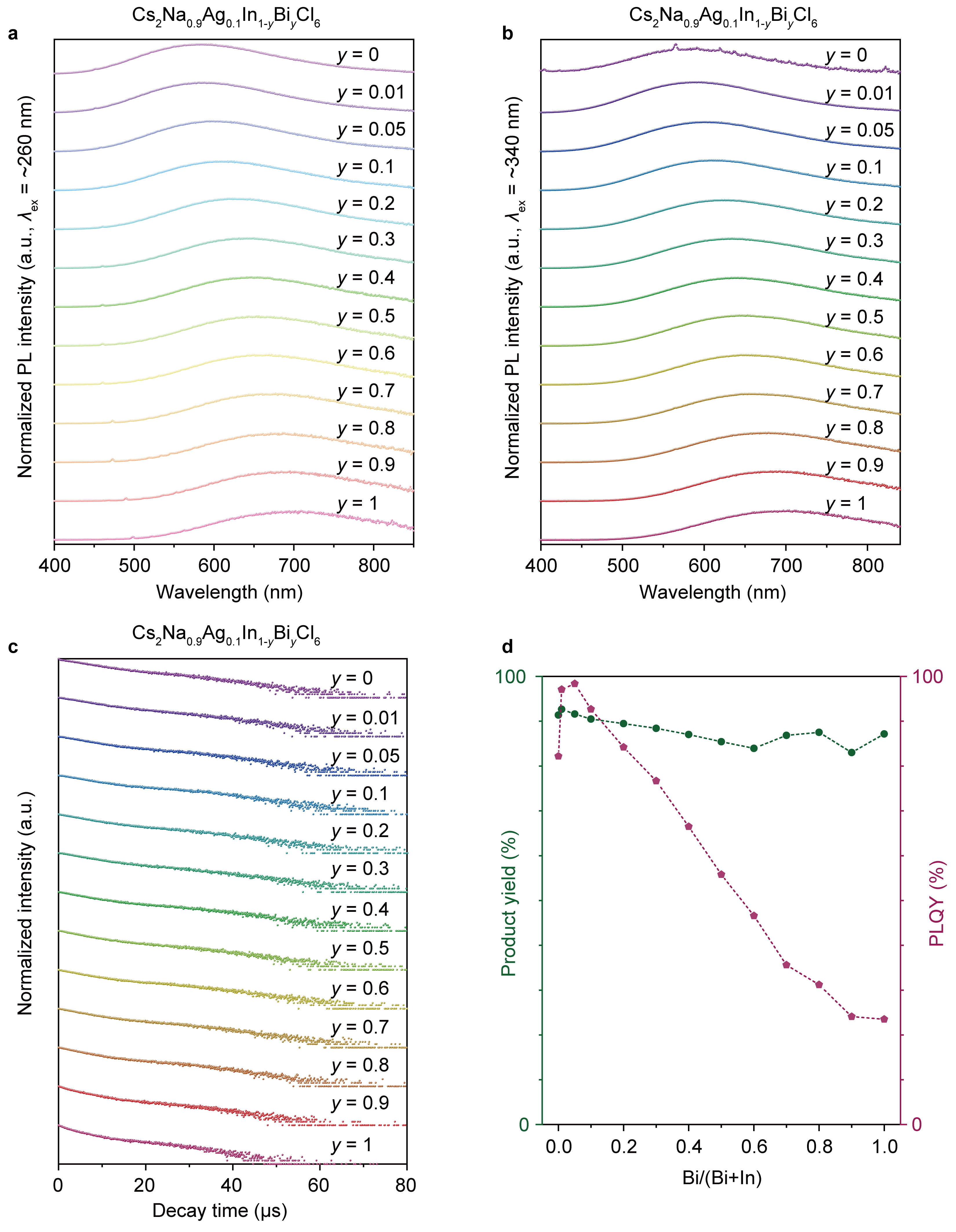


**Fig. S8. Fluorescent performance of Cs_2_Na_0.9_Ag_0.1_In_1-_*_y_*Bi*_y_*Cl_6_.** PL spectra excited at **(a) ~**260 and **(b)** ~340 nm, respectively; **(c)** Lifetime decay curves; **(d)** Product yields and PLQY values as a function of Bi content.

The fitting results for Fig. S8c are shown in Supplementary Table S7, and the fitting criterions are described in Supplementary Note S2.


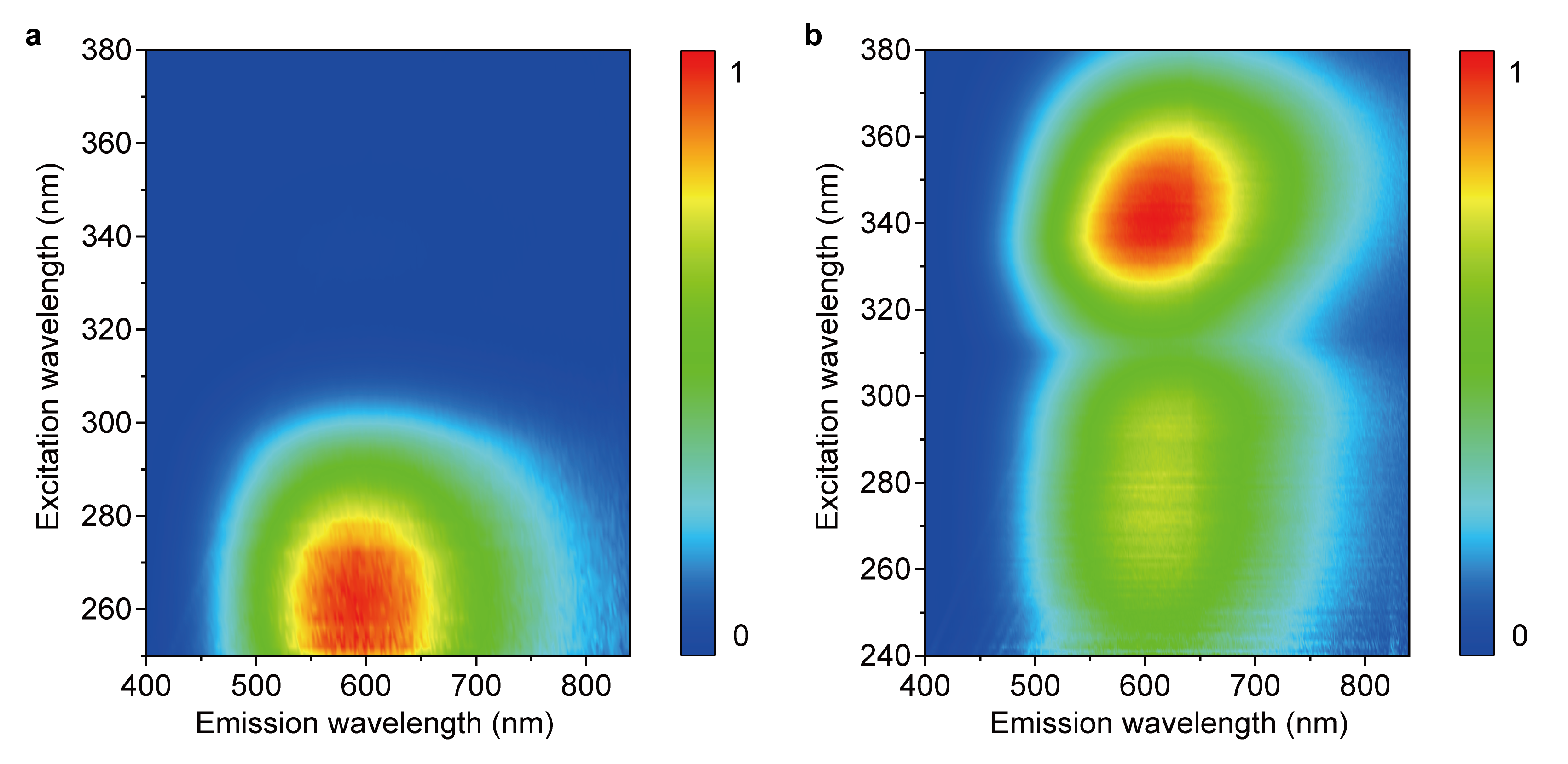


**Fig. S9. PLE spectra of (a)** Cs_2_Na_0.9_Ag_0.1_InCl_6_; **(b)** Cs_2_Na_0.9_Ag_0.1_In_0.95_Bi_0.05_Cl_6_.


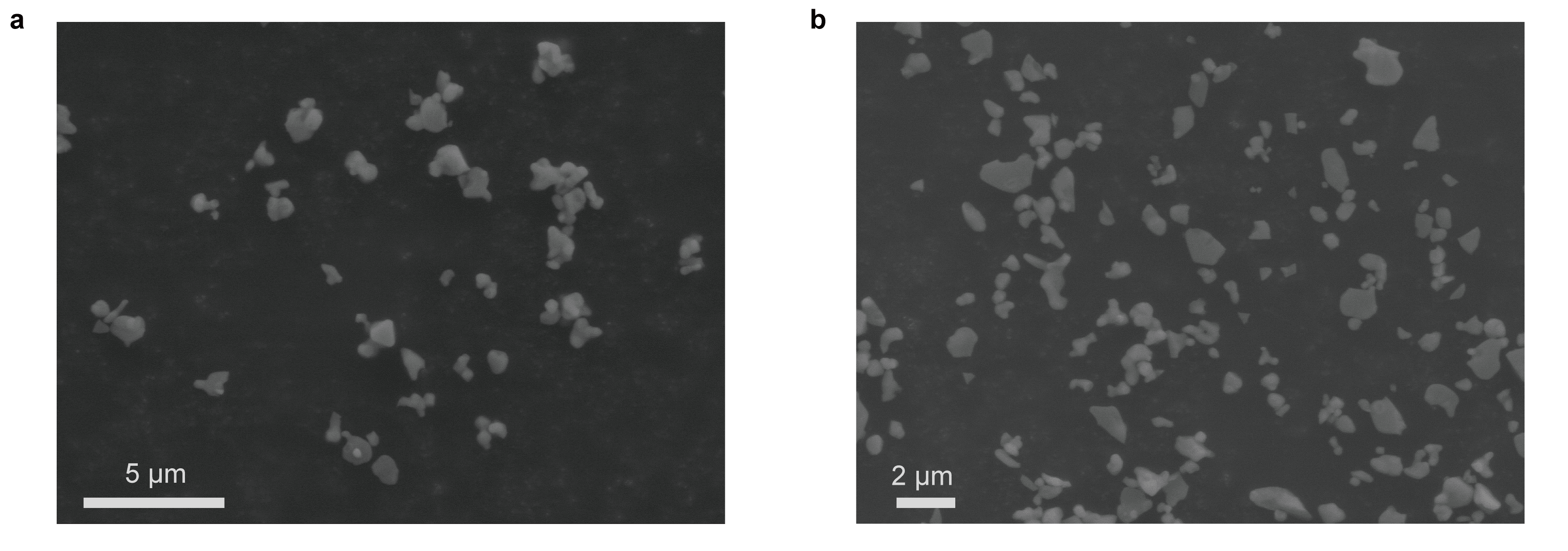


**Fig. S10. SEM images of Cs_2_Na_0.9_Ag_0.1_In_0.95_Bi_0.05_Cl_6_ prepared by the HAAPP strategy at low magnification.**


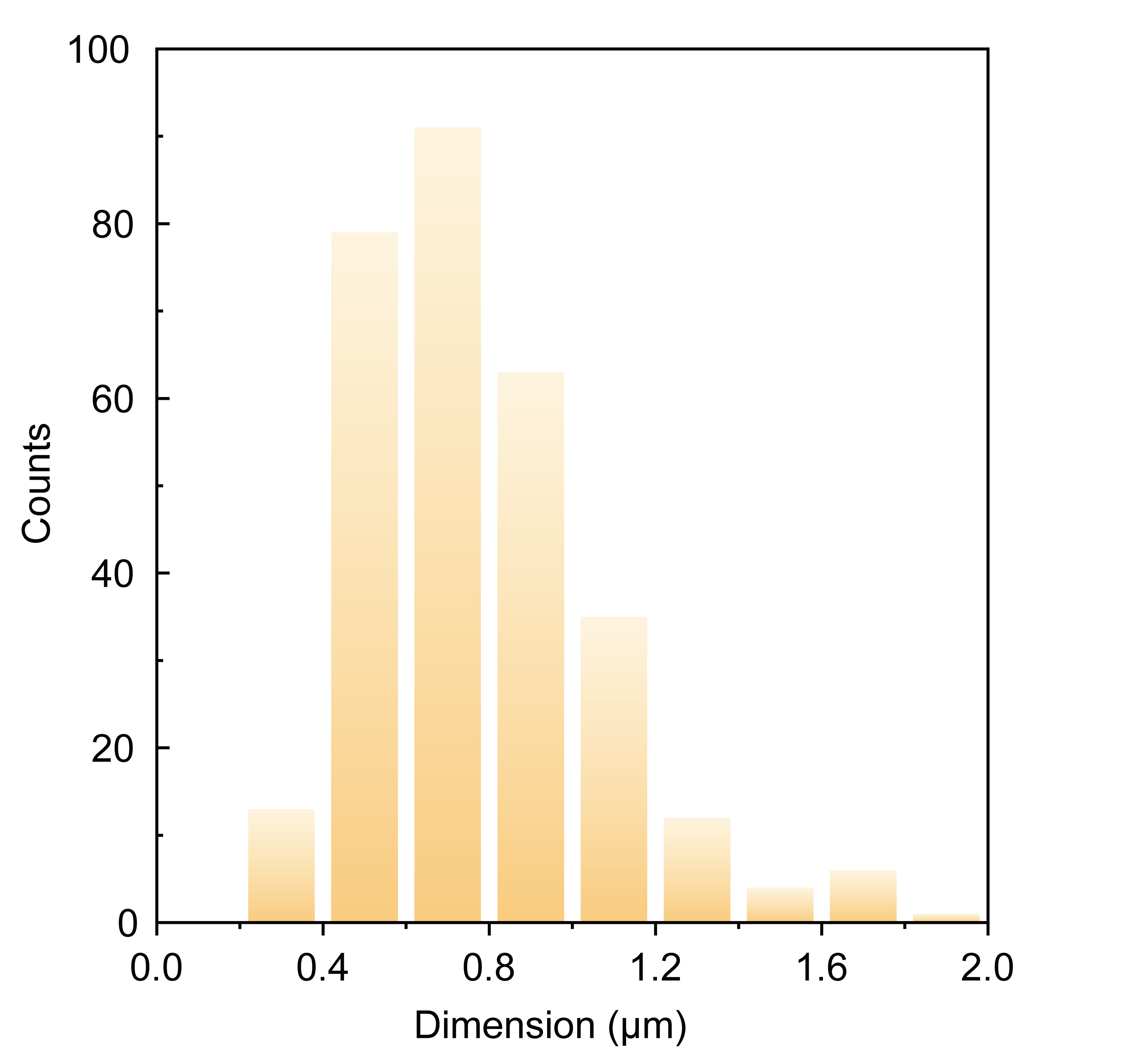


**Fig. S11. Size distribution of Cs_2_Na_0.9_Ag_0.1_In_0.95_Bi_0.05_Cl_6_ products prepared by the HAAPP strategy.**


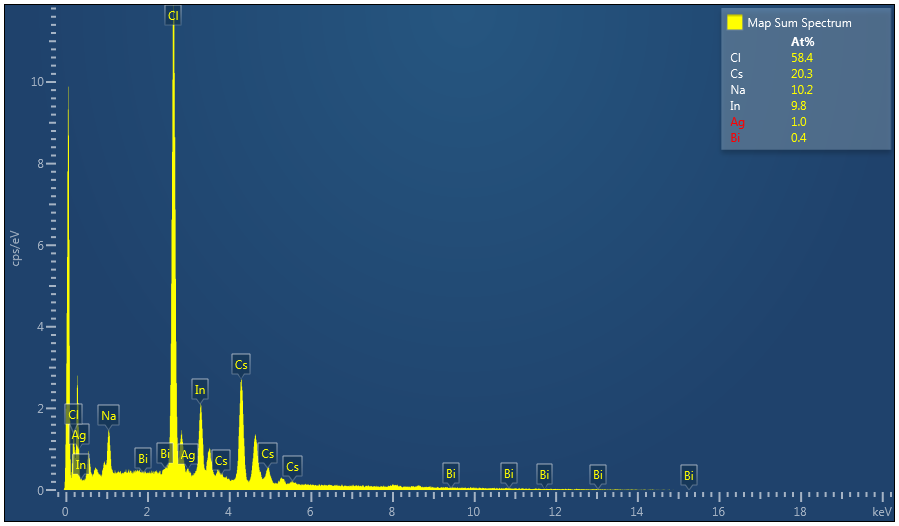


**Fig. S12. EDS spectrum of Cs_2_Na_0.9_Ag_0.1_In_0.95_Bi_0.05_Cl_6_ for Fig. 2c.**

**
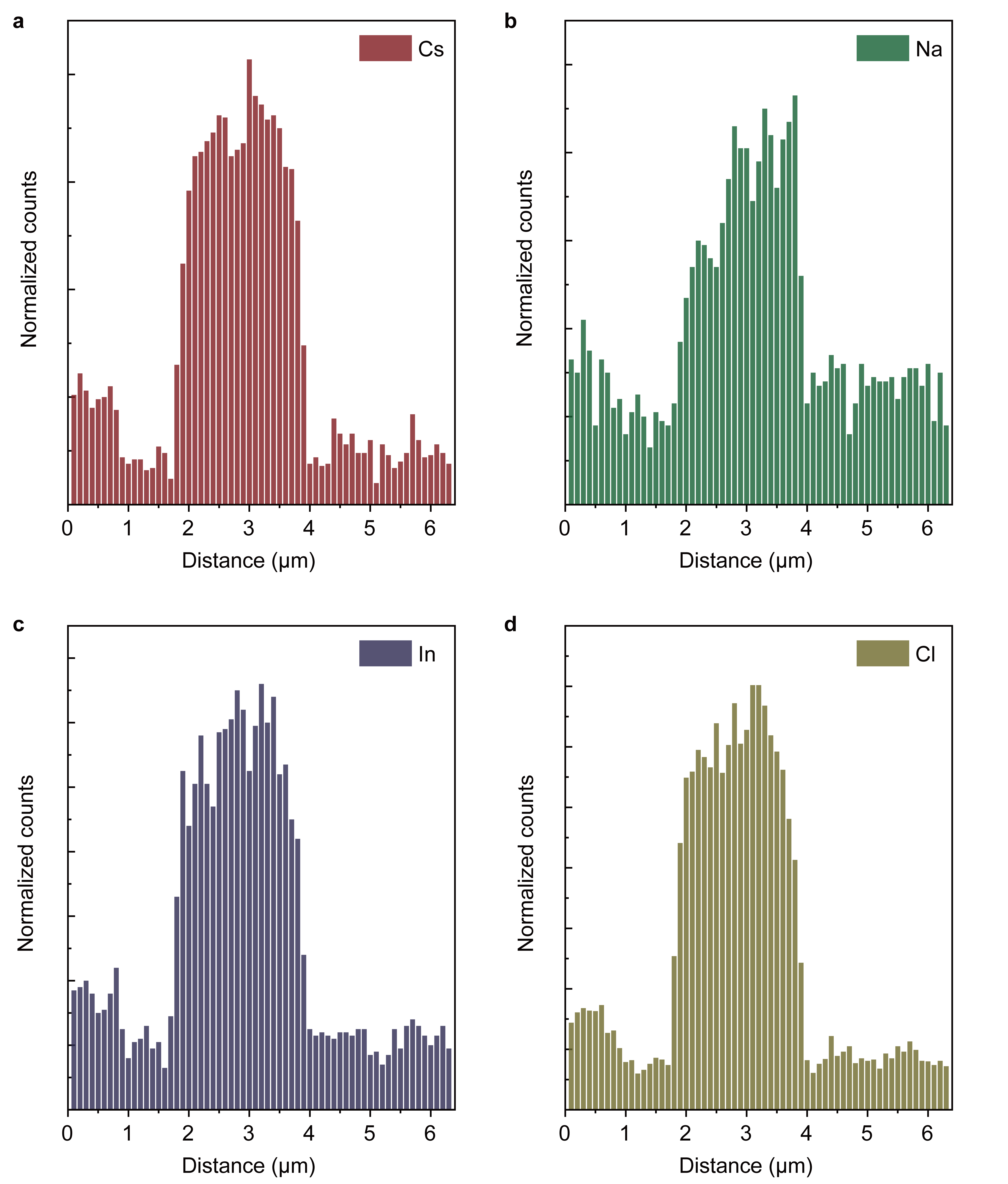
**

**Fig. S13. EDS profile of the line scanning marked in Fig. 2c.** For **(a)** Cs, **(b)** Na, **(c)** In and **(d)** Cl elements.


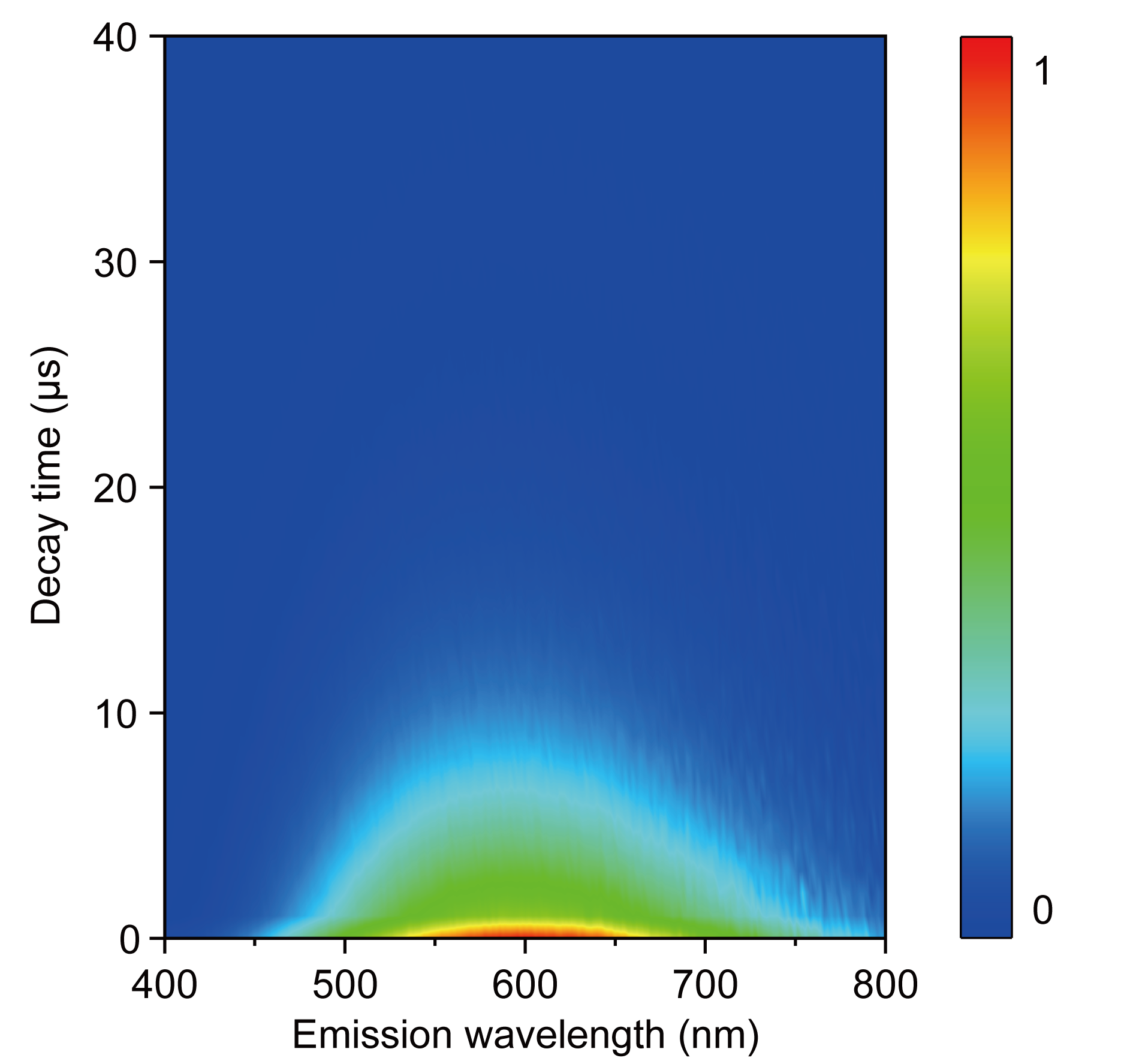


**Fig. S14. Microsecond transient emission spectra of** **Cs_2_Na_0.9_Ag_0.1_In_0.95_Bi_0.05_Cl_6_ excited at 260 nm.**

**
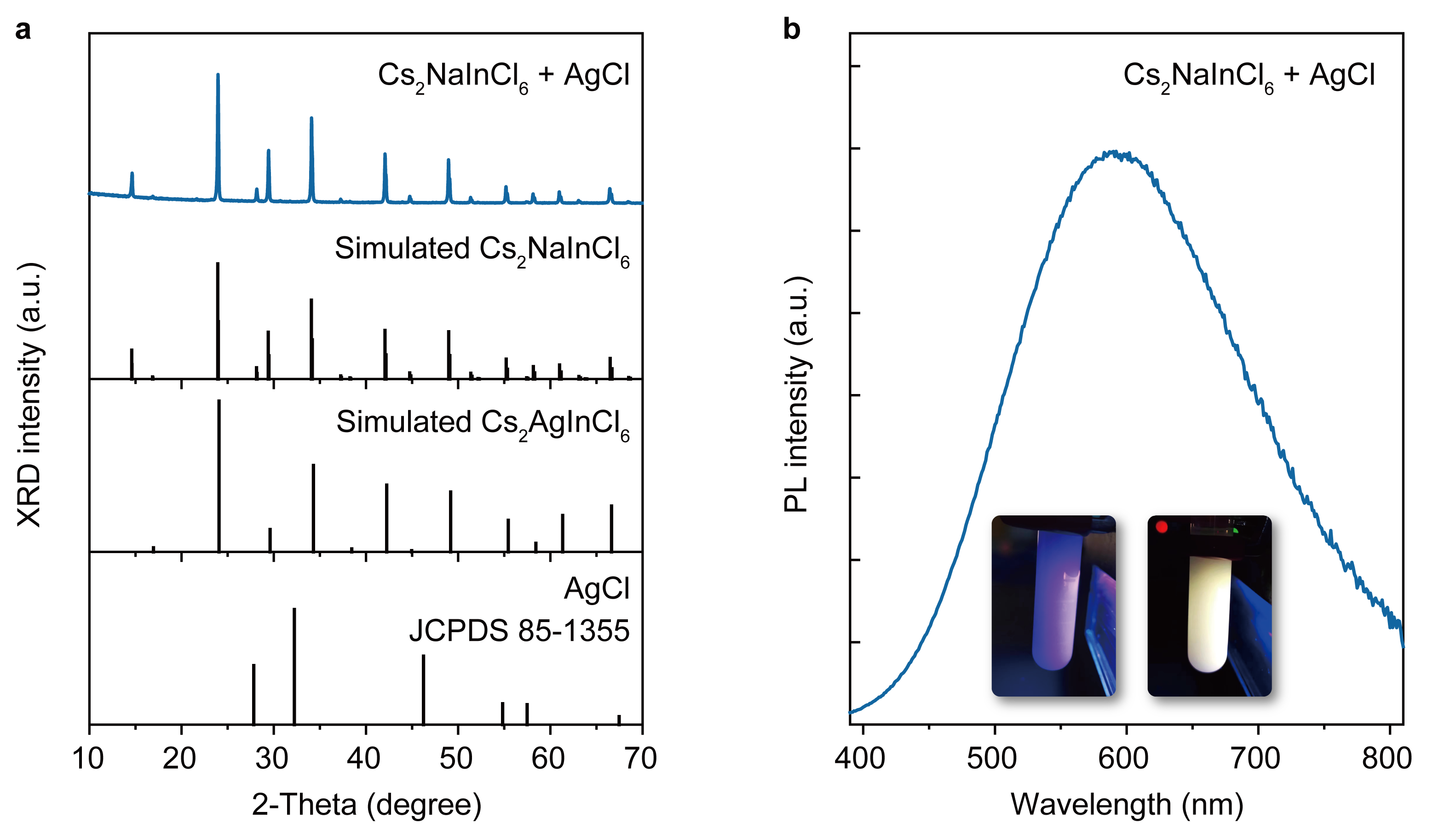
**

**Fig. S15. XRD pattern and PL spectrum of products prepared by mixing Cs_2_NaInCl_6_ and AgCl.** The insets in Fig. S15b are digital pictures of samples (under a 4 W, 254 nm lamp) before (left) and after (right) alloying reaction.

**
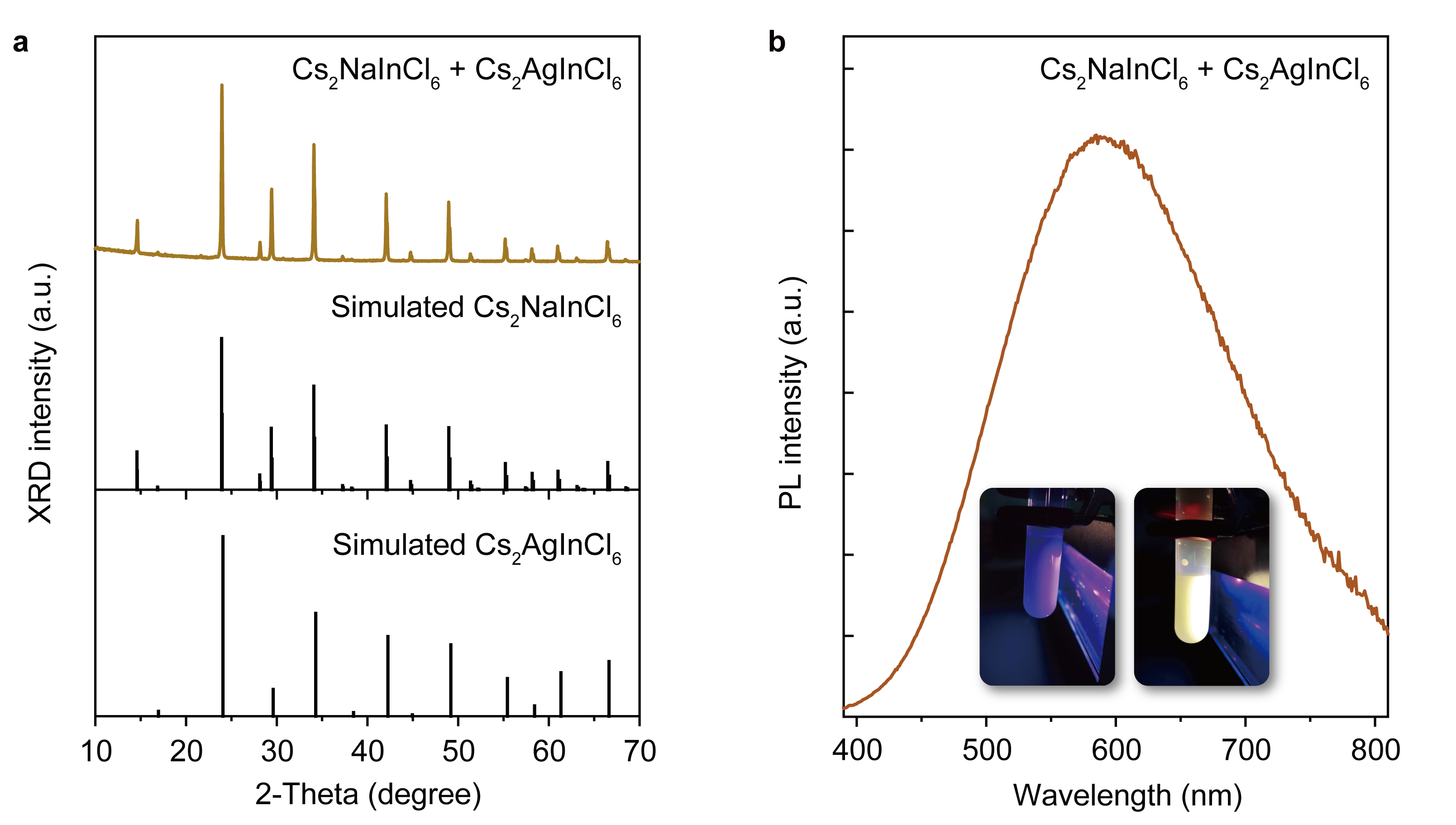
**

**Fig. S16. XRD pattern and PL spectrum of products prepared by mixing Cs_2_NaInCl_6_ and Cs_2_AgInCl_6_.** The insets in Fig. S16b are digital pictures of samples (under a 4 W, 254 nm lamp) before (left) and after (right) alloying reaction.

**
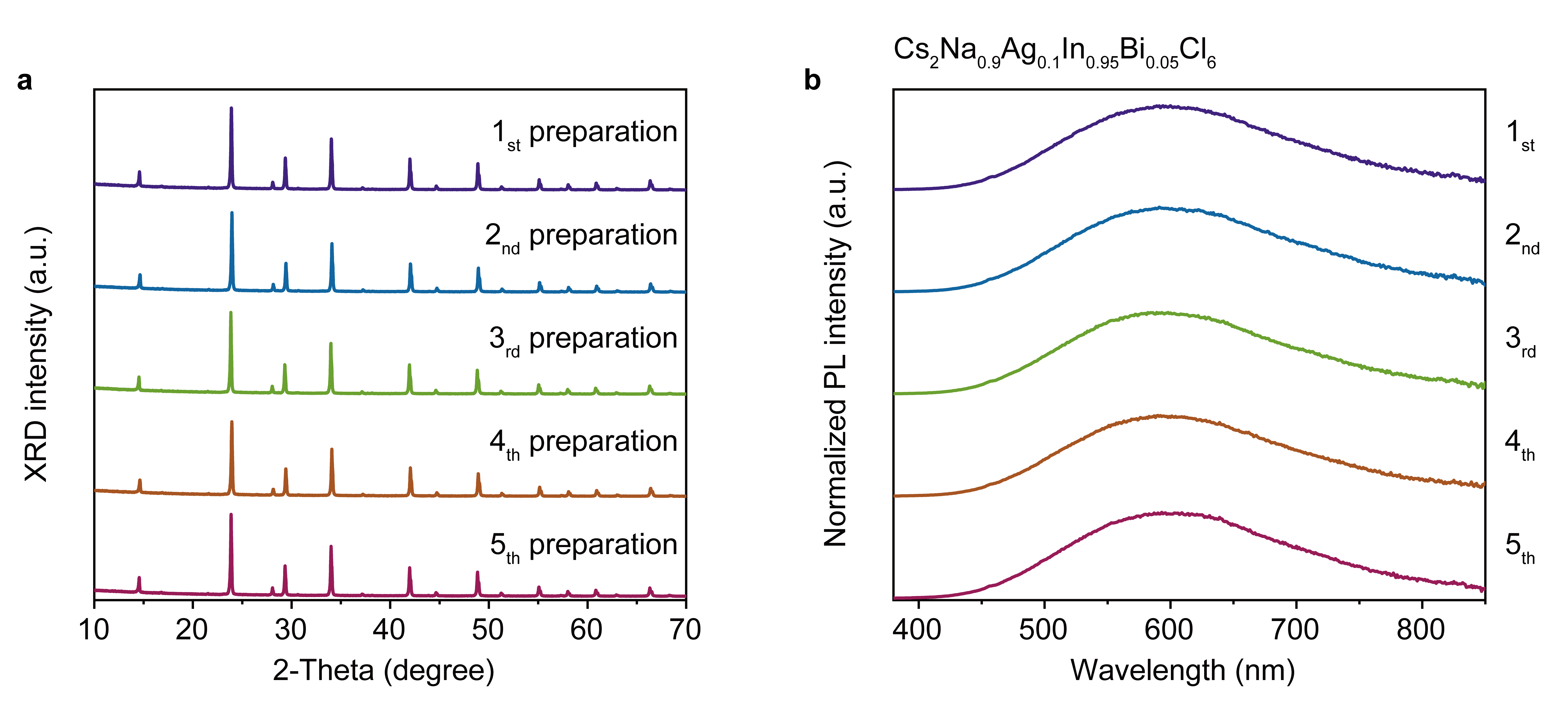
**

**Fig. S17. Cs_2_Na_0.9_Ag_0.1_In_0.95_Bi_0.05_Cl_6_ products synthesized by the HAAPP strategy in 5 days. (a)** XRD pattens and **(b)** PL spectra of 5 products synthesized in 5 days.

The PLQY values of these 5 products were measured to be 99.8%, 98.9%, 96.7%, 97.2%, 99.0%, respectively.


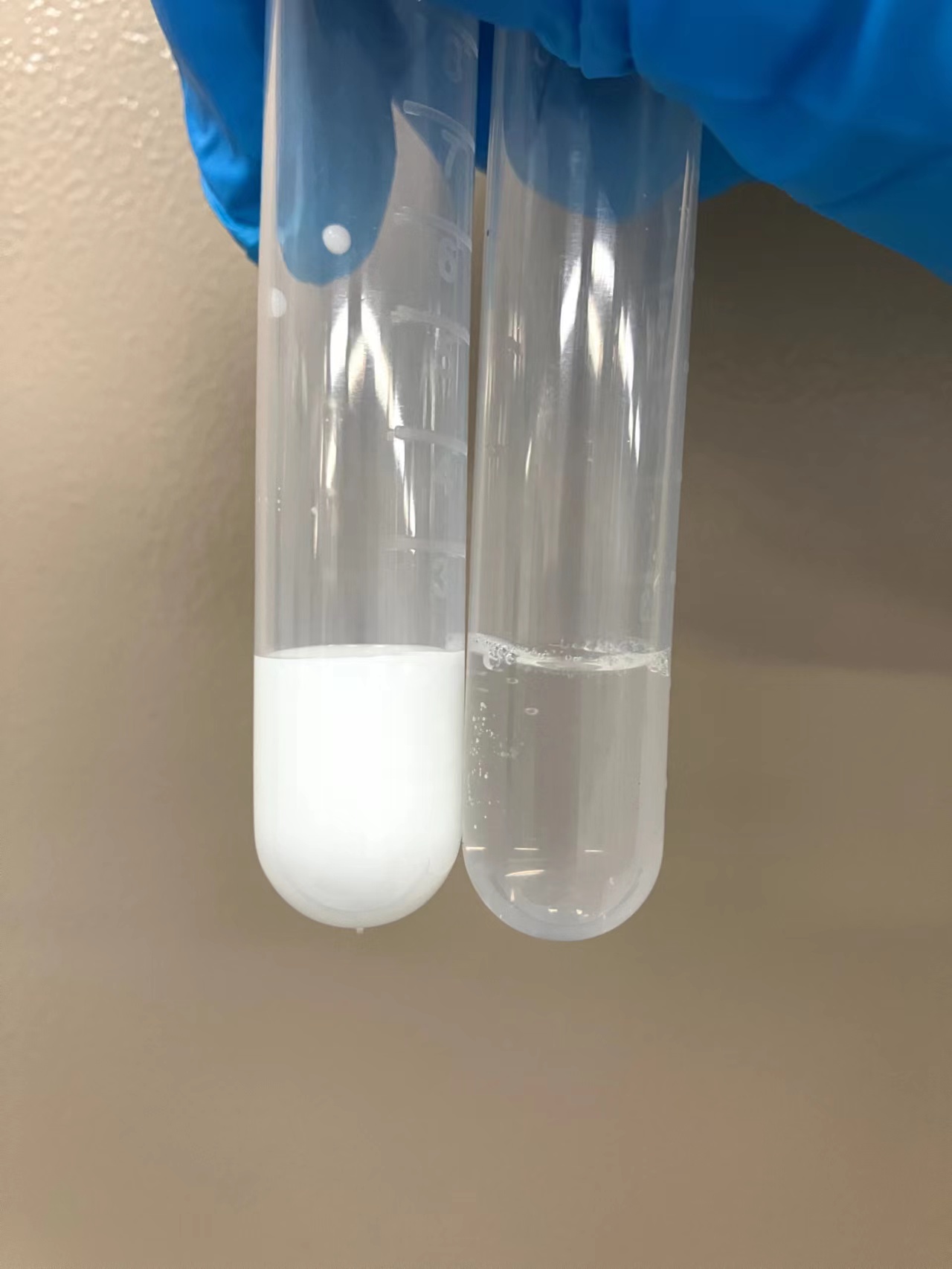


**Fig. S18. Cs_2_Na_0.9_Ag_0.1_In_0.95_Bi_0.05_Cl_6_ in concentrated (35 wt%, left) and diluted (5 wt%, right) HCl solution.**

**
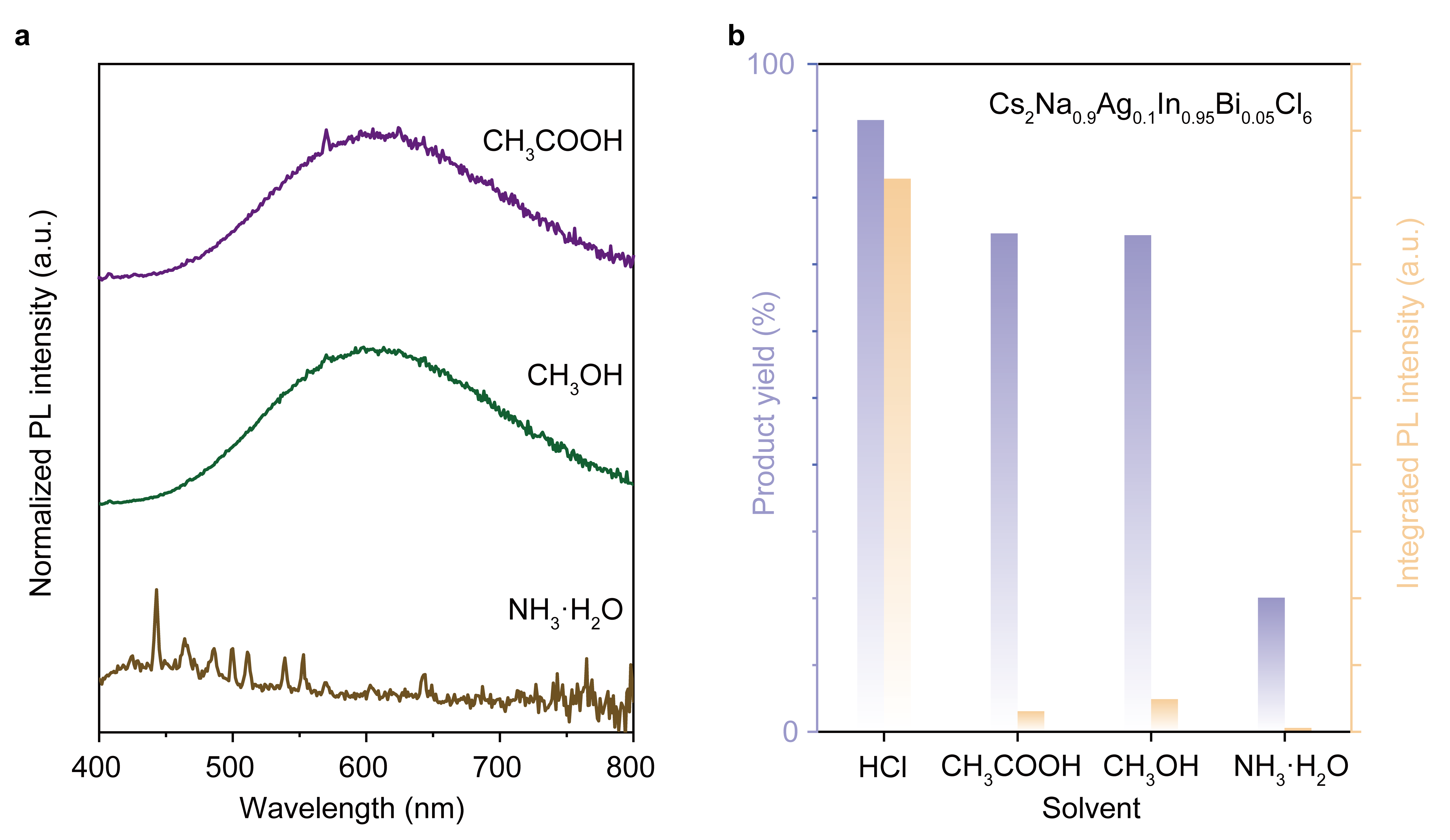
**

**Fig. S19. Fluorescence and product yields of Cs_2_Na_0.9_Ag_0.1_In_0.95_Bi_0.05_Cl_6_ synthesized with different solvents. (a)** PL spectra; **(b)** Product yields and integrated PL intensities.

**
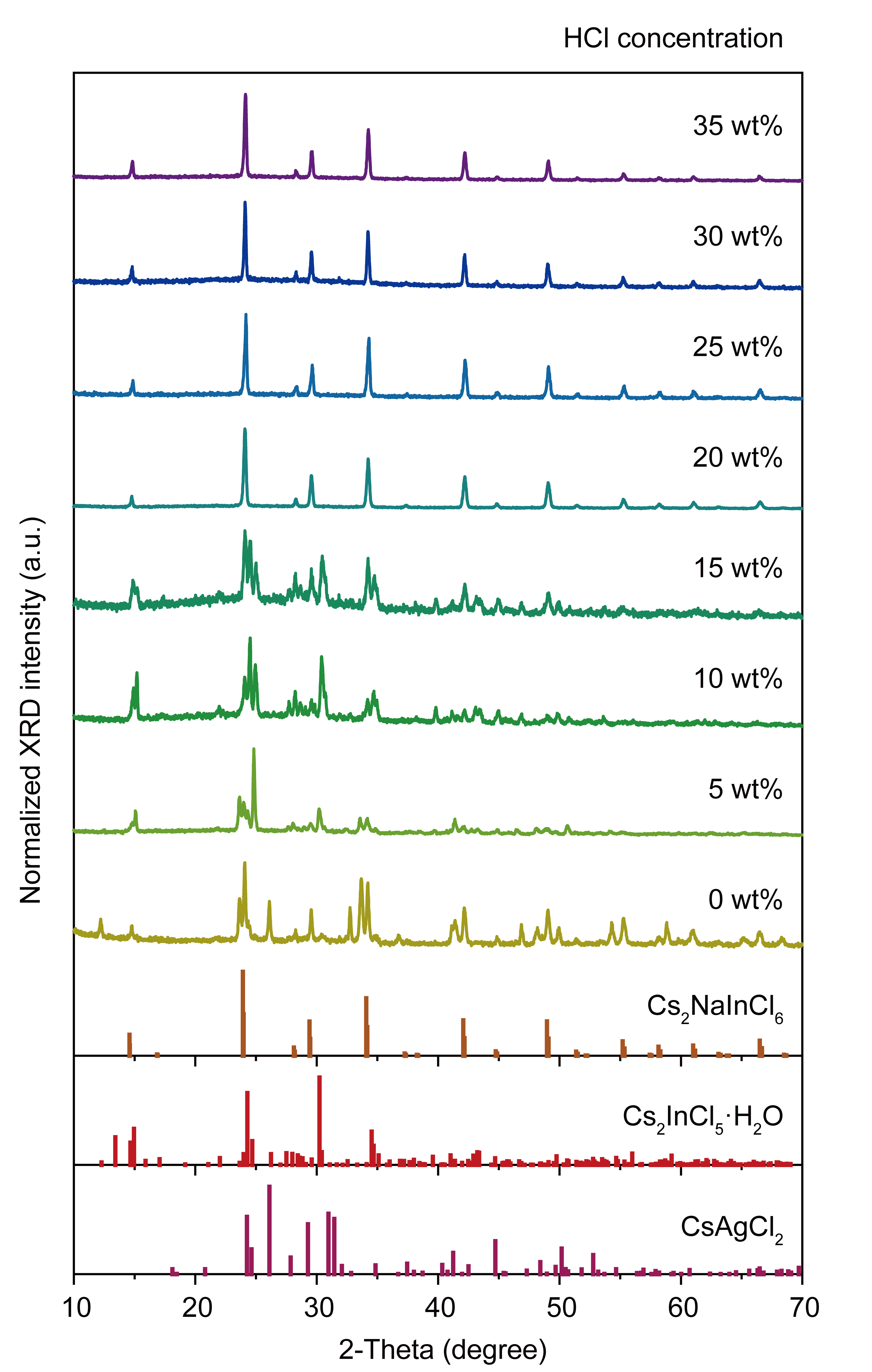
**

**Fig. S20. XRD patterns for Cs_2_Na_0.9_Ag_0.1_In_0.95_Bi_0.05_Cl_6_ synthesized with different concentration of HCl (35-0 wt%). The last 3 patterns are the simulated Cs_2_NaInCl_6_ crystal and the standard PDF cards for Cs_2_InCl_5_·H_2_O (JCPDS 78-1822) and CsAgCl_2_ (JCPDS 76-2238).**

**
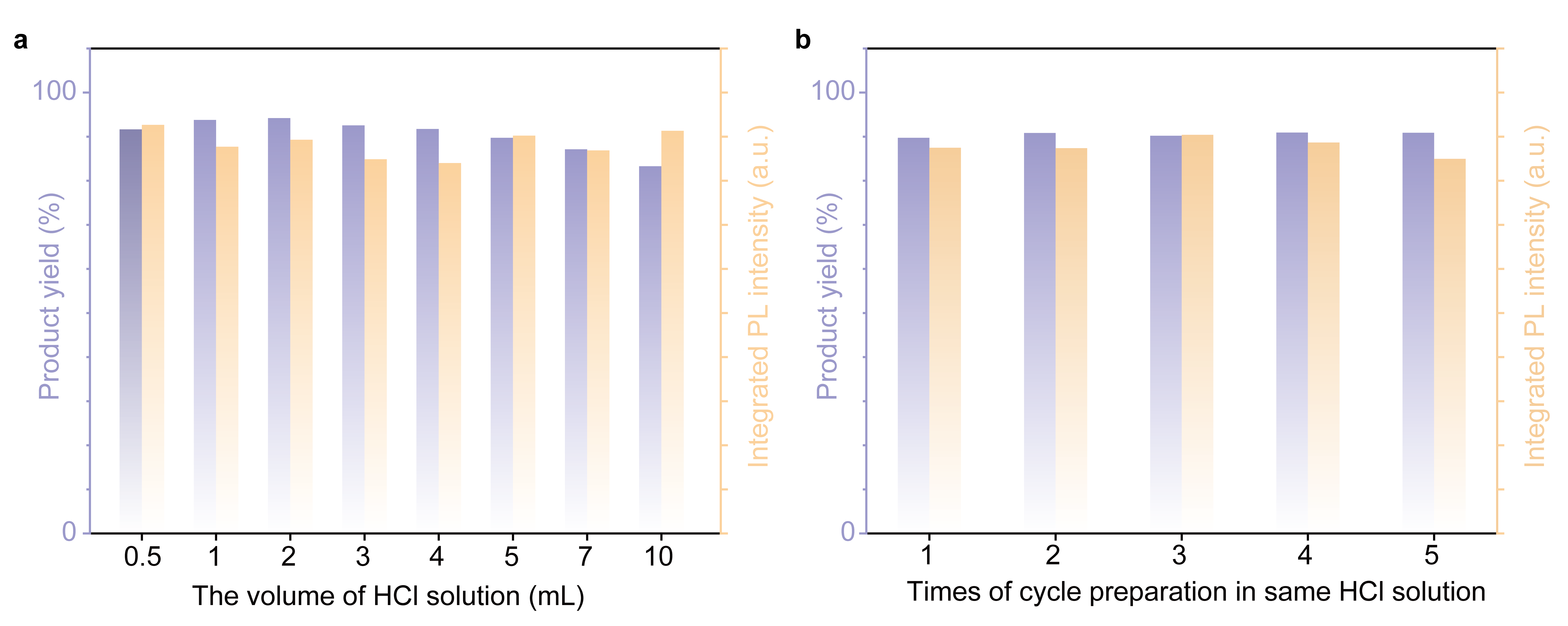
**

**Fig. S21. For Cs_2_Na_0.9_Ag_0.1_In_0.95_Bi_0.05_Cl_6_.** Product yields and PL intensity of products synthesized **(a)** with different volume of HCl or **(b)** with the same HCl solution (1 mL) after different times of cycle preparation.

Notably, from the Fig. 2g and Fig. S21, the maxima of product yield reaches ~95%, the other ~5% may exist in the solution as free ions through the equation S7:

|  | Cs_2_ABCl_6_ $\rightleftharpoons$ 2Cs^+^ + A^+^ + B^3+^ + 6Cl^-^ | (S7) |
| --- | --- | --- |

More free ions can be accommodated if increasing the volume of HCl solution, so that the product yield shows a slow downward trend when HCl solution exceeds 2 mL.


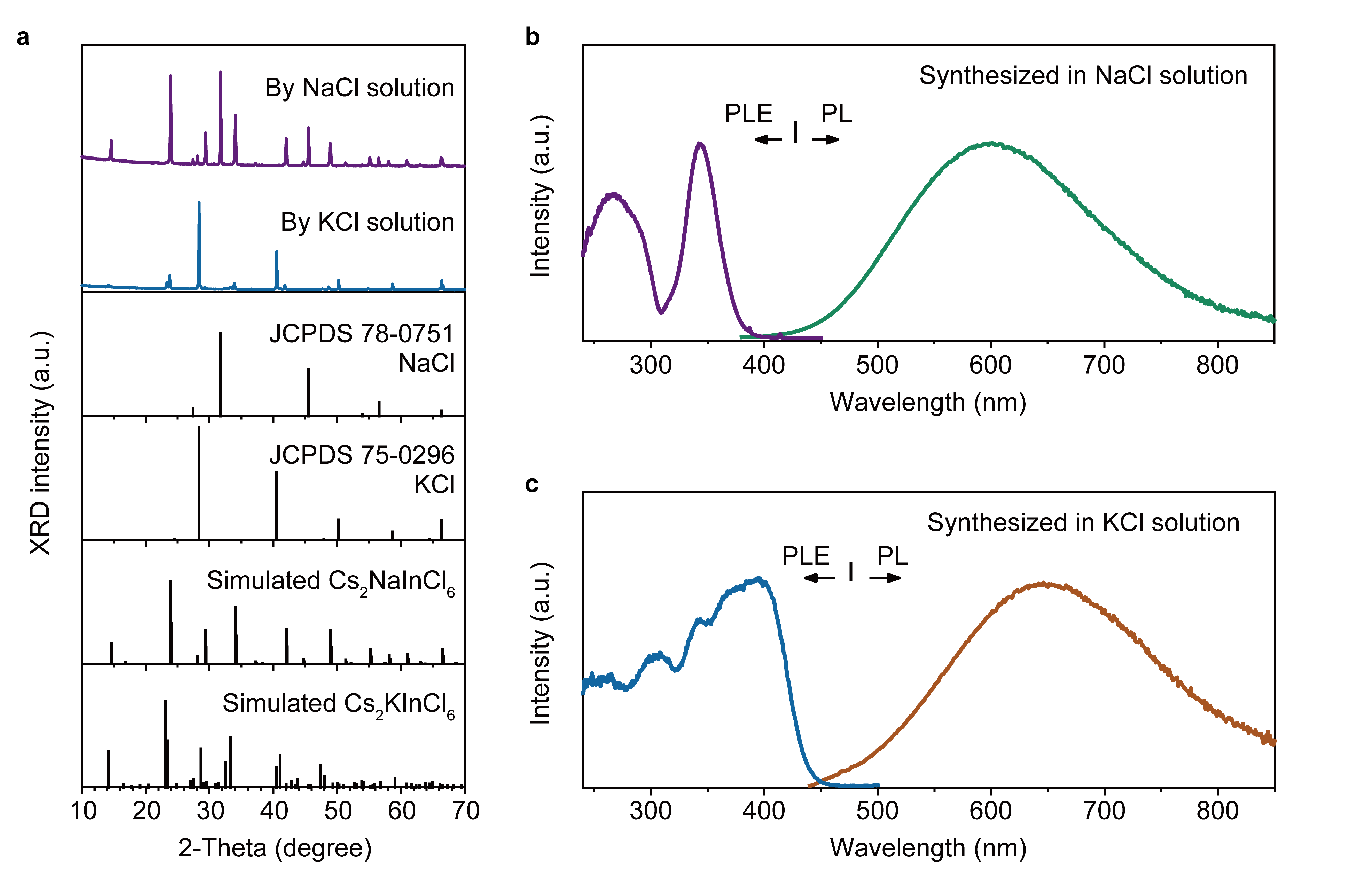


**Fig. S22. XRD patterns and fluorescent spectra. (a)** XRD patterns, PLE and PL spectra of products synthesized in saturated **(b)** NaCl and **(c)** KCl solutions.

The products synthesized with the saturated NaCl and KCl solutions showed PLQY of ~51.5% and ~13.6%, respectively. The low PLQY values may be caused by the impurities in products. The specific ratios of Na (or K) and Ag are shown in Tables S8 and S9.


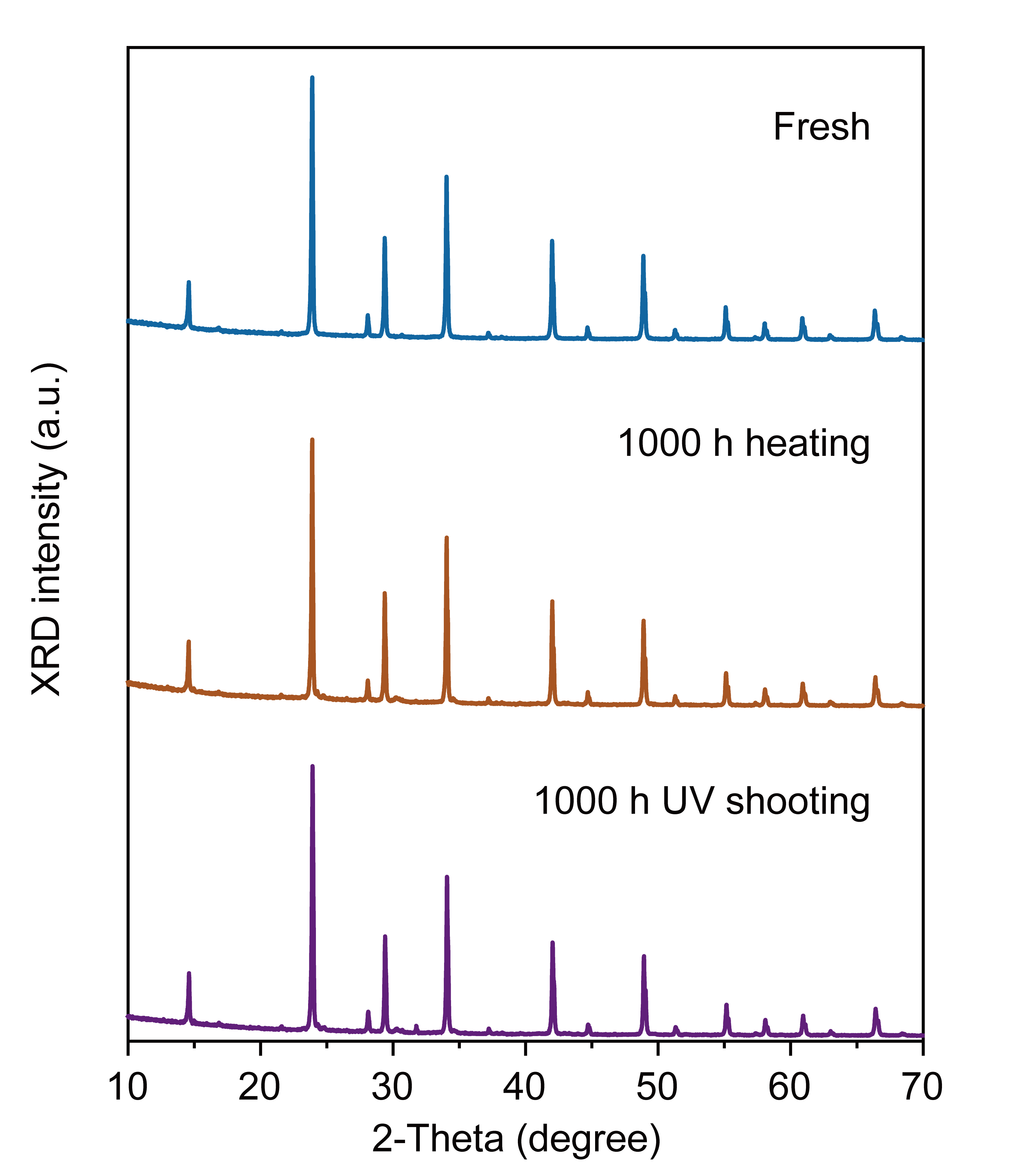


**Fig. S23. XRD patterns for Cs_2_Na_0.9_Ag_0.1_In_0.95_Bi_0.05_Cl_6_ products.** Fresh sample, after thermal- and photo-stability tests.


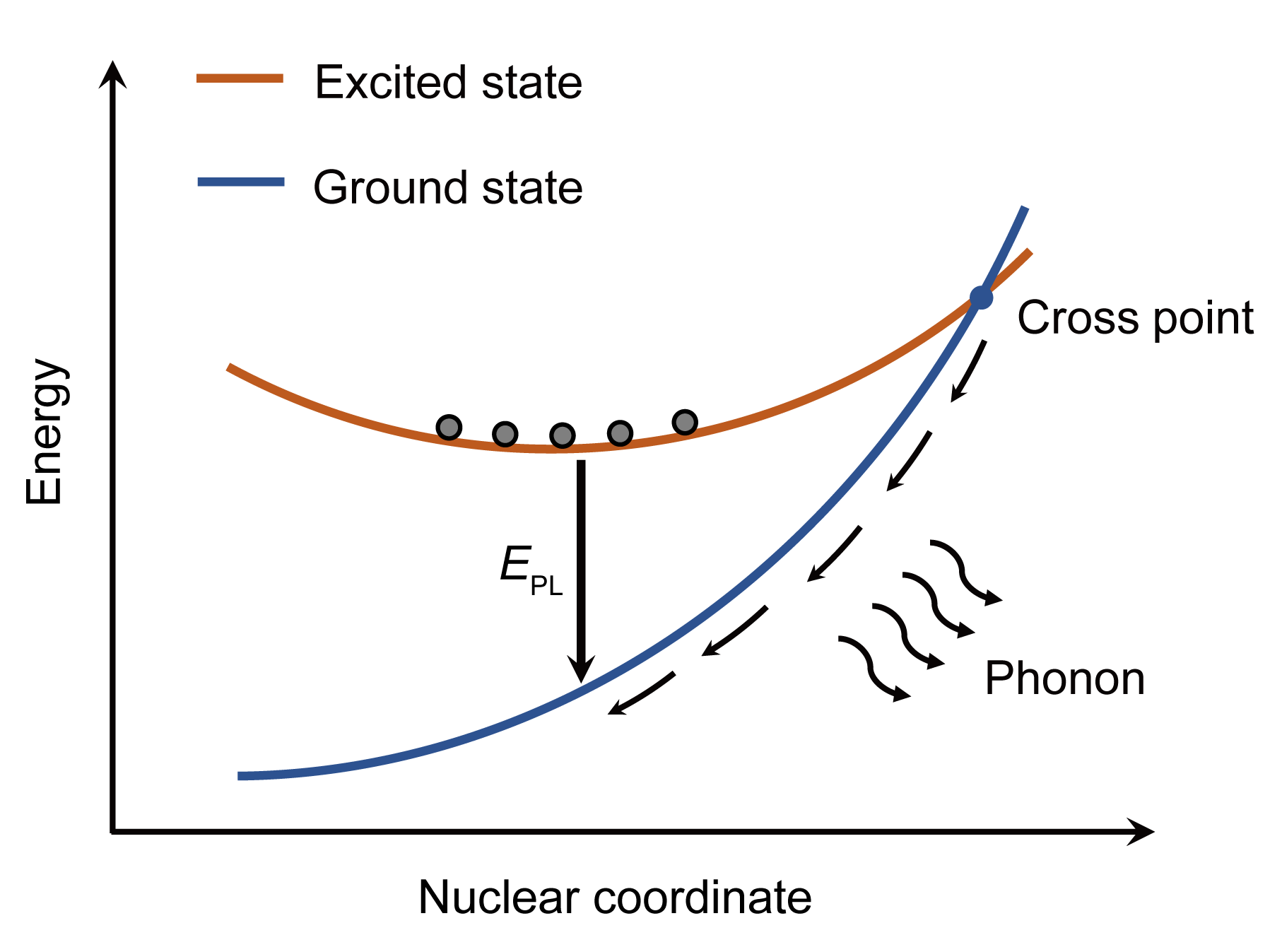


**Fig. S24. Non-radiative transition schematic.**

Generally, the PL emission is attributed to the photon energy release during the transition of electrons from the excited state to the ground state. Some electrons will return to the ground state through the cross point while raising the temperature, resulting in thermal quenching (ref. 4 in the main text).


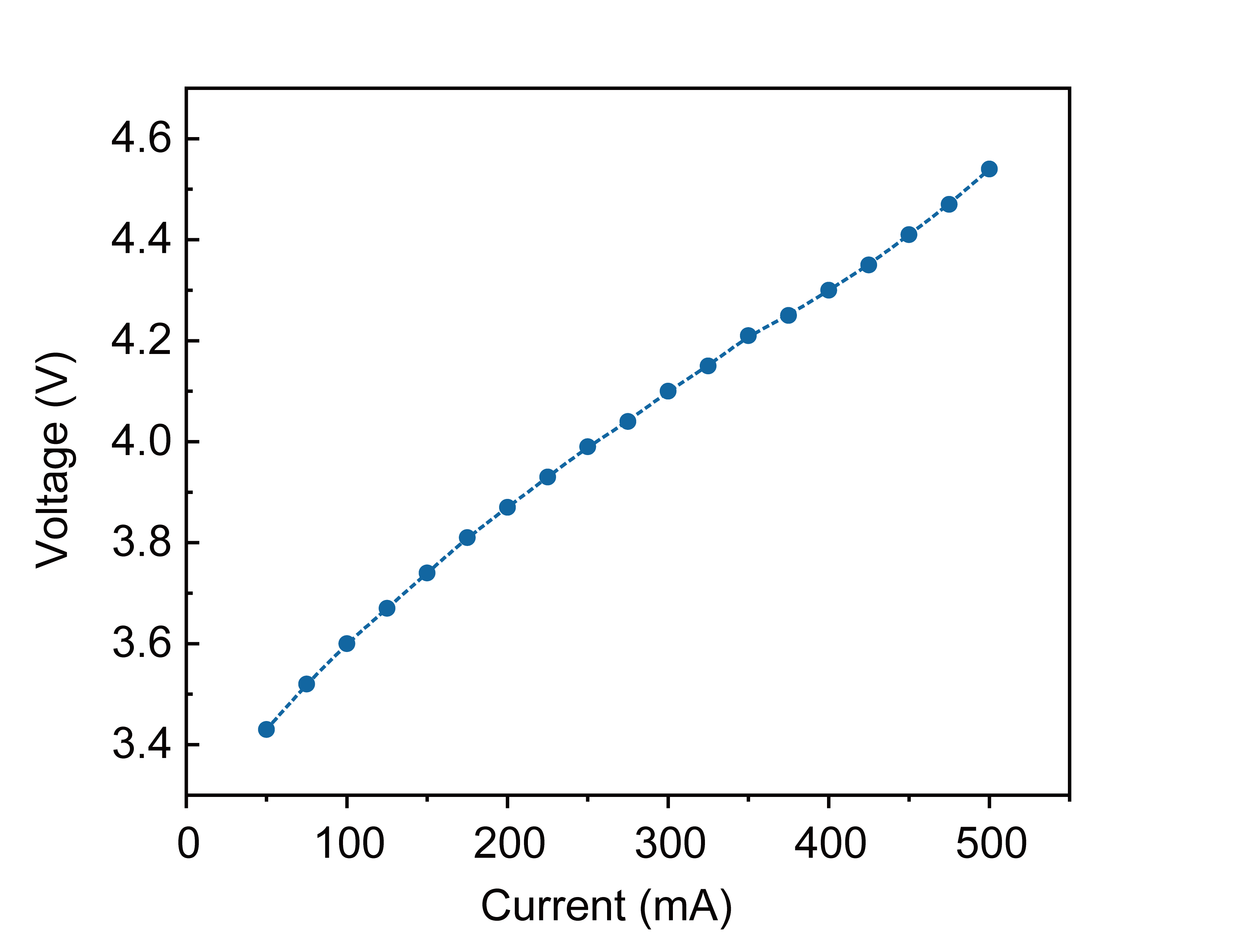


**Fig. S25. Voltage of pc-LED device as a function of input current.**


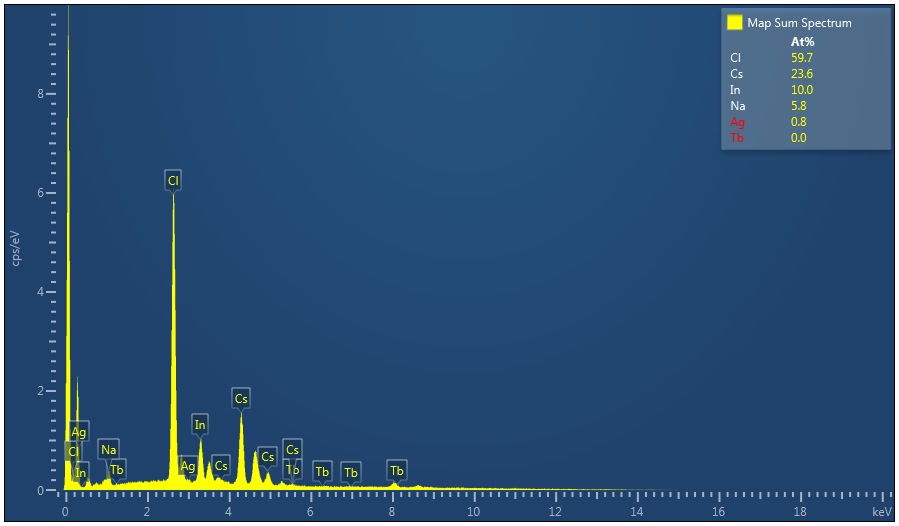


**Fig. S26. EDS spectrum of Cs_2_Na_0.9_Ag_0.1_InCl_6_:10%Tb^3+^ for Fig. 4d.**


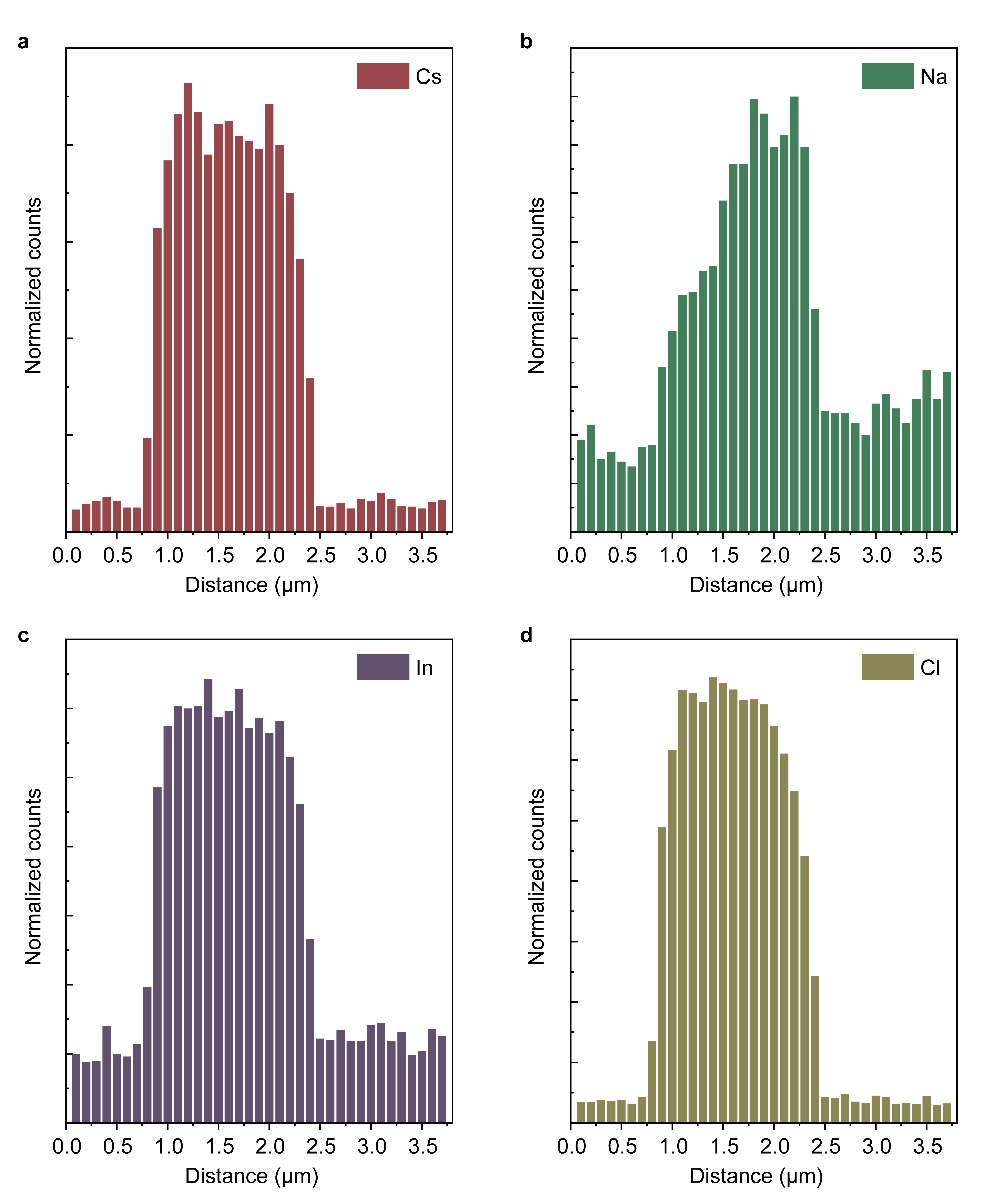


**Fig. S27. EDS profile of the line scanning marked in Fig. 4d.** For **(a)** Cs, **(b)** Na, **(c)** In and **(d)** Cl elements.

**
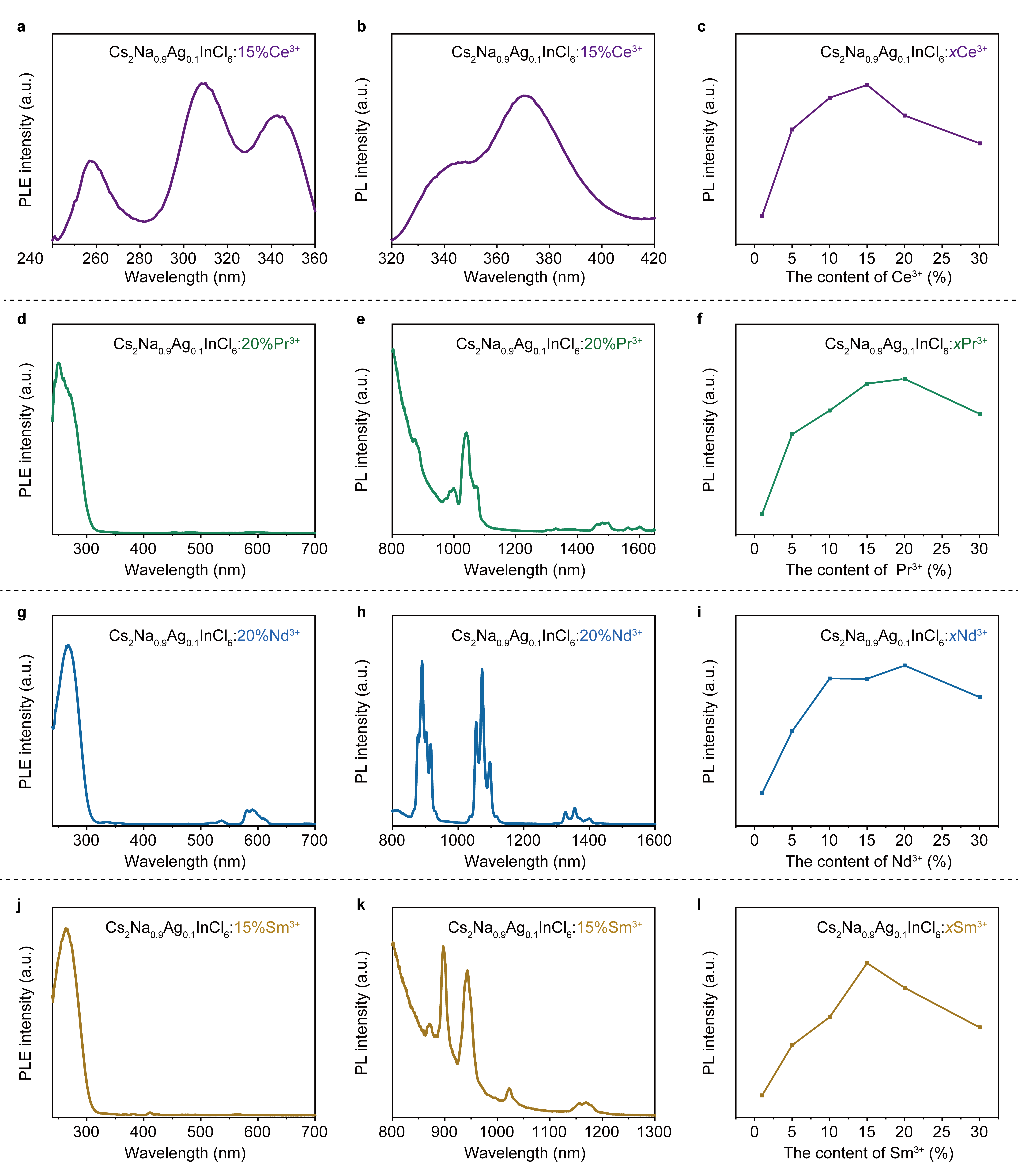
**

**Fig. S28. Ions-doped Cs_2_Na_0.9_Ag_0.1_InCl_6_ phosphors.** Luminescence properties of **(a-c)** Ce^3+^, **(d-f)** Pr^3+^, **(g-i)** Nd^3+^ and **(j-l)** Sm^3+^.


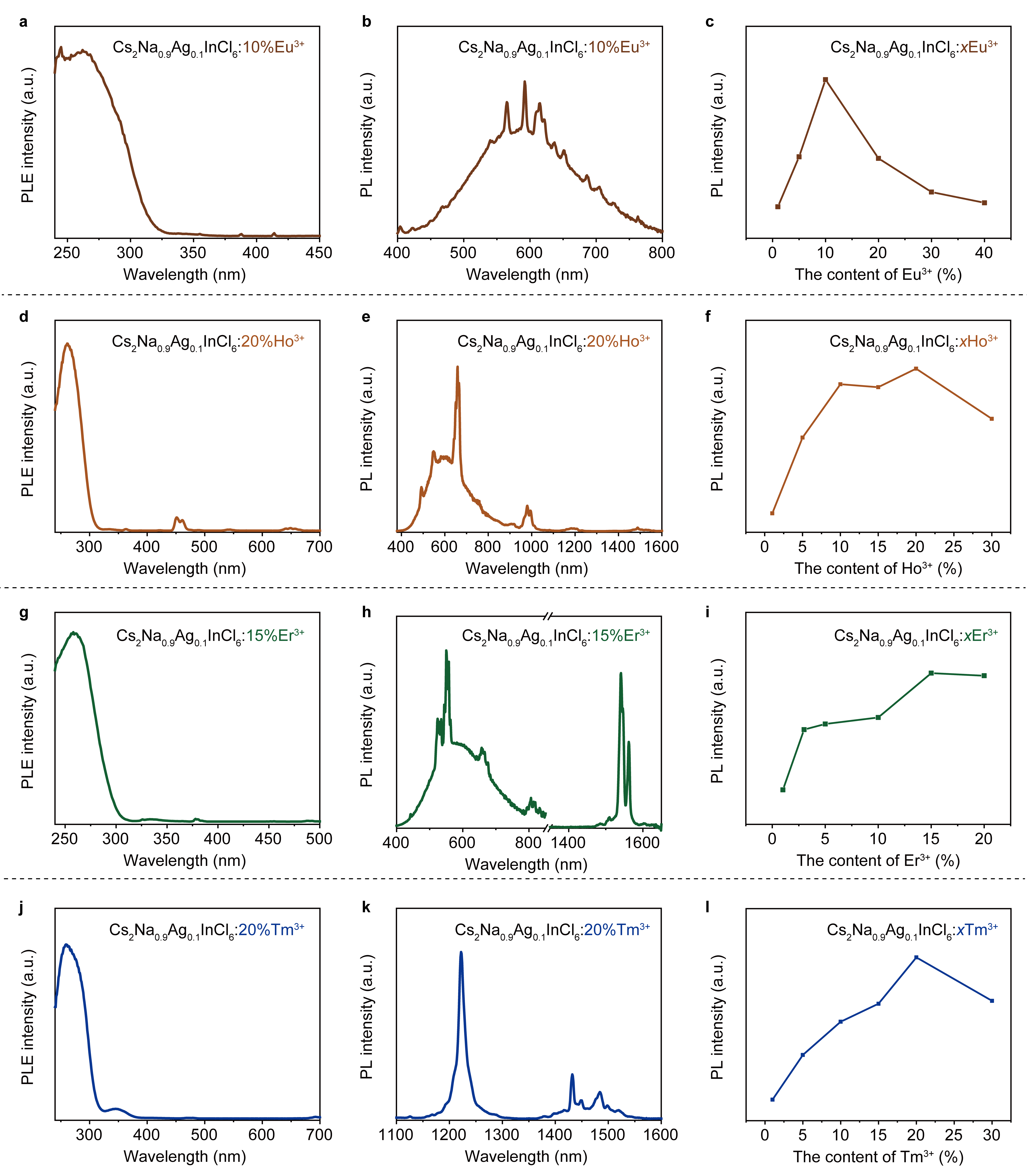


**Fig. S29. Ions-doped Cs_2_Na_0.9_Ag_0.1_InCl_6_ phosphors.** Luminescence properties of **(a-c)** Eu^3+^, **(d-f)** Ho^3+^, **(g-i)** Er^3+^ and **(j-l)** Tm^3+^.


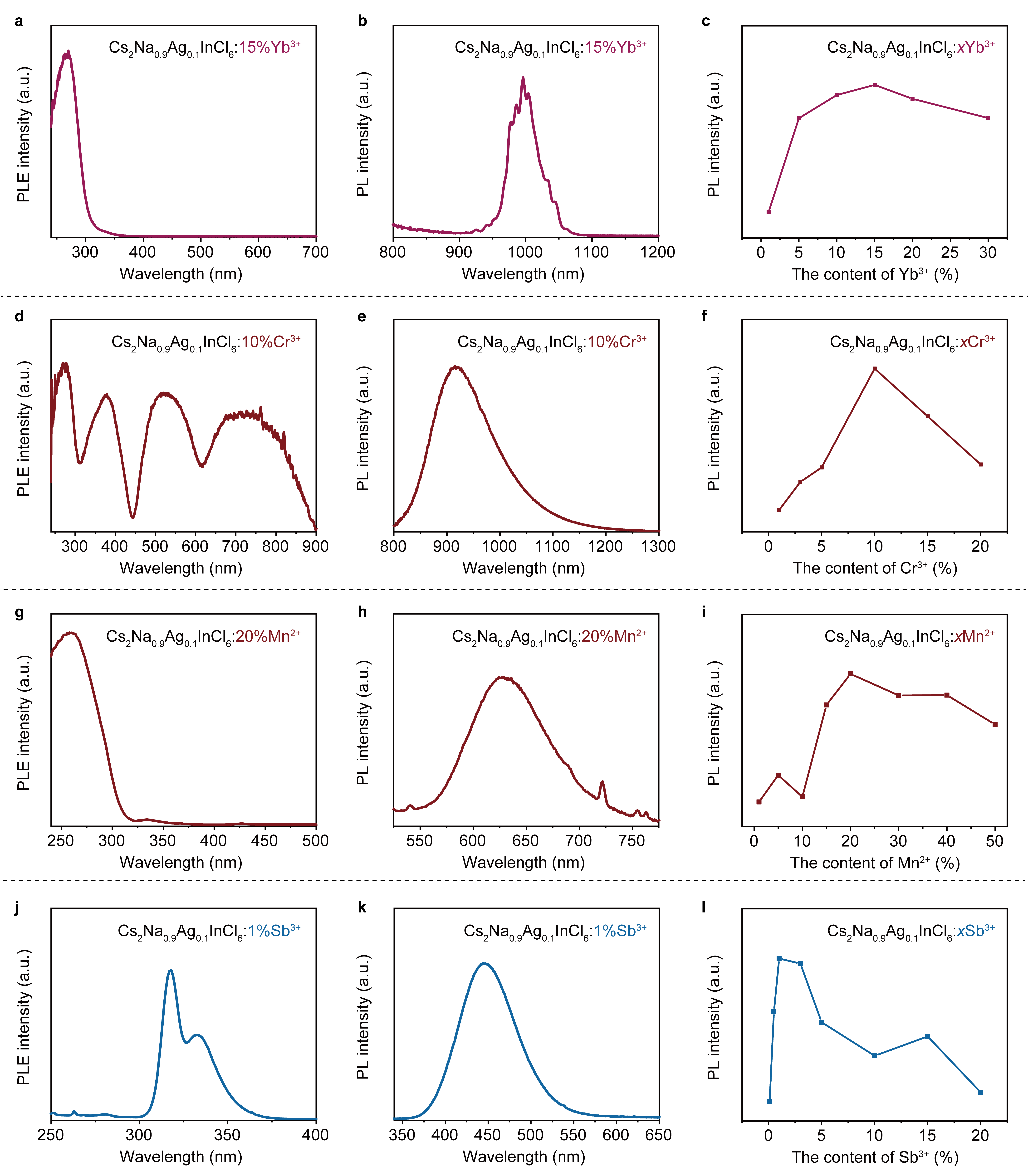


**Fig. S30. Ions-doped** **Cs_2_Na_0.9_Ag_0.1_InCl_6_ phosphors.** Luminescence properties of **(a-c)** Yb^3+^, **(d-f)** Cr^3+^, **(g-i)** Mn^2+^ and **(j-l)** Sb^3+^.

**
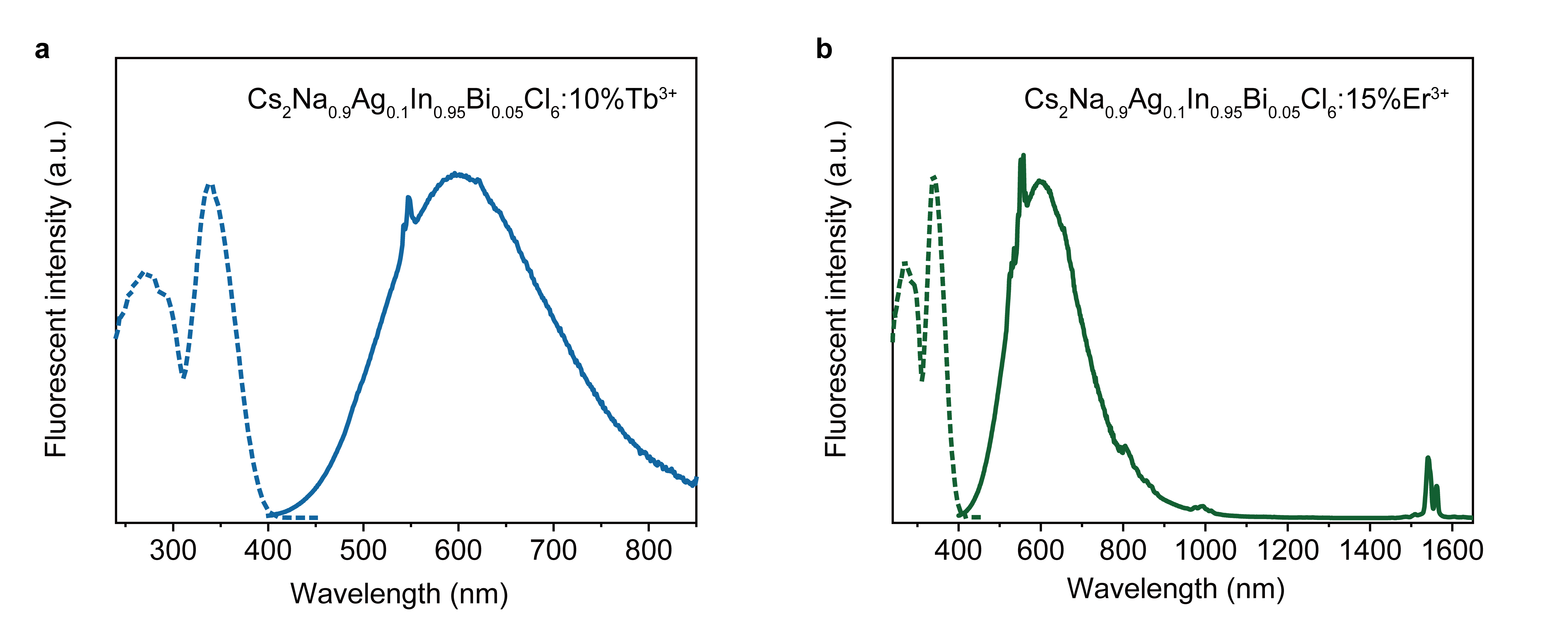
**

**Fig. S31. PL and PLE spectra of (a)** Cs_2_Na_0.9_Ag_0.1_In_0.95_Bi_0.05_Cl_6_:10%Tb^3+^ **and (b)** Cs_2_Na_0.9_Ag_0.1_In_0.95_Bi_0.05_Cl_6_:15%Er^3+^ **phosphors.**

**
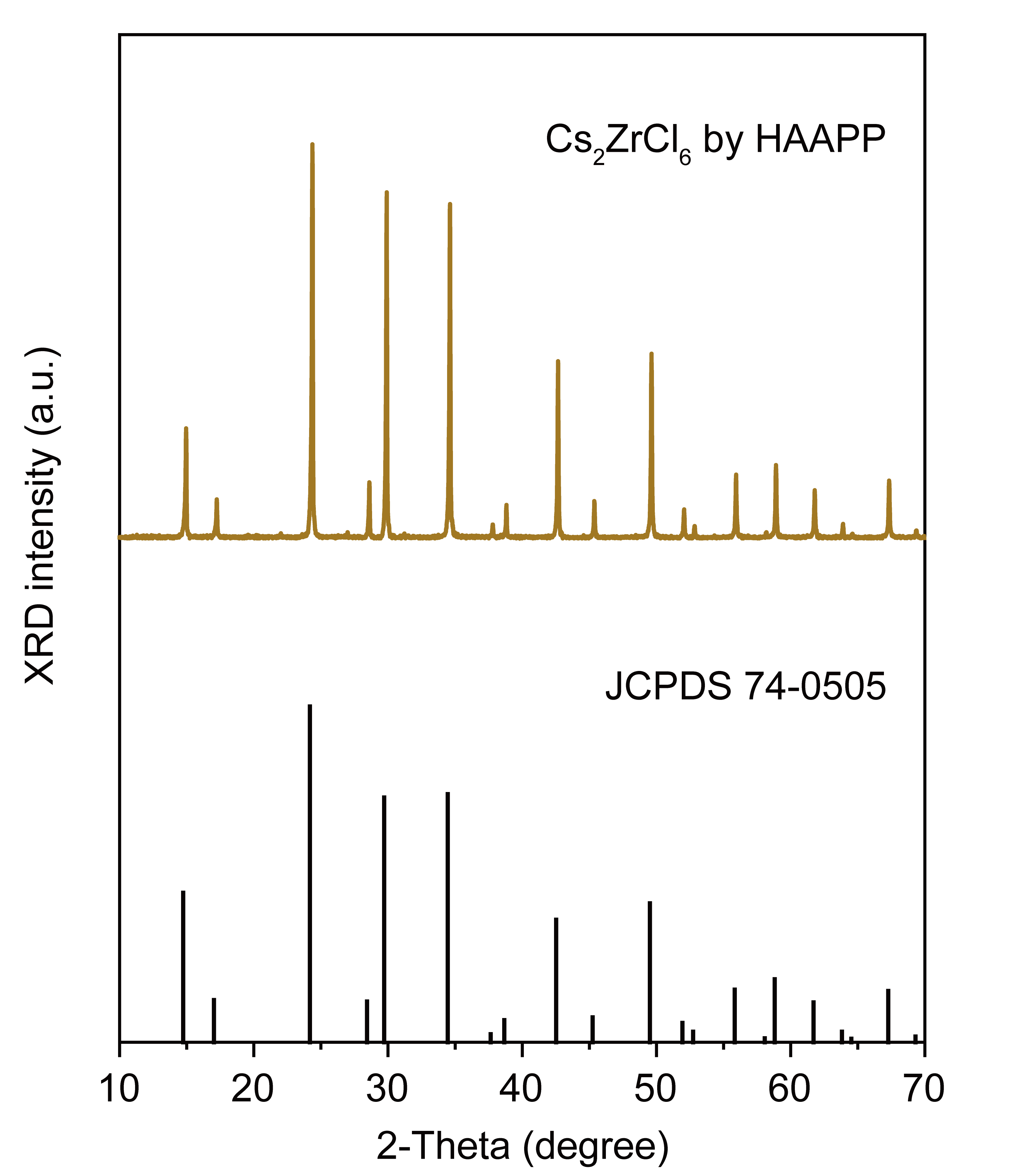
**

**Fig. S32. XRD pattern for Cs_2_ZrCl_6_ synthesized by HAAPP strategy.**

**
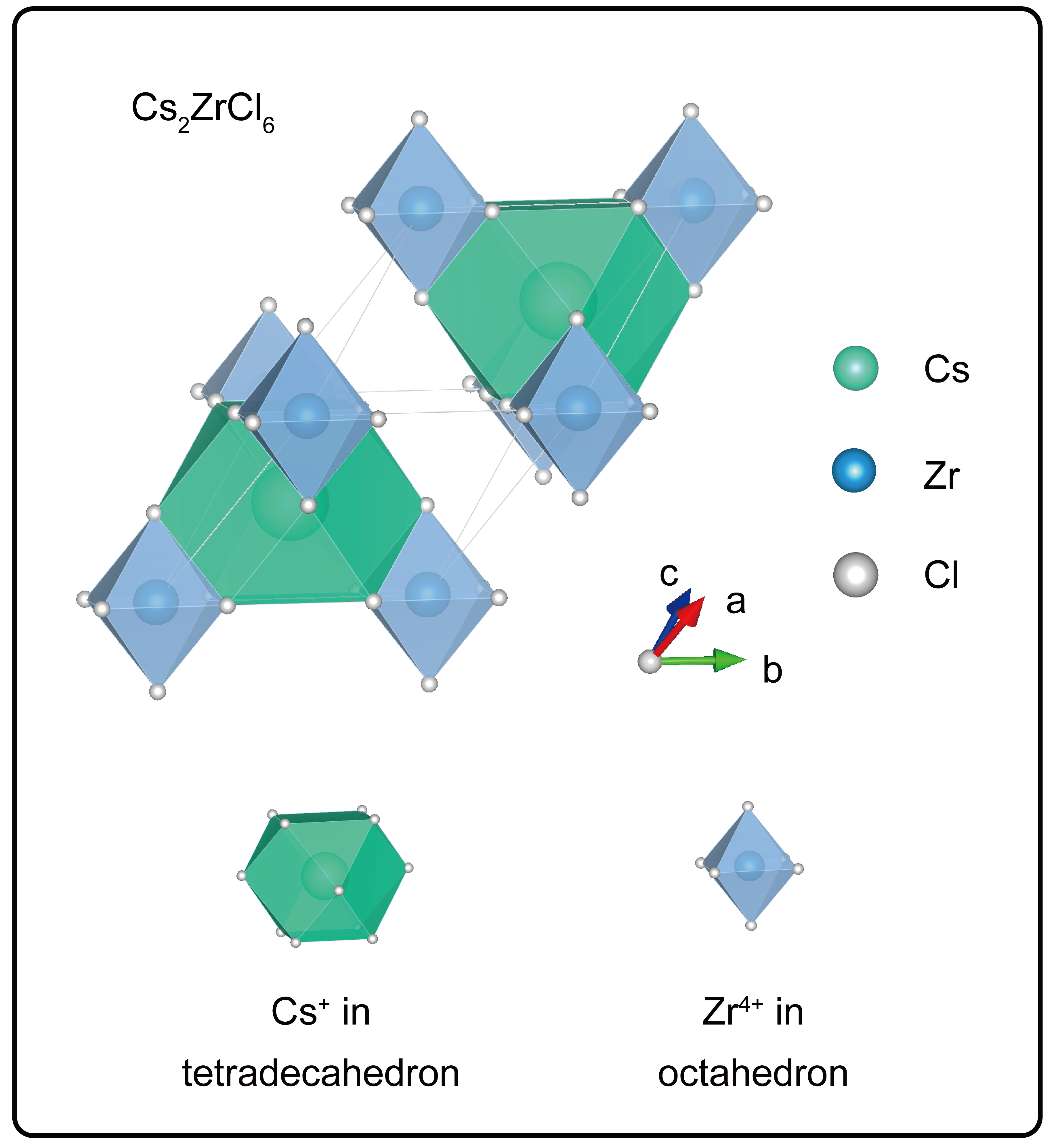
**

**Fig. S33. Standard crystal structure of Cs_2_ZrCl_6_.**

**
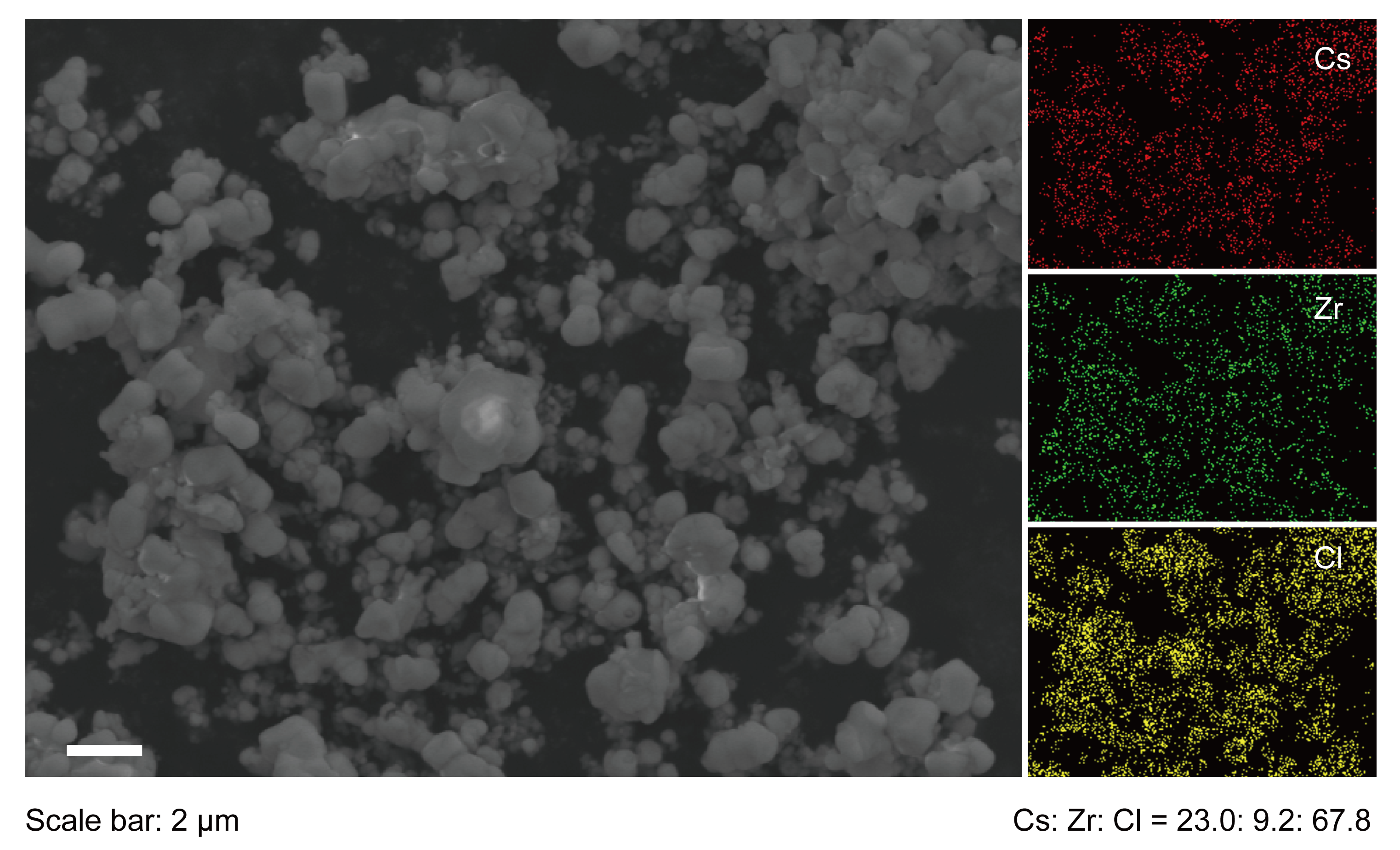
**

**Fig. S34. SEM image and elements mapping of Cs_2_ZrCl_6_ synthesized by the HAAPP strategy.**

The contents of Cs and Zr were further confirmed as 197.91% and 93.90% by ICP, which are greatly in line with the feeding ratio of 2:1.


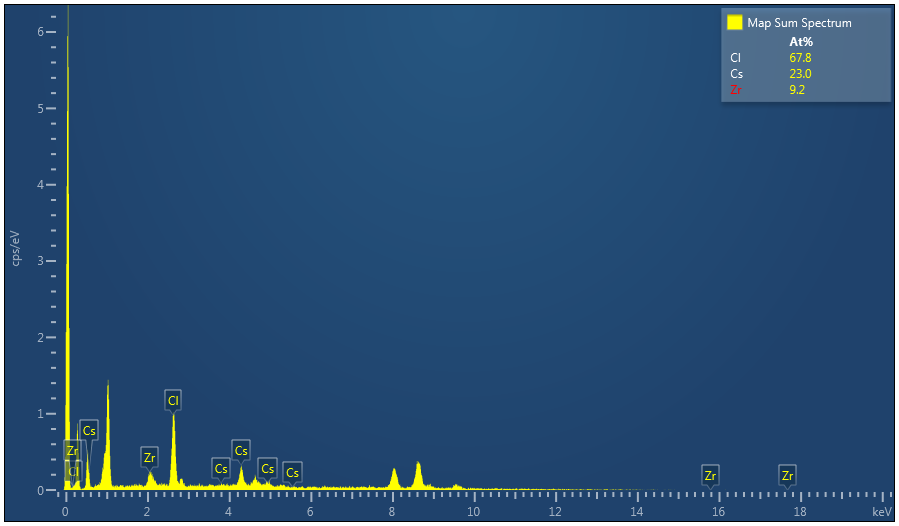


**Fig. S35. EDS spectrum of Cs_2_ZrCl_6_ for Fig. S34.**

**
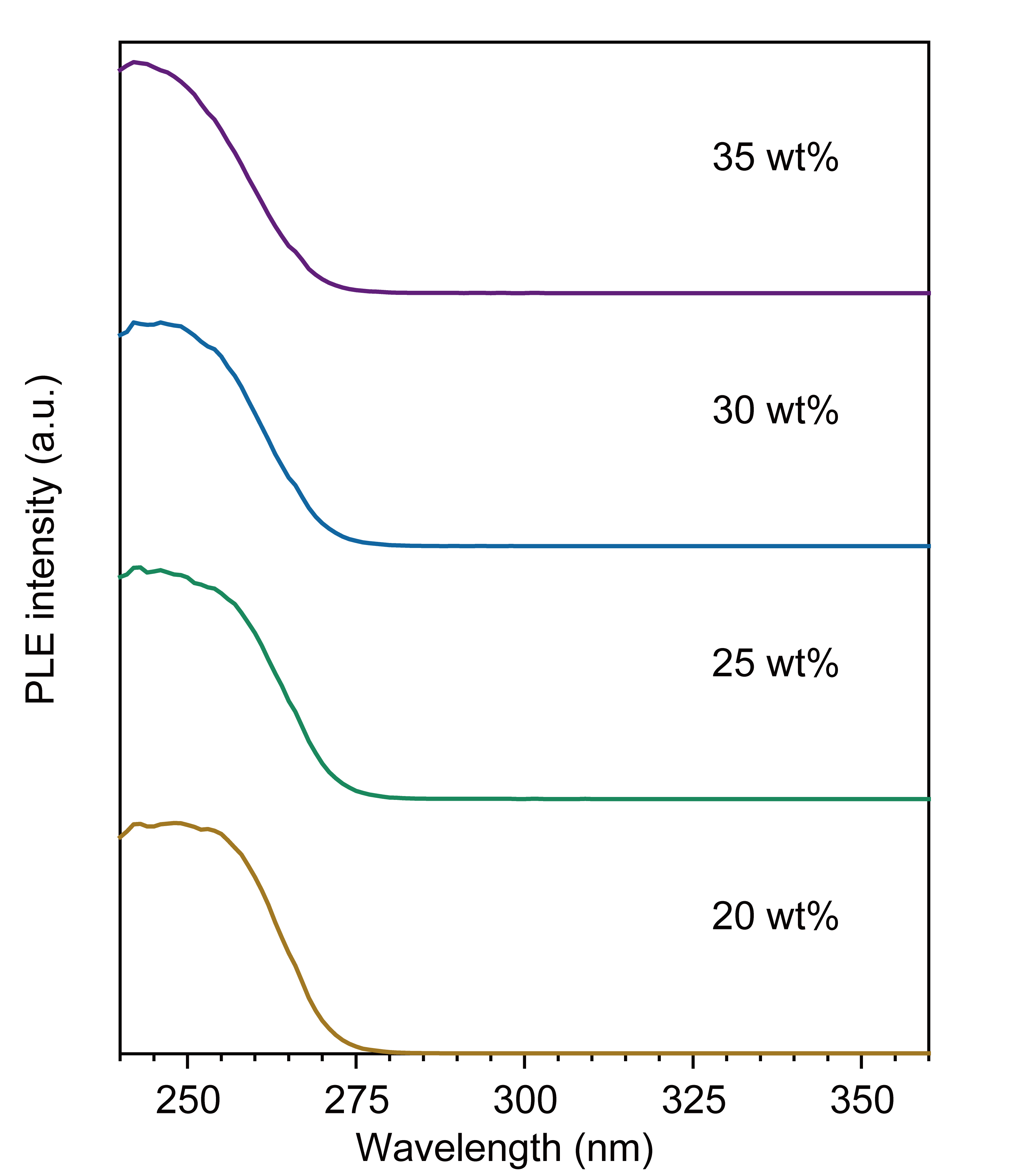
**

**Fig. S36. PLE spectra of Cs_2_ZrCl_6_ synthesized with different concentration of HCl.**

**
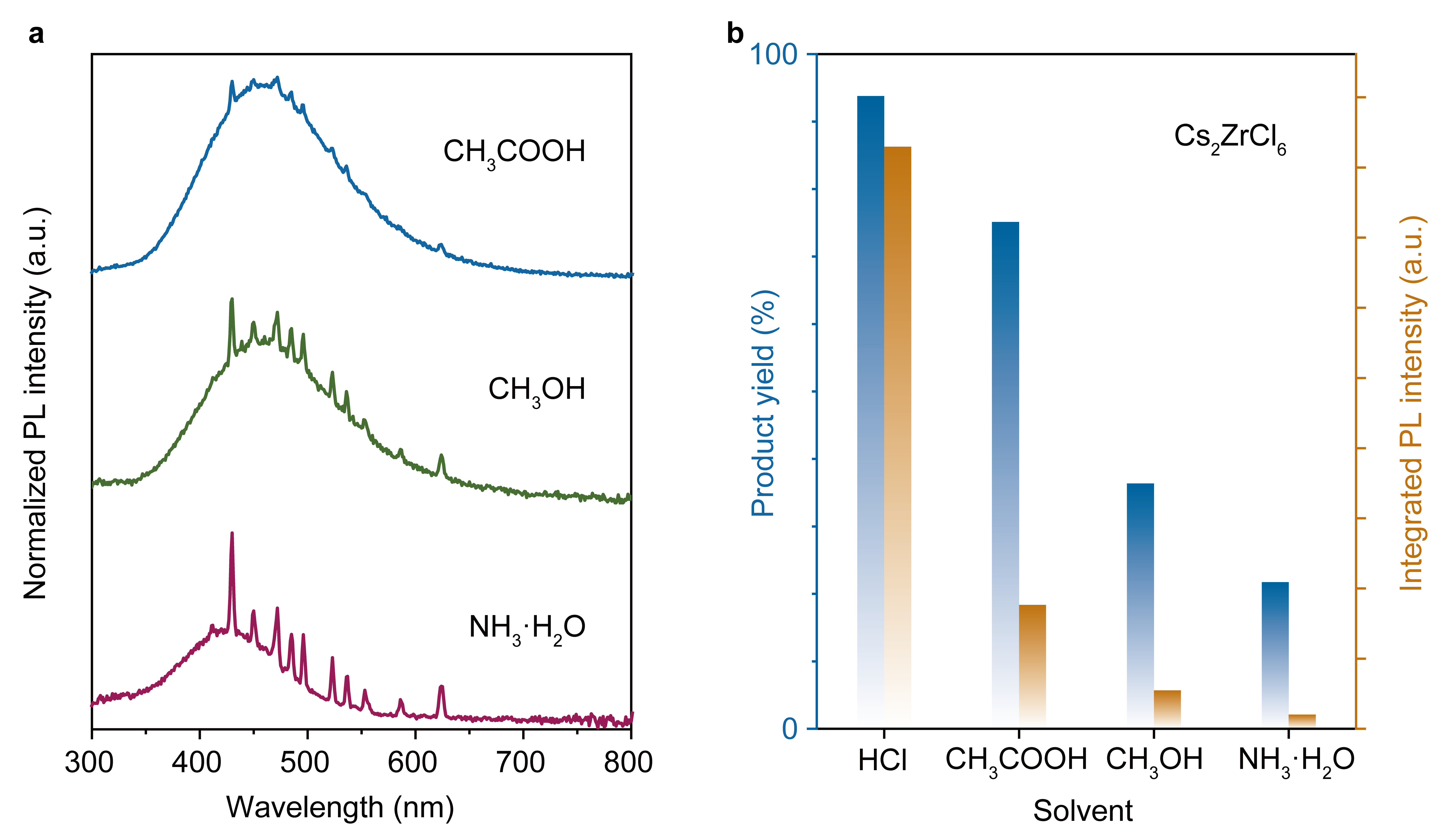
**

**Fig. S37. Fluorescence and product yields of Cs_2_ZrCl_6_ synthesized with different solvents. (a)** PL spectra; **(b)** Product yields and integrated PL intensities.


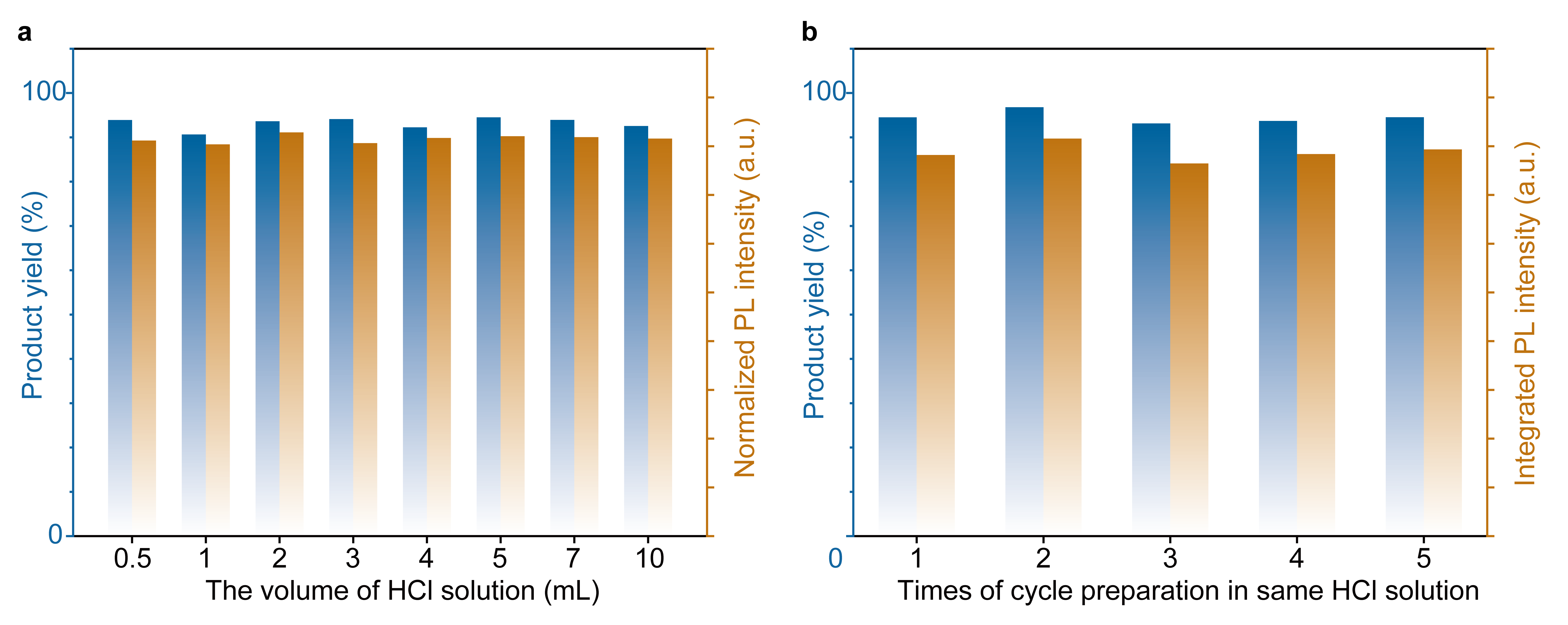


**Fig. S38. Product yields and PL intensities of Cs_2_ZrCl_6_. (a)** HAAPP strategy with different volume of HCl solution; **(b)** Product yields and integrated PL intensity of products synthesized with the same HCl solution (1 mL) after different times of cycle preparation.


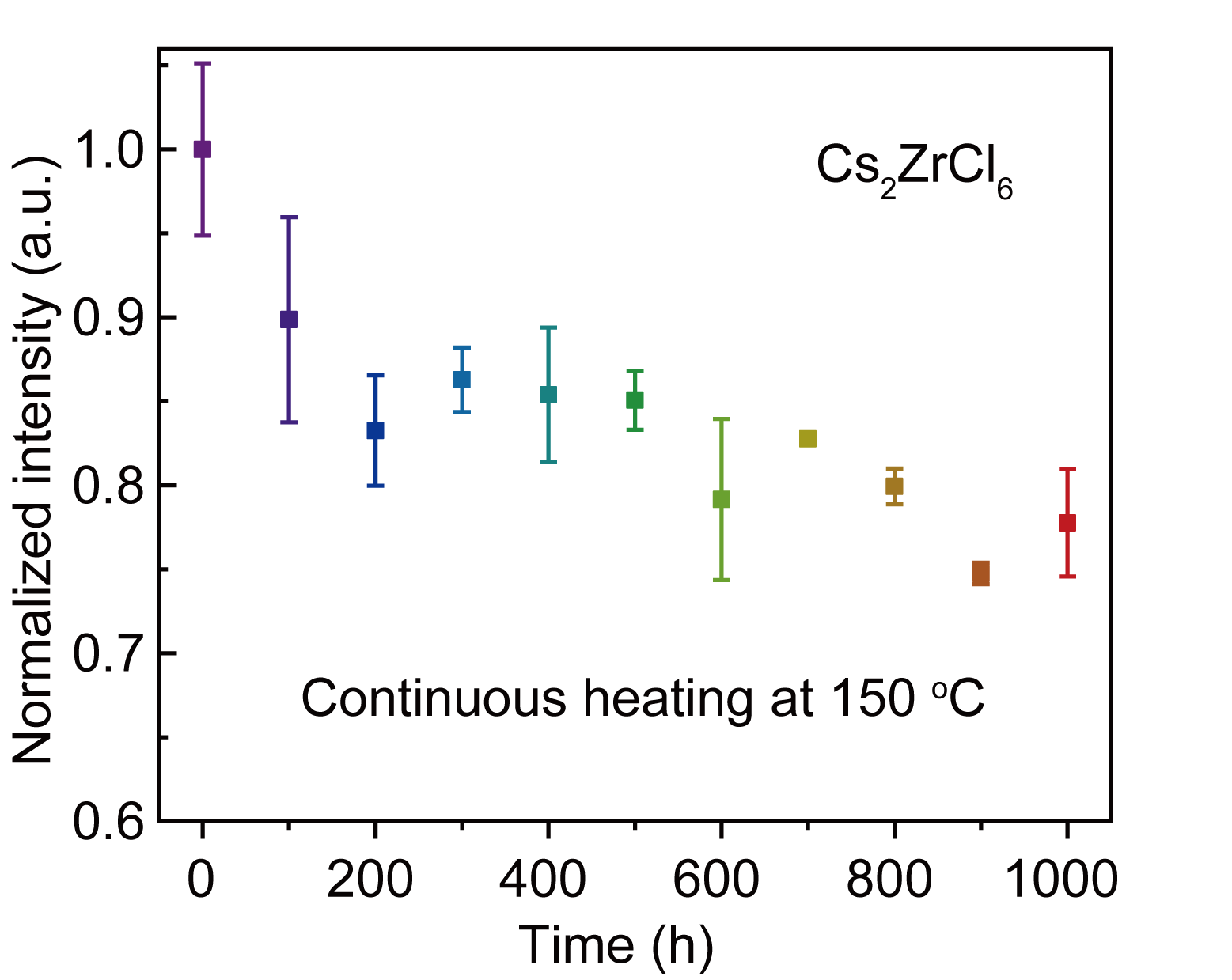


**Fig. S39. Thermal-stability of Cs_2_ZrCl_6_ after heated at 150 ^o^C for 1000 h, which are measured after cooling to the room temperature.**

**
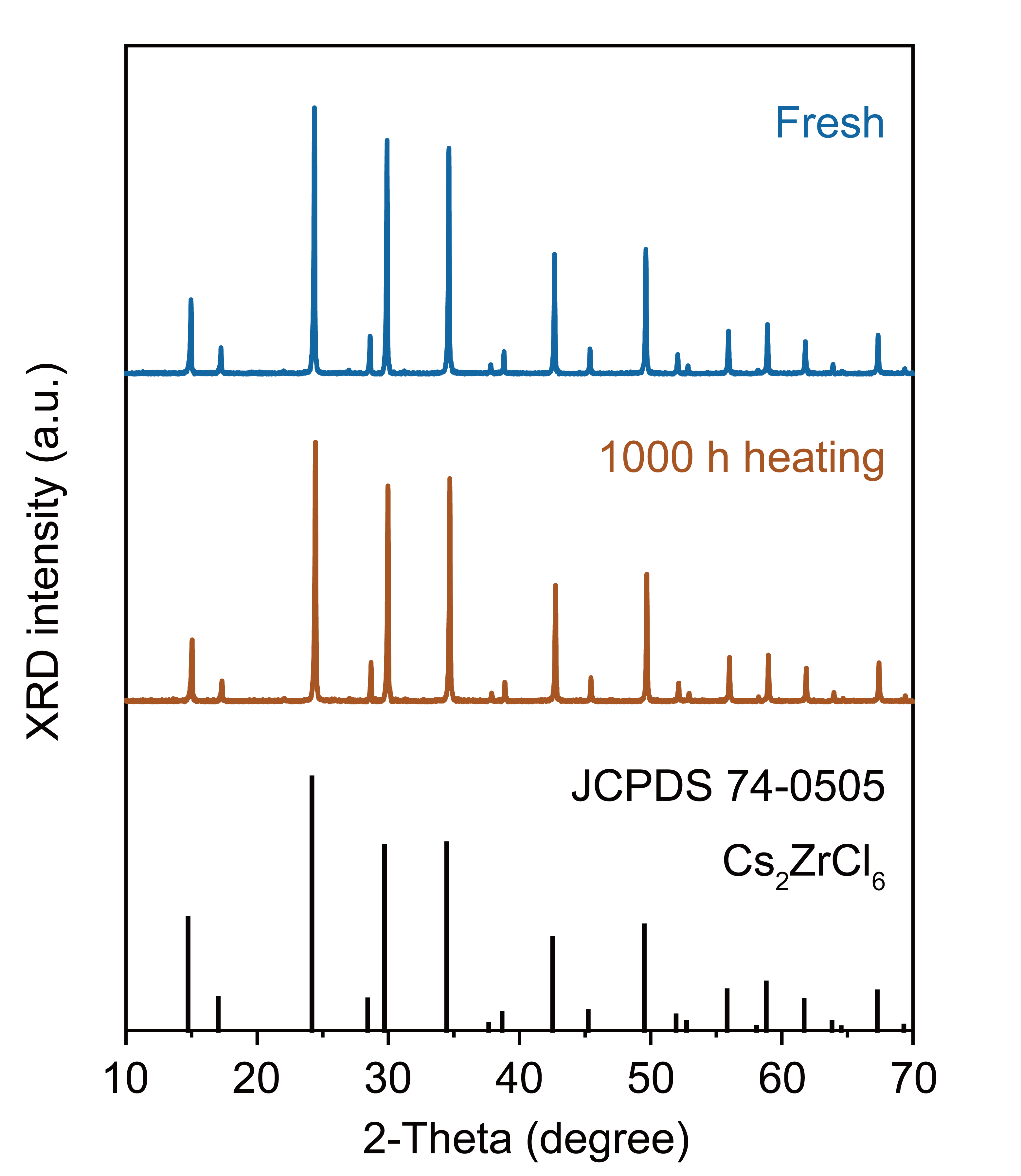
**

**Fig. S40. XRD patterns for fresh and 1000 h-heated Cs_2_ZrCl_6_.**

**
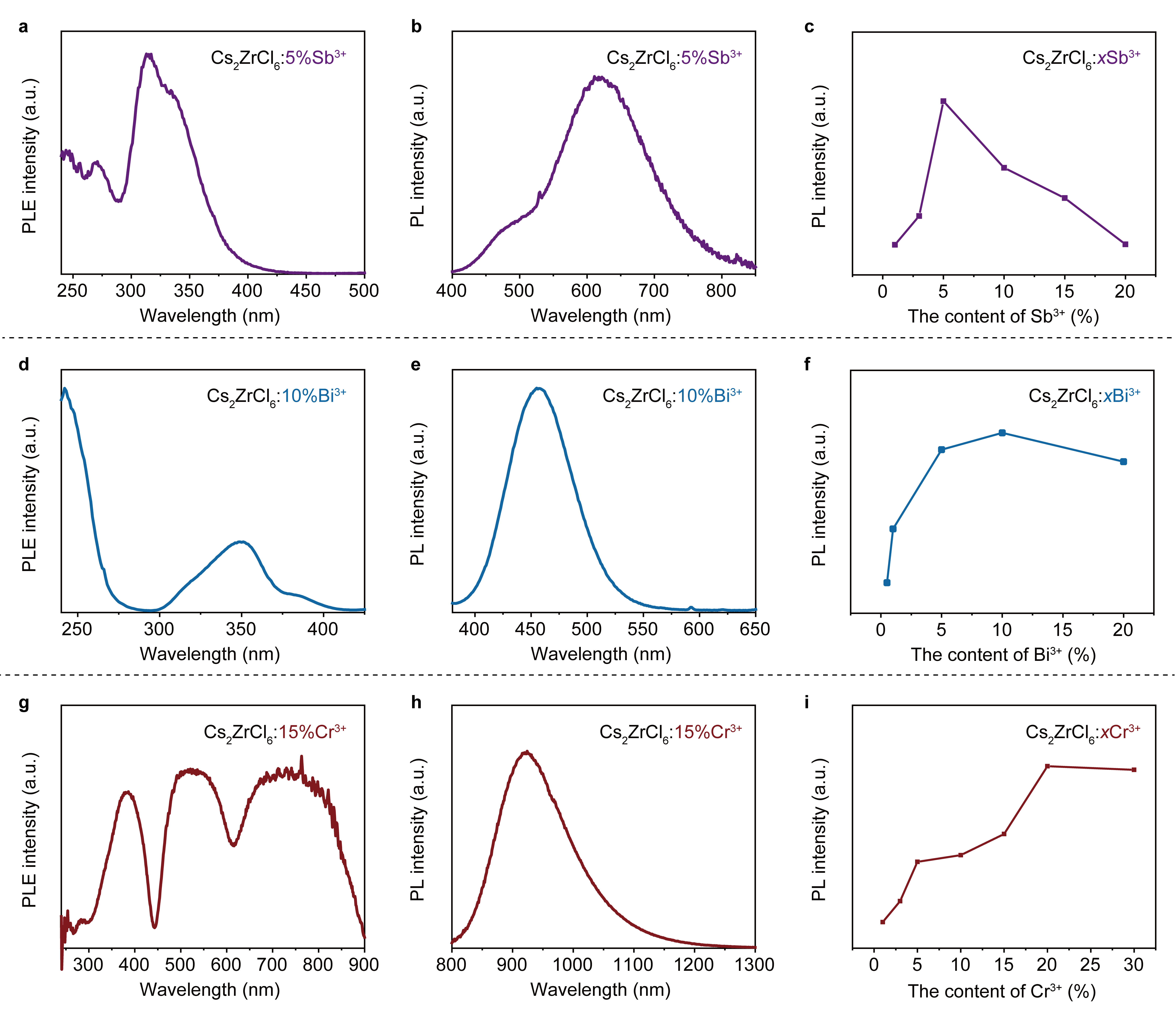
**

**Fig. S41. Ions-doped Cs_2_ZrCl_6_ phosphors.** Luminescence properties of **(a-c)** Sb^3+^, **(d-f)** Bi^3+^ and **(g-i)** Cr^3+^.


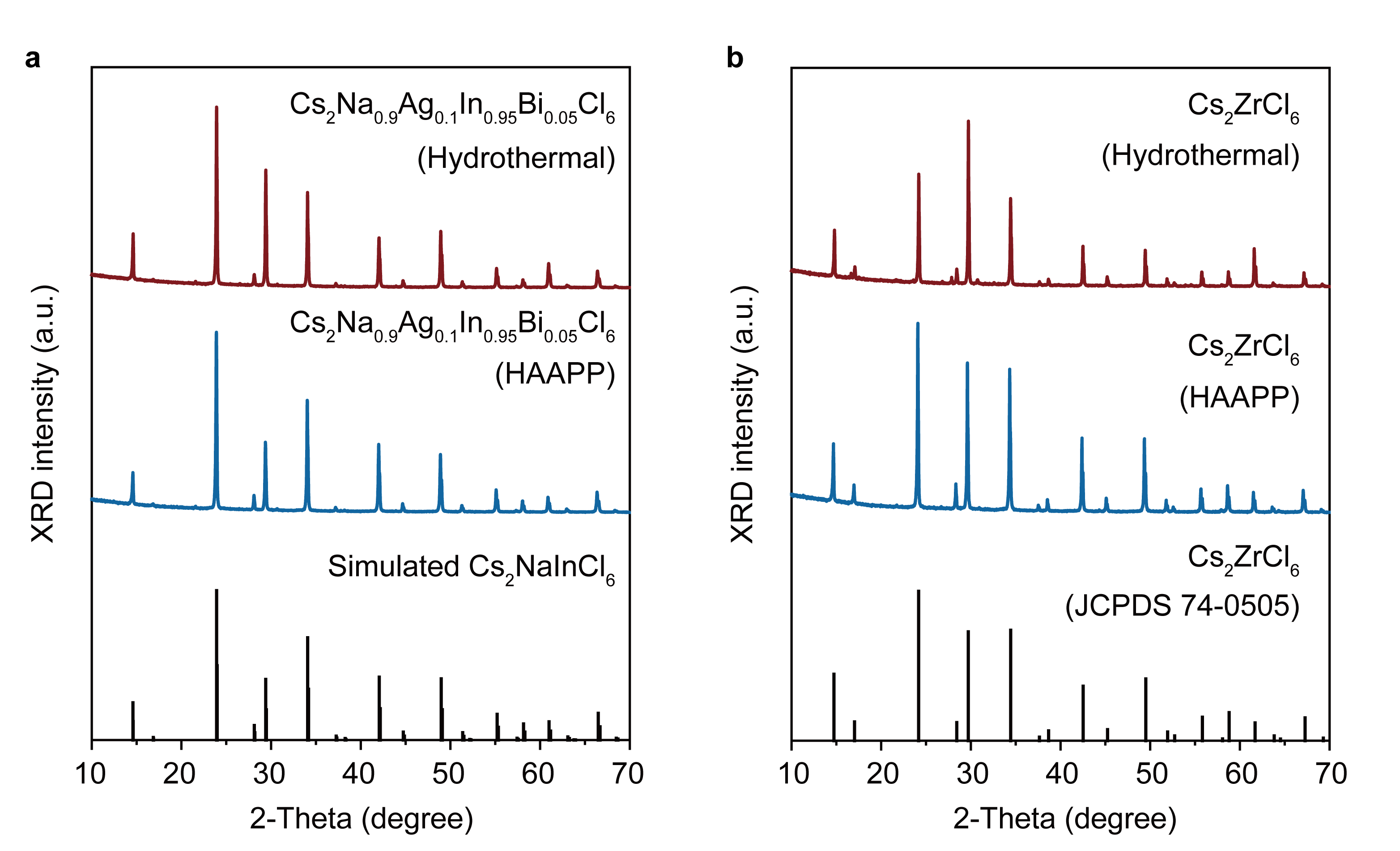


**Fig. S42. XRD patterns comparisons between the HAAPP strategy and hydrothermal method.**

**
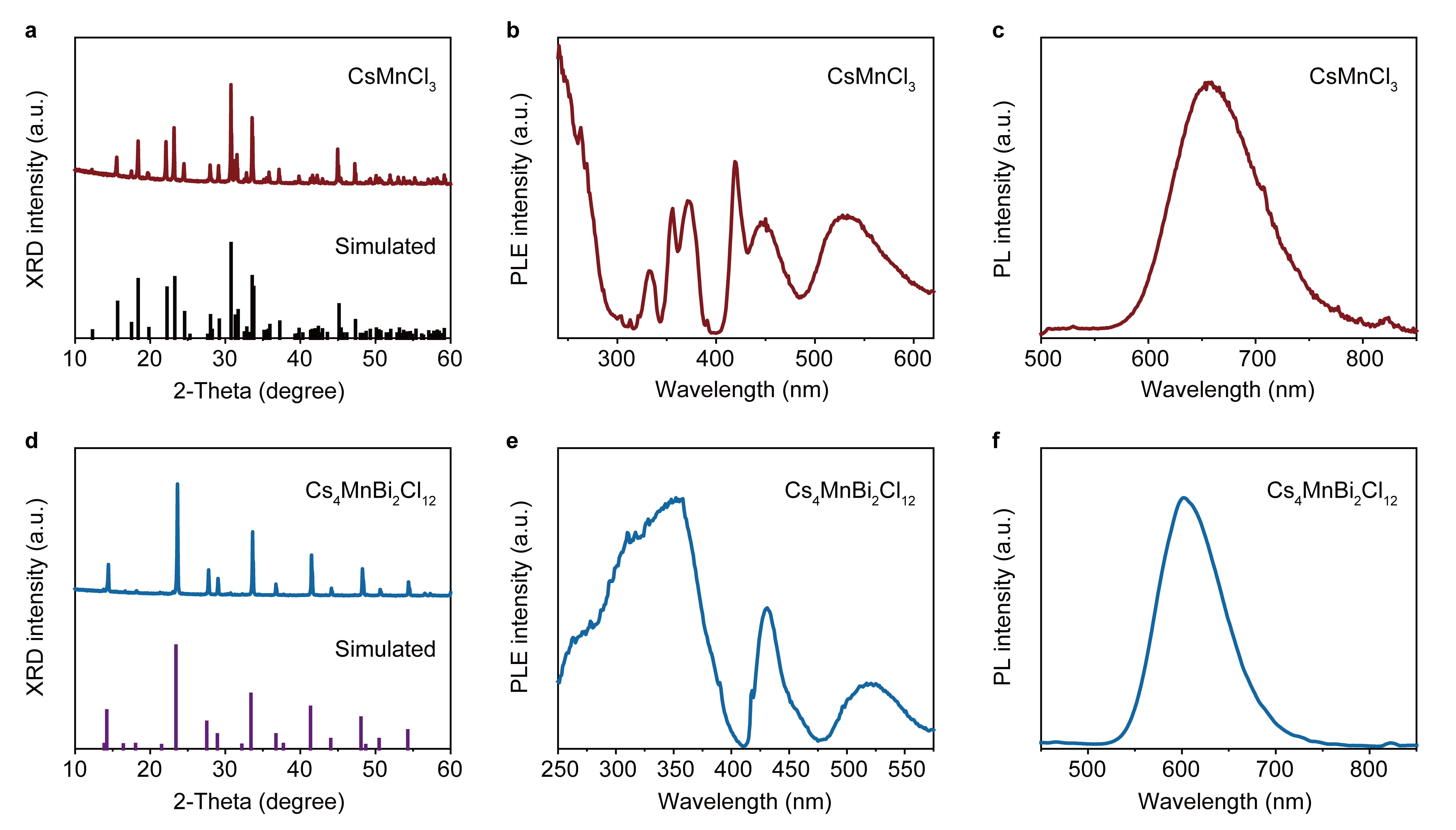
**

**Fig. S43. XRD, PLE and PL spectra of (a-c)** CsMnCl_3_ **and (d-e)** Cs_4_MnBi_2_Cl_12_ products.


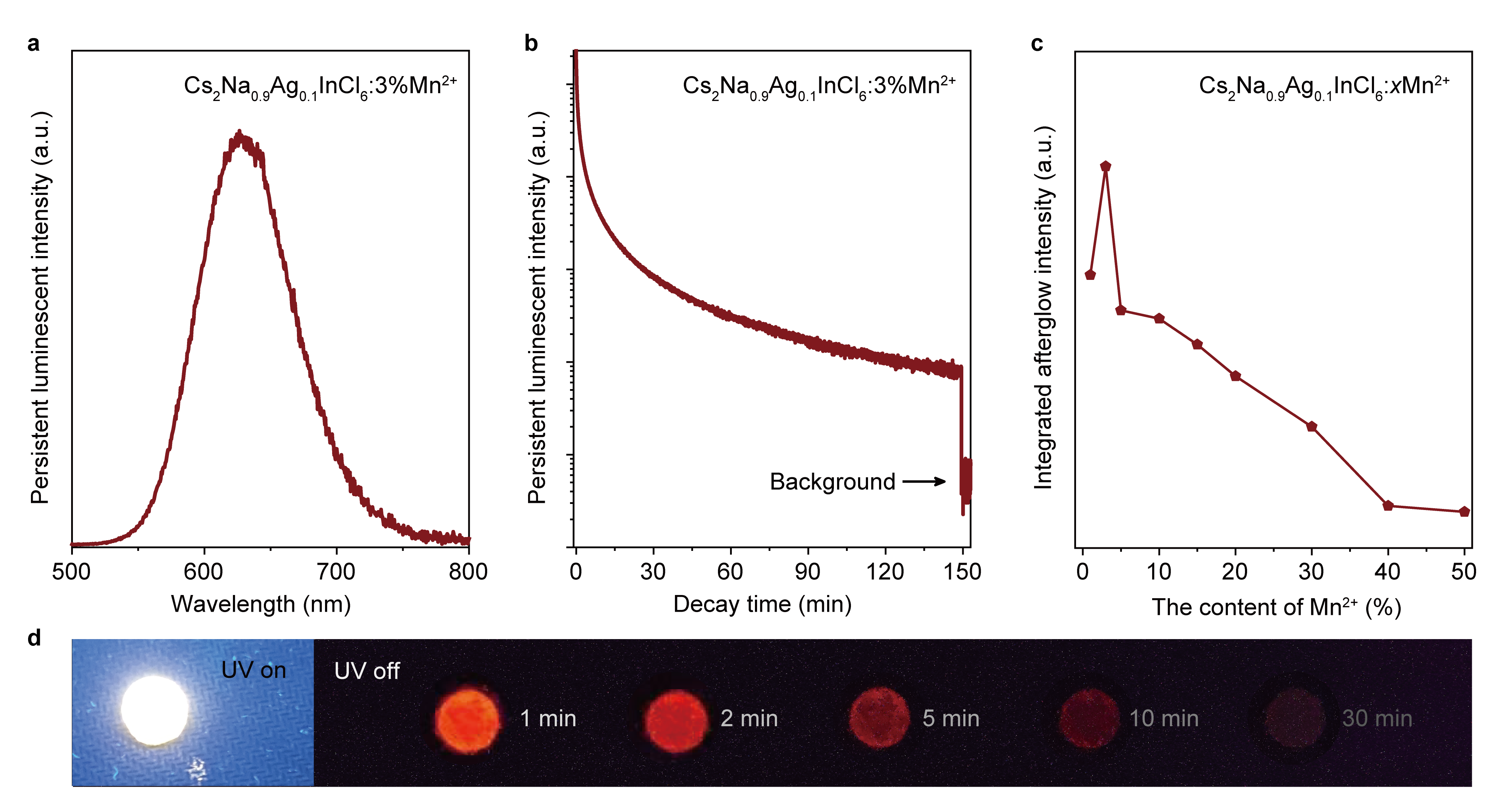


**Fig. S44. Afterglow performance of Cs_2_Na_0.9_Ag_0.1_InCl_6_:Mn^2+^. (a)** Persistent emission spectrum; **(b)** Afterglow decay curve; **(c)** Integrated afterglow intensity as a function of Mn^2+^ content; **(d)** Digital photographs of fluorescence and afterglow.

**
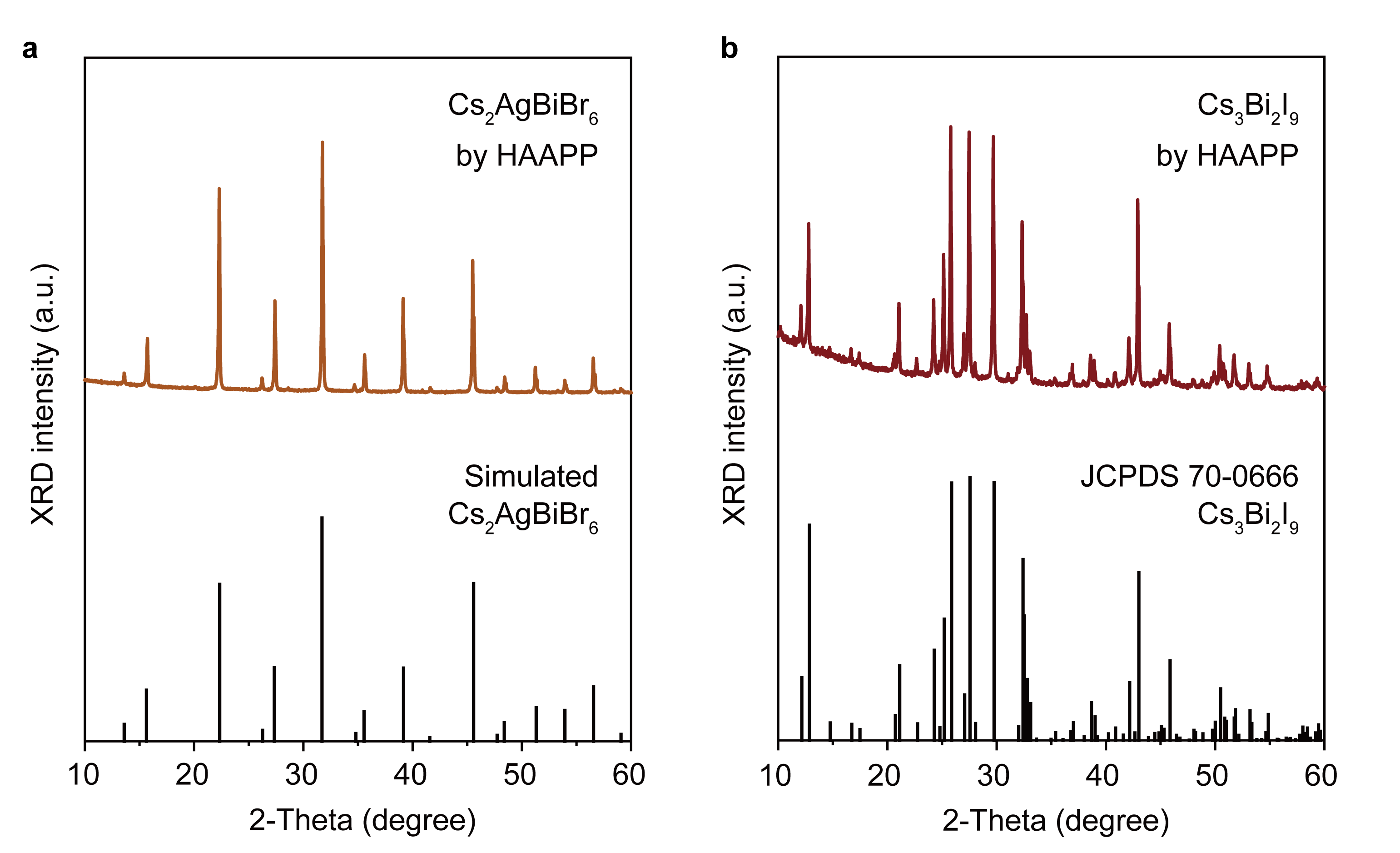
**

**Fig. S45. XRD patterns of (a)** prepared/simulated Cs_2_AgBiBr_6_; **(b)** prepared and standard Cs_3_Bi_2_I_9_ (JCPDS 70-0666).

**Table S1. Actual doping ratios (in molar) of Na and Ag in Cs_2_Na_1-_*_x_*Ag*_x_*InCl_6_ prepared by the HAAPP strategy.**

| **The value of *x*** | **Na** | **Ag** | **Calculated Ag/(Ag+Na)** |
| --- | --- | --- | --- |
| 0.1 | 83.26% | 7.95% | 0.09 |
| 0.3 | 64.30% | 25.68% | 0.29 |
| 0.5 | 48.83% | 33.54% | 0.41 |
| 0.7 | 33.02% | 66.43% | 0.67 |
| 0.9 | 7.78% | 70.20% | 0.90 |

**Table S2. Actual doping ratios (in molar) of Na, Ag, In and Bi in Cs_2_Na_0.9_Ag_0.1_In_1-_*_y_*Bi*_y_*Cl_6_ prepared by the HAAPP strategy.**

| ***y*** | **Na** | **Ag** | **Calculated Ag/(Ag+Na)** | **In** | **Bi** | **Calculated Bi/(In+Bi)** |
| --- | --- | --- | --- | --- | --- | --- |
| 0.05 | 74.1% | 9.9% | 0.12 | 100% | 2.1% | 0.02 |
| 0.1 | 71.3% | 8.9% | 0.11 | 93.6% | 7.6% | 0.08 |
| 0.3 | 73.4% | 8.4% | 0.10 | 80.0% | 26.1% | 0.25 |
| 0.5 | 82.2% | 8.6% | 0.09 | 59.0% | 48.9% | 0.45 |
| 0.7 | 91.7% | 7.1% | 0.07 | 37.4% | 69.0% | 0.65 |
| 0.9 | 93.0% | 12.9% | 0.12 | 12.6% | 89.2% | 0.88 |

**Table S3. Actual doping ratios (in molar) of Na and Ag in Cs_2_Na_0.9_Ag_0.1_In_0.95_Bi_0.05_Cl_6_ prepared by the HAAPP strategy for 5 times in different days.**

| **The value of *x*** | **Na** | **Ag** | **Ag/(Ag+Na)** |
| --- | --- | --- | --- |
| 1_st_ | 83.26% | 7.95% | 0.09 |
| 2_nd_ | 82.84% | 10.13% | 0.11 |
| 3_rd_ | 80.45% | 9.12% | 0.10 |
| 4_th_ | 85.36% | 9.55% | 0.10 |
| 5_th_ | 78.83% | 15.82% | 0.17 |

**Table S4. Actual doping ratios (in molar) of Tb^3+^ in Cs_2_Na_0.9_Ag_0.1_InCl_6_ prepared by the HAAPP strategy.**

| **Feeding ratios of Tb** | **Actual ratios measured by ICP**  **ICP** |
| --- | --- |
| 1% | 0.13% |
| 5% | 0.71% |
| 10% | 1.61% |
| 15% | 1.42% |
| 20% | 1.45% |

**Table S5. Actual doping ratios (in molar) of doping ions in Cs_2_Na_0.9_Ag_0.1_InCl_6_ prepared by the HAAPP strategy.**

| **Feeding ratios** | **Actual ratios measured by ICP**  **ICP** |
| --- | --- |
| 10%Mn | 0.99% |
| 10%Yb | 1.88% |
| 10%Tm | 1.84% |
| 10%Nd | 1.30% |
| 10%Cr | 0.03% |
| 10%Sb | 4.53% |

**Table S6. Lifetime fitting data of Cs_2_Na_1-_*_x_*Ag*_x_*InCl_6_ for Fig. S5c**

| ***x* value** | ***A*_1_ (%)** | ***τ*_1_ (μs)** | ***A*_2_ (%)** | ***τ*_2_ (μs)** | ***A*_3_ (%)** | ***τ*_3_ (μs)** | ***τ*_avg_ (μs)** | ***R*^2^** |
| --- | --- | --- | --- | --- | --- | --- | --- | --- |
| **0** | 79.89 | 0.36 | 20.11 | 11.08 | **-** | **-** | 9.85 | 0.9909 |
| **0.1** | 50.62 | 0.30 | 49.38 | 6.72 | **-** | **-** | 6.44 | 0.9984 |
| **0.2** | 45.39 | 0.27 | 54.61 | 5.50 | **-** | **-** | 5.29 | 0.9978 |
| **0.3** | 20.02 | 0.24 | 77.45 | 3.74 | 2.53 | 16.32 | 5.24 | 0.9989 |
| **0.4** | 19.12 | 0.63 | 77.71 | 2.96 | 3.17 | 13.32 | 4.40 | 0.9989 |
| **0.5** | 13.32 | 0.53 | 83.82 | 2.82 | 2.86 | 13.75 | 4.28 | 0.9991 |
| **0.6** | 28.53 | 0.51 | 68.01 | 2.08 | 3.46 | 9.65 | 3.29 | 0.9994 |
| **0.7** | 91.34 | 1.18 | 8.66 | 6.90 | **-** | **-** | 3.22 | 0.9934 |
| **0.8** | 81.60 | 0.69 | 18.40 | 4.49 | - | - | 2.95 | 0.9943 |

**Table S7. Lifetime fitting data of Cs_2_Na_0.9_Ag_0.1_In_1-_*_y_*Bi*_y_*Cl_6_ for Fig. S8c**

| ***y* value** | ***A*_1_ (%)** | ***τ*_1_ (μs)** | ***A*_2_ (%)** | ***τ*_2_ (μs)** | ***τ*_avg_ (μs)** | ***R*^2^** |
| --- | --- | --- | --- | --- | --- | --- |
| **0** | 71.54 | 5.5 | 28.46 | 13.58 | 9.50 | 0.9978 |
| **0.01** | 81.84 | 6.13 | 18.16 | 16.25 | 9.88 | 0.9985 |
| **0.05** | 81.43 | 5.73 | 18.57 | 17.13 | 10.35 | 0.9981 |
| **0.1** | 78.20 | 5.59 | 21.80 | 17.26 | 10.99 | 0.9978 |
| **0.2** | 79.27 | 5.42 | 20.73 | 16.02 | 10.04 | 0.9986 |
| **0.3** | 81.22 | 5.68 | 18.78 | 16.59 | 10.08 | 0.9982 |
| **0.4** | 76.00 | 4.98 | 24.00 | 15.74 | 10.35 | 0.9985 |
| **0.5** | 78.26 | 4.69 | 21.74 | 15.31 | 9.74 | 0.9982 |
| **0.6** | 73.62 | 4.68 | 26.38 | 15.27 | 10.39 | 0.9985 |
| **0.7** | 76.55 | 4.92 | 23.45 | 15.2 | 9.92 | 0.9987 |
| **0.8** | 77.81 | 4.37 | 22.19 | 14.84 | 9.52 | 0.9986 |
| **0.9** | 72.53 | 3.08 | 27.47 | 11.06 | 7.68 | 0.9985 |
| **1.0** | 72.21 | 2.41 | 27.79 | 9.65 | 6.80 | 0.9983 |

**Table S8. Actual doping ratios (in molar) of doping ions of products prepared in the saturated NaCl solution.**

| **Elements** | **Feeding ratios** | **Actual ratios measured by ICP**  **ICP** |
| --- | --- | --- |
| Ag | 10% | 5.99% |
| Na | 90% | 389.51% |

The actual ratios were calculated based on the pure Cs_2_Na_0.9_Ag_0.1_InCl_6_. The actual content of Na was greatly over 100%, which was caused by the additional precipitation of NaCl impurity from the provided saturated NaCl solution, shown in Fig. S22.

**Table S9. Actual doping ratios (in molar) of doping ions of products prepared in the saturated KCl solution.**

| **Elements** | **Feeding ratios** | **Actual ratios measured by ICP**  **ICP** |
| --- | --- | --- |
| Ag | 10% | 36.54% |
| Na | 90% | 5.97% |
| K | 0% | 570.63% |

The actual ratios were calculated based on the pure Cs_2_Na_0.9_Ag_0.1_InCl_6_. Based on the results in Fig. S22, the products contain Cs_2_Na_1-_*_x-y_*Ag*_x_*K*_y_*InCl_6_ and KCl solids. Similar to the Table. S8, the actual content of K was greatly over 100%, which should be attributed to the alloyed K^+^ in perovskites and additional KCl solids from the provided saturated KCl solution.
